# Cancer risks in rheumatoid arthritis patients who received immunosuppressive therapies: Will immunosuppressants work?

**DOI:** 10.3389/fimmu.2022.1050876

**Published:** 2022-12-20

**Authors:** Yuzhuo Zhang, Jiangpeng Lin, Zhixuan You, Hengjia Tu, Peng He, Jiarong Li, Rui Gao, Ziyu Liu, Zhiyuan Xi, Zekun Li, Yi Lu, Qiyuan Hu, Chenhui Li, Fan Ge, Zhenyu Huo, Guibin Qiao

**Affiliations:** ^1^ Guangzhou Medical University, Guangzhou, Guangdong, China; ^2^ Department of Medical Imaging, Changzhi Medical College, Changzhi, Shanxi, China; ^3^ College of Clinical Medicine, Jilin University, Changchun, Jilin, China; ^4^ Sir Run Run Shaw Hospital, Zhejiang University School of Medicine, Hangzhou, China; ^5^ Shanxi Medical University, Taiyuan, Shanxi, China; ^6^ School of Basic Medical Sciences, Qiqihar Medical University, Qiqihar, Heilongjiang, China; ^7^ National Cancer Center/National Clinical Research Center for Cancer/Cancer Hospital, Chinese Academy of Medical Sciences and Peking Union Medical College, Beijing, China; ^8^ Department of Thoracic Surgery, Guangdong Provincial People’s Hospital, Guangdong Academy of Medical Sciences, Guangzhou, Guangdong, China

**Keywords:** rheumatoid arthritis, cancer risk, immunosuppression therapy, immunosuppressant, tumor mutational burden

## Abstract

**Background:**

Exploring the cancer risks of rheumatoid arthritis (RA) patients with disease-modifying anti-rheumatic drugs (DMARDs) can help detect, evaluate, and treat malignancies at an early stage for these patients. Thus, a comprehensive analysis was conducted to determine the cancer risk of RA patients using different types of DMARDs and analyze their relationship with tumor mutational burdens (TMBs) reflecting immunogenicity.

**Methods:**

A thorough search of PubMed, EMBASE, Web of Science, and Medline was conducted up to 20 August 2022. Standardized incidence ratios (SIRs) were constructed with a random-effect model to determine risks for different types of malignancies in comparison with the general population. We also analyzed the correlation between SIRs and TMBs using linear regression (LR).

**Results:**

From a total of 22 studies, data on 371,311 RA patients receiving different types of DMARDs, 36 kinds of malignancies, and four regions were available. Overall cancer risks were 1.15 (SIR 1.15; 1.09–1.22; *p* < 0.001) and 0.91 (SIR 0.91; 0.72–1.14; *p* = 0.402) in RA populations using conventional synthetic DMARDs (csDMARDs) and biologic DMARDs (bDMARDs), respectively. RA patients taking csDMARDs displayed a 1.77-fold lung cancer risk (SIR 1.77; 1.50–2.09; *p* < 0.001), a 2.15-fold lymphoma risk (SIR 2.15; 1.78–2.59; *p* < 0.001), and a 1.72-fold melanoma risk (SIR 1.72; 1.26–2.36; *p* = 0.001). Correlation coefficients between TMBs and SIRs were 0.22 and 0.29 from those taking csDMARDs and bDMARDs, respectively.

**Conclusion:**

We demonstrated a cancer risk spectrum of RA populations using DMARDs. Additionally, TMBs were not associated with elevated cancer risks in RA patients following immunosuppressive therapy, which confirmed that iatrogenic immunosuppression might not increase cancer risks in patients with RA.

**Interpretation:**

Changes were similar in cancer risk after different immunosuppressive treatments, and there was a lack of correlation between SIRs and TMBs. These suggest that we should look for causes of increased risks from the RA disease itself, rather than using different types of DMARDs.

## Introduction

RA is a chronic persistent autoimmune illness with an uncertain etiology (1). It is characterized by progressive joint injury and extra-articular manifestations, resulting in various clinical symptoms and injuries, even causing permanent disability (2). The global prevalence of RA is 0.27% according to the 2017 Global Burden of Disease Study. The age-standardized prevalence of RA in North America and Western Europe reaches 0.38 and 0.35%, respectively. The prevalence is also higher in India and South American countries (3). In addition, its impact on the female population is particularly severe (4). Over the past few decades, the improvement of the standard of RA treatment has improved the response rate and the quality of life for RA patients. However, RA populations’ rising rates of comorbidity and related mortality from cardiovascular disease, infections, and cancers have gained the attention of the public (5–9).

Patients with RA were frequently treated with DMARDs, including csDMARDs and bDMARDs, to relieve the condition (10). These drugs have a slow onset but provide sustained remission of disease activity in patients, fundamentally inhibiting progressive damage to tissues and joints and delaying or halting disease progression (4, 11). DMARDs usually have an immunosuppressive effect and may cause immunosuppression in the human body. However, because immunological disorders are a common symptom of RA, it is unclear how the immune system will respond to csDMARDs and bDMARDs. This has raised concerns about the immune status of RA patients who received immunosuppressive therapies, including infectious burden, vaccination, and malignancy burden (9, 12, 13).

In the past decades, a growing number of studies have focused on the incidence or prevalence of cancer in RA populations. A comprehensive analysis conducted by Simon et al. demonstrated that elevated SIRs of overall cancer in RA patients and SIRs of site-specific cancers were different (14). In solid tumors, lung cancer showed an elevated risk (15), whereas breast cancer demonstrated a decreased risk (16). Increased lymphoma risk was a common finding in hematologic neoplasms (17). All of these suggested that cancer risks were organ specific. However, this organ specificity of cancer was not well interpreted by available studies.

TMB is defined as the utilization of sequencing technology to detect the total number of somatic cell gene coding errors, base replacements, gene insertions, or deletion errors per million bases (18). TMB and cancer type diversity indicates differences in immunogenicity, which is directly tied to the immune system’s capacity to identify tumor cells. TMB may be associated with site-specific cancer risks in RA patients after using immunosuppressants, while the underlying cause is uncertain.

Here, we performed a comprehensive analysis to determine the overall cancer risk of RA patients using various immunosuppressant therapies, as well as the risk of particular site-specific cancers. In addition, we utilized LR to investigate the correlation between corresponding SIRs and TMBs on participants with distinct baseline characteristics. The aims of this study were to explore whether immunosuppressive therapy increased cancer risks in RA populations and to compare characteristics of association through a synthetic analysis of the relationship between different patients and thus to understand how immune system functions better.

## Methods

### Search strategy and selection criteria

We conducted a search of PubMed, EMBASE, Web of Science, and Medline databases up to 1 August 2022. Studies that involved RA patients receiving immunosuppressive therapy were qualified. References from pertinent papers were also checked. When crucial information was lacking, we asked the authors for further information. All search results were assessed in accordance with the PRISMA statement (19). The protocol was registered in the Prospective Register of Systematic Reviews (PROSPERO ID CRD42021232432).

### Study selection criteria

Our analysis comprised studies that satisfied the following criteria: (1) only case–control studies and cohort studies were included, (2) documented at least one site-specific cancer risk in RA patients receiving immunosuppressive therapies, and (3) published or accepted English-language studies can be retrieved from the web database described above, as of August 2022. Studies were excluded for the following reasons: (1) studies not published in English or not retrievable *via* the aforementioned network databases, (2) SIRs and 95% CIs could not be determined or calculated from the paper, and (3) a deficiency of statistics based on available data.

### Data extraction and quality assessment

Three authors (YZ, JL, and PH) independently extracted relevant data meeting the inclusion criteria and addressed any disputes through discussion. The following demographic and clinical characteristics of patients were extracted as outcome data: first author, publication year, region, age characteristic, type of immunosuppressive therapy drugs, number of RA patients who received immunosuppressive therapies, number of studies on various cancers, and SIRs in various cancers after immunosuppressive treatment was recorded.

Using CGP tests, Chalmers et al. (18) found the distribution of TMB in 100,000 cancer cases in a heterogeneous cohort study and confirmed that TMB was associated with somatic alterations in more than 100 different types of tumors. Chalmers et al. (18) also established pertinent median TMB values for tumors in different organs (**Supplementary Table S1**). If the median TMB for a particular tumor was not available, it was calculated by averaging TMB values of subtypes that were included in the study (NHL, pancreas, ovary, small intestine, brain and central nervous system, colorectum, soft tissue, urethra, skin, and lung). The detected median TMB and their natural logarithms are displayed in **Supplementary Tables S1**, **S2**.

The Newcastle–Ottawa Scale (NOS) criteria (20) were used to assess the methodological quality of selected trials, including selection (four items), comparability (one item), and outcome (three items), presented in **Supplementary Table S3**. Any disputes were settled through consultation.

### Statistical analysis

We extracted SIRs published in each study and their 95% CIs and pooled cancer risks of RA patients who received immunosuppressive therapy (csDMARDs or bDMARDs). A randomized effect model was employed to synthesize SIRs and 95% CIs in RA patients receiving immunosuppressive therapy (21, 22). The synthesized SIRs were divided into six modules by anatomical site or histology: respiratory system cancers, hematological malignancies, cutaneous system cancers, reproductive and urinary organ system cancers, digestive system cancers, and other malignancies. Cochran’s *Q* test and the *I* (2) statistic were adopted to assess the heterogeneity across studies (23). When the *I* (2) statistic >50%, statistical heterogeneity was deemed significant (23). In addition, subgroup analyses by geographic region (North America, Europe, Oceania, and Asia) and age (55 and 60 years served as cutoff points) were undertaken to assess possible correlations between included studies’ varied features. To determine if any study had a substantial impact on the outcomes, pooled-effect estimates were put through sensitivity analyses by removing each study. Publication biases were evaluated statistically by running Funnel plot tests and Egger’s test (24).

Moreover, we utilized the LR method to determine correlation coefficients and to investigate connections between TMBs and comprehensive SIRs. Since neither TMBs nor SIRs were normally distributed, we used the natural logarithm of each variable to compare them. For statistical and LR analysis, STATA 15.0 software (STATA Corp, College Station, TX, USA) was applied. Every *p*-value had a two-tailed distribution, and a *p*-value of 0.05 or lower was regarded as statistically significant.

### Role of the funding source

Funders were not involved in decisions regarding study design, data collection, data synthesis and analysis, manuscript writing, or submission of articles for publication.

## Results

### Systematic search and study characteristics

**Figure 1:**
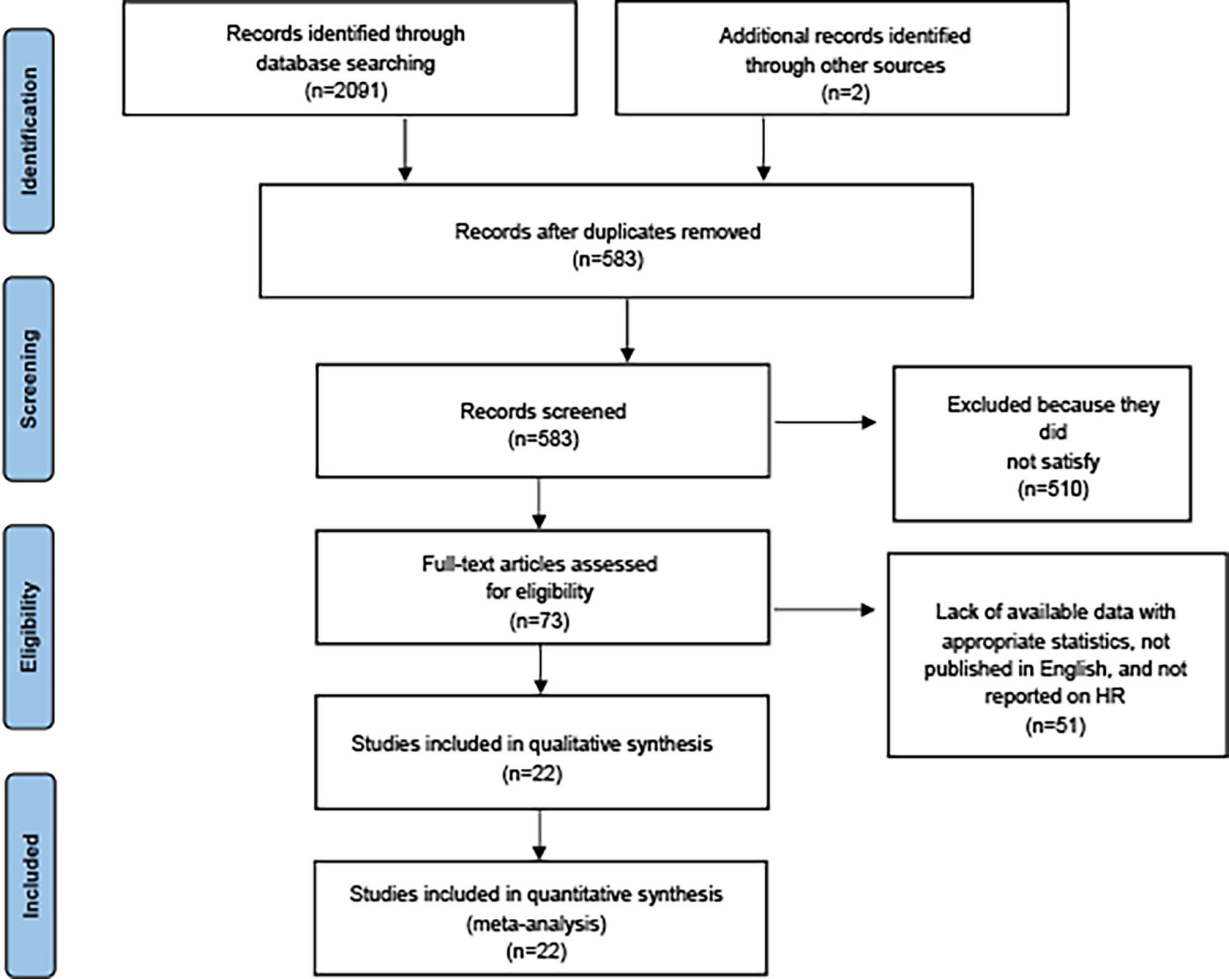
PRISMA diagram of study selection (available at https://prisma-statement.org/prismastatement/flowdiagram.aspx).

We identified 2,091 studies through database search, in addition to identifying two additional studies by other sources. After removing duplicates, 583 studies remained. Ultimately, 22 studies (15, 25–39) met the requirements for inclusion, which involved a total of 371,311 RA patients receiving immunosuppressive therapy and covered 36 types of site-specific cancers. **Supplementary Table S3** provides more information on the included studies. Among these studies, 17 studies (15, 17, 25, 26, 28, 29, 31–34, 39–42) provided SIRs of multiple cancers in RA patients receiving immunosuppressive therapies [7 for multiple DMARDs (15, 25, 29, 31, 39, 41, 43), 5 for csDMARDs (17, 28, 33, 38, 42), and 5 for bDMARDs (26, 32, 34, 37, 40)]. The remaining five studies (27, 30, 35, 36, 44) provided SIRs for several or individual cancer risks of RA patients who received immunosuppressive therapies (**Figure 1**).

### Cancer risks in RA patients who received immunosuppressive therapies

Firstly, in comparison with the general population, RA patients treated with csDMARDs had a 1.15-fold increased cancer risk (SIR 1.15; 1.09–1.22; *p* < 0.001). In addition, some elevated significant site-specific cancer risks were shown, such as lung cancer (SIR 1.77; 1.50–2.09; *p* < 0.001), lymphoma (SIR 2.15; 1.78–2.59; *p* < 0.001), melanoma (SIR 1.72; 1.26–2.36; *p* = 0.001), and NMSC (SIR 1.50; 1.10-2.06; *p* = 0.011). Secondly, RA patients taking bDMARDs did not have obvious increased cancer risks than the general population. However, among these patients, lymphoma (SIR 4.19; 2.51-7.00; *p* < 0.001) and NMSC (SIR 1.61; 1.34-1.96; *p* < 0.001) demonstrated raised cancer risks. SIRs of each site-specific cancer for csDMARDs or bDMARDs are listed in **Table 1**.

**Table 1:** SIRs of all-cancer and cancer types by anatomical site or histology among RA patients with different DMARDs.

Site	csDMARDs					bDMARDs							
	*N*	SIR	95% CI	*p*-value	*I (2*)	*N*	SIR	95% CI	*p*-value	I-square			
**Overall cancer**	13	1.15	(1.09, 1.22)	<0.001	79.7%	9	0.91	(0.72, 1.14)	0.402	82.4%		
Respiratory system													
Lung cancer	11	1.77	(1.50, 2.09)	<0.001	72.6%	5	1.20	(0.84, 1.73)	0.315	52.4%		
Hematological malignancies													
Lymphoma	8	2.15	(1.78, 2.59)	<0.001	35.9%	7	4.19	(2.51, 7.00)	<0.001	74.5%		
Non-Hodgkin’s lymphoma	9	2.04	(1.71, 2.44)	<0.001	47.5%	4	2.63	(1.18, 5.86)	0.018	73.7%		
Hodgkin’s lymphoma	5	3.17	(2.56, 3.94)	<0.001	0.0%	3	6.11	(2.11, 17.72)	0.001	0.0%		
Leukemia	4	1.96	(1.18, 3.25)	0.009	69.4%	1	0.75	(0.04, 15.33)	NA	–		
Multiple myeloma	2	1.94	(1.36, 2.77)	<0.001	0.0%	1	1.68	(0.42, 6.74)	NA			
Integumentary system													
Non-melanoma skin cancer	6	1.50	(1.1, 2.06)	0.011	76.0%	8	1.61	(1.34, 1.94)	<0.001	61.7%		
Melanoma	6	1.72	(1.26, 2.36)	0.001	69.2%	1	1.57	(0.70, 3.49)	NA			
Squamous cell carcinoma	2	1.63	(1.02, 2.60)	0.041	52.8%	3	1.42	(1.21, 1.66)	<0.001	0.0%		
Basal cell carcinoma	1	1.22	(1.06, 1.40)	0.005		2	1.05	(0.74, 1.49)	0.788	72.0%		
Reproductive and urinary organs													
Breast cancer	8	0.84	(0.79, 0.91)	<0.001	0.0%	3	0.56	(0.42, 0.74)	<0.001	0.0%		
Prostate cancer	6	0.93	(0.79, 1.10)	0.426	37.6%	3	0.80	(0.47, 1.36)	0.405	6.2%		
Bladder cancer	5	1.27	(0.87, 1.86)	0.212	84.1%	2	0.83	(0.45, 1.53)	0.545	0.0%		
Cervical cancer	5	1.49	(0.98, 2.28)	0.064	78.0%	2	1.04	(0.69, 1.58)	0.845	0.0%		
Renal cancer	4	1.36	(1.05, 1.75)	0.02	63.1%	2	0.98	(0.63, 1.53)	0.945	0.0%		
Cancer of uterus Endometrial	4	0.53	(0.40, 0.69)	<0.001	20.3%	2	0.48	(0.30, 0.76)	0.002	0.0%		
ovarian cancer	2	0.85	(0.31, 2.33)	0.75	88.0%	2	1.48	(0.79, 2.76)	0.222	26.2%		
Cancer of vulva and vagina	2	2.00	(0.80, 4.96)	0.136	0.0%	1	0.80	(0.10, 5.80)	NA	–		
Cancer of testis	2	1.29	(0.28, 6.05)	0.745	0.0%	–	–	–	–	–		
Cancer of urethra	1	1.12	(0.54, 2.34)	0.763	–	1	1.31	(0.68, 2.52)	NA	–		
Digestive system													
Colorectal cancer	8	0.80	(0.6, 1.07)	0.138	84.5%	3	0.93	(0.38, 2.26)	0.869	89.4%		
Gastric cancer	6	1.05	(0.71, 1.55)	0.825	55.9%	1	0.85	(0.32, 1.49)	NA	–		
Liver cancer	4	1.13	(0.93, 1.36)	0.221	0.0%	1	2.10	(0.50, 8.40)	NA	–		
Pancreas cancer	3	0.90	(0.73, 1.10)	0.291	0.0%	2	0.65	(0.26, 1.59)	0.345	34.2%		
Esophageal cancer	3	1.45	(1.10, 1.90)	0.007	0.0%	1	0.85	(0.32, 1.49)	NA	–		
Cancer of small intestine	1	1.22	(0.75, 1.89)	NA	–	–	–	–	–	–		
Gallbladder cancer	1	2.00	(0.30, 14.40)	NA		–	–	–	–	–		
Cancer of bile duct	–	–	–	–	–	1	0.69	(0.30, 1.15)	NA	–		
Anus cancer	–	–	–	–	–	1	2.50	(0.60, 10.00)	NA	–		
Other malignancies													
Cancer of soft tissues	2	1.42	(1.00, 2.01)	0.05	0.0%	–	–	–	–	–		
Brain and central nervous system cancer	1	1.12	(0.89, 1.40)	0.327	–	2	0.81	(0.12, 5.22)	0.821	65.9%		
Oral cancer	1	1.12	(0.68, 1.72)	NA	–	3	1.26	(0.38, 4.19)	0.705	67.3%		
Head and neck cancer	1	0.30	(0.20, 0.70)	<0.001	–	–	–	–	–	–		
Cancer of bones and joints	1	5.70	(2.16, 15.03)	<0.001		–	–	–	–	–		
Cancer of larynx	1	1.19	(0.63, 2.04)	–	–	–	–	–	–	–		
Thyroid cancer	–	–	–	–	–	1	1.32	(0.53, 2.24)	NA			

An SIR >1 suggests that the cancer risk is higher than that of the ordinary population. SIR, standardized incidence ratio; CI, confidence interval.

N refers to the total number of SIR values of corresponding site-specific cancers in RA patients with different immunosuppressants.

### Relationships between cancer incidence and TMB

**Figure 2:**
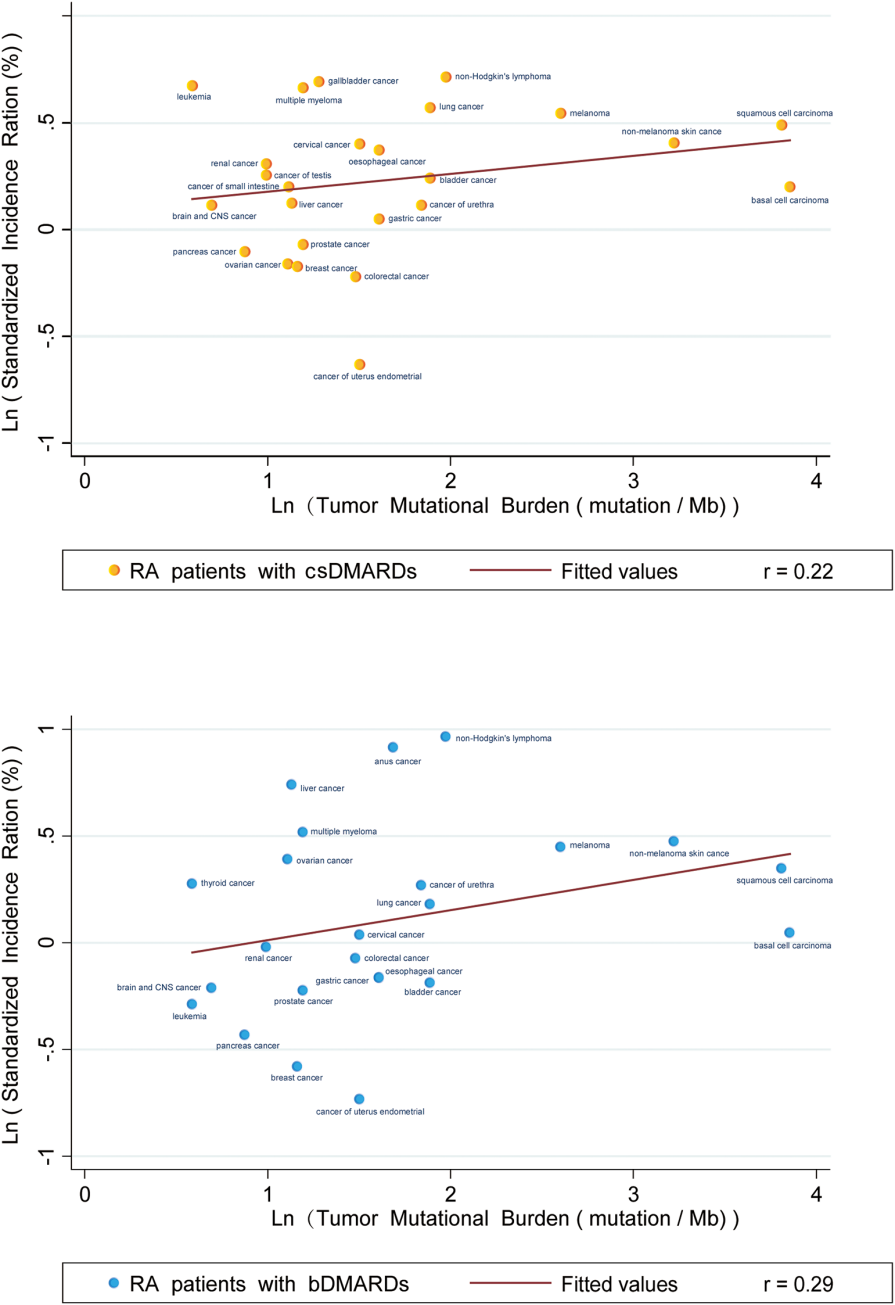
Correlation between Tumor Mutational Burdens and Standardized Incidence Ratios in RA patients taking CSDMARDs or bDMARDs. Data on the x and y axis are shown on a logarithmic scale.

We found no evident correlation between TMBs and SIRs in RA patients who received csDMARDs (*p* < 0.001) or bDMARDs (*p* < 0.001). Correlation coefficients between SIRs and TMBs were 0.22 and 0.29, respectively, in RA patients using csDMARDs or bDMARDs (**Figure 2**).

### Sensitivity analysis

Results of sensitivity analyses are displayed in **Supplementary Figures S2**, **S3**. Our findings demonstrated that pooled outcomes for RA patients utilizing csDMARDs were not greatly changed by excluding any one trial. However, there were some differences in results for RA patients using bDMARDs, which is possibly caused by the limited quantity of included studies.

### Subgroup analyses

We conducted exploratory subgroup analyses by region and age. The results of the subgroup analyses revealed that *I*² values decreased when stratified by region in included studies, indicating that region was the source of apparent heterogeneity in our study. However, we found no considerable reduction in heterogeneity in each group in exploratory subgroup analyses using the cutoff ages of 55 and 60 years. **Supplementary Figure S4** presented forest plots of subgroup analyses.

### Publication bias

Owing to the limited characteristics collected in RA patients, we could not detect sources other than region and age that contributed to manifest heterogeneity. Our findings of Egger’s test showed no publication bias for all cancers examined in RA patients taking csDMARDs. However, publication bias existed in several cancers for RA patients using bDMARDs, which is possibly induced by the small number of included studies (**Supplementary Figure S5**). Furthermore, we created funnel plots to evaluate bias, and the outcomes are displayed in **Supplementary Figure S6**.

## Discussion

Our study presented the profile of overall and site-specific cancer risks in RA patients using immunosuppressive therapy in contrast to the general population. Subgroup analysis revealed an obvious reduction in overall cancer risk after immunosuppressive therapy when divided by geographic region. However, the difference in overall cancer risk after immunosuppressive therapy was not reduced when stratified by age (55 and 60 years, respectively), suggesting that age of onset of RA patients may not be the main source of heterogeneity in our study. For the relationship between SIRs and TMBs, the correlation coefficient was 0.22 (csDMARDs) and 0.29 (bDMARDs), indicating that varied cancer incidence in RA patients was extremely weakly related to the use of immunosuppressants, and the two could be considered uncorrelated.

Overall cancer risk was increased in RA patients using csDMARDs, while those using bDMARDs did not indicate the same result. Furthermore, lung cancer risk was increased in RA patients using csDMARDs. It is more interesting to note that those receiving csDMARDs or bDMARDs both had high risks of having hematological and integumentary system cancer.

According to estimates, smoking caused up to 85% of lung cancer cases (45). Tobacco use also had been found to enhance RA risk by 40% (46). Therefore, smoking might be a common risk factor for lung cancer and RA in certain populations (47). Previous meta-analyses (14, 48) have reported that RA may be one of the risk factors for lung cancer. A pooled analysis and Mendelian randomization study (49) found that RA was associated with a 44% increased risk of lung cancer (RR 1.44; 1.31–1.57). Some recently published studies were included in our study, which has a larger sample size and greater statistical power. Our findings suggested that RA patients using DMARDS also had a significantly higher risk of having lung cancer. In addition, pulmonary involvement in RA is the most common extra-articular manifestation, occurring in up to 60% (50–53). RA-related interstitial lung disease is the most challenging complication of lung manifestation (54). It leads to pathological epithelial damage, repair abnormalities, and epithelial–mesenchymal transformation, which may eventually lead to cancerous transformation (55).

Although the exact mechanism by which RA altered lung cancer risk is still uncertain, there have been several molecular pathways underlying RA and lung cancer. The cyclooxygenase (COX)-2/thromboxane A2 (TxA2) pathway has been demonstrated to play a potential role in lung cancer development through an auto-regulatory feedback loop (56). MAP4K3 (also known as GLK) is a serine/threonine kinase that directly interacts with and activates PKCu, resulting in the activation of IKK/NF-kB in human T cells (57). Overexpression of GLK in T cells is a key causative factor in the development of autoimmune diseases (58). Meanwhile, in cancer cells, GLK directly phosphorylates and activates IQGAP1, resulting in the induction of Cdc42-mediated cell migration and cancer metastasis (59). In summary, the bridge from RA to lung cancer still needs to be studied further.

In previous studies on RA (60–62) versus cancer, incidence of blood malignancies showed high-risk results, which is consistent with our findings. In 2005, Zintzaras et al. explored the correlation between RA and NHL through a pooled analysis of observational studies (63). They described that the SIR of RA with lymphoma was 3.9 (95% CI: 2.5–5.9). Our study confirmed the results of Zintzaras and colleagues. Moreover, we further concluded that RA patients treated with csDMARDs or bDMARDs had a 2.15-fold and 4.19-fold risk of lymphoma, respectively. Notably, RA itself, a representative chronic inflammatory disease, was one of the triggers for the generation of lymphoma. Conversion of lymphocytes into malignant clones was a multi-step process involving chronic stimulation with various antigens and/or viruses, such as Epstein–Barr virus, local inflammation, and immunosuppression. These could weaken immune protection and increase the risk of lymphoma (64, 65). As for integumentary system cancers, previous studies (15, 17, 27, 29, 31, 66–68) have indicated that RA patients using csDMARDs and bDMARDs had a 20%–80% increased risk of NMSC in contrast to the general population. In our study, non-melanoma risk increased in RA patients using csDMARDs and bDMARDs, as were melanoma risk in RA patients using csDMARDs. However, the number of studies using bDMARDs for RA alone was insufficient, and its relation with melanoma risk remains to be confirmed.

TMB is a promising biomarker that can be used to predict the efficacy of tumors on ICIs (18). In 2017, Elizabeth Marion Jaffee et al. (69) plotted a linear plot between the median TMB of 27 tumors and the ORR of ICI treatment (analytical data came from multiple clinical studies), in which TMB was positively correlated to ORR (*p* < 0.001) and the correlation coefficient was 0.74. Additionally, to a certain degree, TMB reflects immunogenicity. TMB refers to the number of somatic mutations after the tumor genome removes the germline mutation; a large number of mutated somatic cells will produce a large number of altered peptides, some of which are successfully expressed and processed by the major histocompatibility complex, thereby producing new antigens recognized by the human immune system, which can cause anti-tumor responses to mutated somatic cells through specific killing. This makes mutated somatic cells more likely to be targeted by activated immune cells (18). Therefore, when the immune system is in a normal state, malignant tumor cells with high TMB values are more easily recognized and cleared by the body’s immune system during the embryonic stage. However, the use of immunosuppressants diminishes the role of the human immune system in immune surveillance. This results in an increased survival rate of malignant tumors with high TMB values, which may ultimately result in higher rates of overall and site-specific cancers in a segment of the population.

In 2020, Huo et al. (70) undertook a comprehensive study of cancer risks for recipients of solid organ transplantation and then plotted a linear plot between TMBs of site-specific malignancies and cancer combined SIRs of solid organ transplant recipients. They found a remarkable positive relationship between TMBs and SIRs (*p* < 0.001) with a correlation coefficient of 0.68, indicating that an increase in cancer incidence was related to immunosuppression. Our study also used LR to explore the correlation between TMB and the corresponding cancer incidence. However, our study found that both RA patients treated with csDMARDs and bDMARDs had low coefficients (csDMARDs *r* = 0.22, bDMARDs *r* = 0.29) of correlation between TMB and the corresponding cancer incidence line. This partly demonstrated that the risk of organ-specific cancer in RA patients was not related to DMARDs use, which reminded us to explore the potential mechanism of cancer risks from the RA disease itself. Additionally, some immunomodulatory agents used in the clinical treatment of different autoimmune disorders have been shown to increase Treg frequency and susceptibility to Treg suppression and to restore the balance between immunosuppression and immune activation, so as to achieve the purpose of treatment (71–76). Our pooled analysis and correlation analysis of TMBs and SIRs preliminarily indicated that changes of cancer risks in RA patients were not primarily dependent on immunosuppressive effects after DMARDs. However, exact factors contributing to changes in cancer risks are still uncertain.

The three main strengths of our study should be emphasized. First and foremost, to the best of our knowledge, this study is the most pooled study in the world to evaluate cancer risks in RA patients using different kinds of immunosuppressants. Secondly, the study explored the relationship between SIRs of corresponding malignant tumors and their TMBs, investigated the cancer risk of RA patients from a specific immunological perspective through fitting analysis, and clarified that changes in cancer risks were not due to the use of DMARDs. Lastly, previously published meta-analyses were restricted to specific organs, specific malignancies, individual countries, individual regions, or small sample sizes. The study gathered a large sample of data from around the world and assessed cancer risks in RA patients treated with DMARDs. These global data, along with our findings, can provide a strong reference for clinicians and researchers on the risks of immunosuppressive therapy in patients with RA, as well as reliable evidence-based medical evidence for primary prevention of certain cancers in RA patients.

Despite the above advantages of our study, there are some limitations to this comprehensive analysis. First, there is a strong heterogeneity between involved studies, which may be due to the following reasons: (1) differences in tumor characteristics of malignancies were incorporated; (2) few studies provided detailed information on BMI (77), smoking status (78), alcohol consumption (79), and immunosuppressant drug dosages to adjust for these potential confounding factors; and (3) reference materials of included studies are all general populations, but countries and regions of studies are different, resulting in different final matching criteria. Therefore, we conducted subgroup analyses based on the populations’ age included in the study as well as regional distribution to improve stability of results, but the study is still only a preliminary observation of cancer risks in RA patients with immunosuppressant therapy. Second, the involved studies have used different age and sex ratios for the standardization of SIR, which may have some effect on fitting results. Third, due to the limited number of studies using bDMARDs, there was a publication bias in sensitivity analysis and Egger’s test for certain site-specific cancers. Fourth, the TMB algorithm used in our study was relatively basic, and findings should be interpreted with caution. Because we cannot directly use TMB in the study of Chalmers et al. (18), we replaced TMB by averaging cancer subtypes mentioned in that study. Therefore, in order to increase the reliability of analysis associations, more precise TMBs should be determined according to the proportion of tumor pathological typing. Last but not least, most studies did not specify the dosage of DMARDs used, limiting our ability to explain whether potential adverse effects caused by patient medications impacted cancer risks in RA patients through subgroup analysis.

The number of studies on the risk of cancers after DMARDs is far from adequate. Thus, future iterations of the evaluation for cancer risks require more relevant clinical observations. In addition, in subsequent clinical evidence, we also hope that more studies would record the timing of cancer onset, specific drug names and doses, duration of drug treatment, and final efficacy in detail, which would provide more evidence for further syntheses. In the later stages, we will continue to pay attention to new research in this field and strive to collect more adequate and specific data, aiming to demonstrate a better cancer risk landscape for RA patients.

## Conclusion

Comprehensive analysis indicated that RA patients using DMARDs (only csDMARDs or bDMARDs, not suitable for csDMARDs combined with bDMARDs) had elevated risks of lung and renal cancer, but reduced risks of breast and endometrial cancer.

The higher risks were also shown in cancers of the hematologic and cutaneous system (different malignancies at different risks). In addition, TMBs were not associated with elevated cancer risks in RA patients following immunosuppressive therapy. These relationships provided clinicians guidance on the individualized treatment of RA patients and the prevention of site-specific malignancies.

## Data availability statement

The original contributions presented in the study are included in the article/**Supplementary Material**. Further inquiries can be directed to the corresponding authors.

## Ethics statement

This study has been approved by Institutional Review Board (IRB) of Guangdong Provincial People’s Hospital. Because the study is a retrospective analysis, the ethics committee waived informed consent for all patients.

## Author contributions

This study was proposed by YZ. YZ, JRL and PH extracted data. YZ, RGR and ZX analyzed data. YZ wrote the initial draft manuscript. JPL and PH revised the original text and carried out English editing. ZH and GQ revised the final manuscript. All authors have contributed to the revision of the manuscript. YZ is the guarantor. All authors contributed to the article and approved the submitted version.
