# Supplementary material for: Cancer risks in rheumatoid arthritis patients who received immunosuppressive therapies: Will immunosuppressants work?
Source: Front Immunol. 2022 Dec 20;13:1050876. doi: 10.3389/fimmu.2022.1050876 (PMC9807750; doi:10.3389/fimmu.2022.1050876)
Supplement: Supplementary file 1 [file DataSheet_1.pdf]

Cancer risks in rheumatoid arthritis patients received immunosuppressive therapies:will immunosupressants work?

Yuzhuo Zhang, Jiangpeng Lin, Zhixuan You, Hengjia Tu, Peng He,

Jiarong Li, Rui Gao, Ziyu Liu, Zhiyuan Xi, Zekun Li, Yi Lu, Qiyuan Hu,

Chenhui Li, Fan Ge, Zhenyu Huo, Guibin Qiao

Supplementary materials

**Table of Contents**		
**Figure S1**	PRISMA checklist.	**3-6**
**Figure S2**	Sensitivity analysis for cancers in RA patients with csDMARDs.	**7-13**
**Figure S3**	Sensitivity analysis for cancers in RA patients with bDMARDs.	**14-18**
**Figure S4**	Subgroup analyses, stratified by region and age.	**19-23**
**Figure S5**	Egger's test plots for cancers in RA patients with csDMARDs.	**24-30**
**Figure S6**	Egger's test plots for cancers in RA patients with bDMARDs.	**31-35**
**Figure S7**	Funnel plots for cancers in RA patients with csDMARDs.	**36-44**
**Figure S8**	Funnel plots for cancers in RA patients with bDMARDs.	**45-52**
**Table S1**	Tumor Mutational Burden values and their natural logarithms of directly matched malignancies.	**53**
**Table S2**	Tumor Mutational Burden values and their natural logarithms of non-directly matched malignancies.	**54-55**
**Table S3**	Demographicdetailsoftheincludedstudies.	**56-58**

PRISMA 2020 Checklist

Section and Topic	Item #	Checklist item	Location where item is reported
**TITLE**			
Title	1	Identify the report as a systematic review.	Page 1 (Title)
**ABSTRACT**			
Abstract	2	See the PRISMA 2020 for Abstracts checklist.	Page 4 (ABSTRACT)
**INTRODUCTION**			
Rationale	3	Describe the rationale for the review in the context of existing knowledge.	Page 7 (INTRODUCTION)
Objectives	4	Provide an explicit statement of the objective(s) or question(s) the review addresses.	Page 8 (INTRODUCTION)
**METHODS**			
Eligibility criteria	5	Specify the inclusion and exclusion criteria for the review and how studies were grouped for the syntheses.	Page 9 (Study election criteria)
Information sources	6	Specify all databases, registers, websites, organisations, reference lists and other sources searched or consulted to identify studies. Specify the date when each source was last searched or consulted.	Page 8 (Search strategy and selection criteria)
Search strategy	7	Present the full search strategies for all databases, registers and websites, including any filters and limits used.	Page 8 (Search strategy and selection criteria)
Selection process	8	Specify the methods used to decide whether a study met the inclusion criteria of the review, including how many reviewers screened each record and each report retrieved, whether they worked independently, and if applicable, details of automation tools used in the process.	Page 9 (Data extraction and quality assessment)
Data collection process	9	Specify the methods used to collect data from reports, including how many reviewers collected data from each report, whether they worked independently, any processes for obtaining or confirming data from study investigators, and if applicable, details of automation tools used in the process.	Page 9,10 (Data extraction and quality assessment)
Data items	10a	List and define all outcomes for which data were sought. Specify whether all results that were compatible with each outcome domain in each study were sought (e.g. for all measures, time points, analyses), and if not, the methods used to decide which results to collect.	Page 9,10 (Data extraction and quality assessment)
	10b	List and define all other variables for which data were sought (e.g. participant and intervention characteristics, funding sources). Describe any assumptions made about any missing or unclear information.	Page 11 (Role of the Funding Source)
Study risk of bias assessment	11	Specify the methods used to assess risk of bias in the included studies, including details of the tool(s) used, how many reviewers assessed each study and whether they worked independently, and if applicable, details of automation tools used in the process.	Page 10,11 (Statistical

Section and Topic	Item #	Checklist item	Location where item is reported
			analysis)
Effect measures	12	Specify for each outcome the effect measure(s) (e.g. risk ratio, mean difference) used in the synthesis or presentation of results.	Page 10 (Data extraction and quality assessment)
Synthesis methods	13a	Describe the processes used to decide which studies were eligible for each synthesis (e.g. tabulating the study intervention characteristics and comparing against the planned groups for each synthesis (item #5)).	Page 9 (Data extraction and quality assessment)
	13b	Describe any methods required to prepare the data for presentation or synthesis, such as handling of missing summary statistics, or data conversions.	Page 10,11 (Statistical analysis)
	13c	Describe any methods used to tabulate or visually display results of individual studies and syntheses.	Table 1
	13d	Describe any methods used to synthesize results and provide a rationale for the choice(s). If meta-analysis was performed, describe the model(s), method(s) to identify the presence and extent of statistical heterogeneity, and software package(s) used.	Page 10,11 (Statistical analysis)
	13e	Describe any methods used to explore possible causes of heterogeneity among study results (e.g. subgroup analysis, meta-regression).	Page 10,11 (Statistical analysis)
	13f	Describe any sensitivity analyses conducted to assess robustness of the synthesized results.	Page 11 (Statistical analysis)
Reporting bias assessment	14	Describe any methods used to assess risk of bias due to missing results in a synthesis (arising from reporting biases).	Page 11 (Statistical analysis)
Certainty assessment	15	Describe any methods used to assess certainty (or confidence) in the body of evidence for an outcome.	Page 10 (Data extraction and quality assessment)
**RESULTS**			
Study selection	16a	Describe the results of the search and selection process, from the number of records identified in the search to the number of studies included in the review, ideally using a flow diagram.	Page 11,12 (Systematic search and study characteristics)
	16b	Cite studies that might appear to meet the inclusion criteria, but which were excluded, and explain why they were excluded.	Page 12 (Figure 1)
Study characteristics	17	Cite each included study and present its characteristics.	Page 11,12 (Systematic search and study characteristics)

PRISMA 2020 Checklist

Section and Topic	Item #	Checklist item	Location where item is reported
Risk of bias in studies	18	Present assessments of risk of bias for each included study.	Page 15,16 (Publication bias)
Results of individual studies	19	For all outcomes, present, for each study: (a) summary statistics for each group (where appropriate) and (b) an effect estimate and its precision (e.g. confidence/credible interval), ideally using structured tables or plots.	Table 1
Results of syntheses	20a	For each synthesis, briefly summarise the characteristics and risk of bias among contributing studies.	Page 15,16 (Sensitivity analysis, Publication bias)
	20b	Present results of all statistical syntheses conducted. If meta-analysis was done, present for each the summary estimate and its precision (e.g. confidence/credible interval) and measures of statistical heterogeneity. If comparing groups, describe the direction of the effect.	Page 12 (Systematic search and study characteristics)
	20c	Present results of all investigations of possible causes of heterogeneity among study results.	Page 15 (Subgroup analyses)
	20d	Present results of all sensitivity analyses conducted to assess the robustness of the synthesized results.	Page 15 (Sensitivity analysis)
Reporting biases	21	Present assessments of risk of bias due to missing results (arising from reporting biases) for each synthesis assessed.	Page 15,16 (Publication bias)
Certainty of evidence	22	Present assessments of certainty (or confidence) in the body of evidence for each outcome assessed.	Page 12 (Systematic search and study characteristics)
**DISCUSSION**			
Discussion	23a	Provide a general interpretation of the results in the context of other evidence.	Page 16,17,18
	23b	Discuss any limitations of the evidence included in the review.	Page 20,21
	23c	Discuss any limitations of the review processes used.	Page 21
	23d	Discuss implications of the results for practice, policy, and future research.	Page 19,20
**OTHER INFORMATION**			
Registration and protocol	24a	Provide registration information for the review, including register name and registration number, or state that the review was not registered.	Page 6 （SYSTEMATIC REVIEW REGISTRATION）
	24b	Indicate where the review protocol can be accessed, or state that a protocol was not prepared.	Page 6 （SYSTEMATIC

PRISMA 2020 Checklist

Section and Topic	Item #	Checklist item	Location where item is reported
			REVIEW REGISTRATION
	24c	Describe and explain any amendments to information provided at registration or in the protocol.	
Support	25	Describe sources of financial or non-financial support for the review, and the role of the funders or sponsors in the review.	Page 20,21 (Funding)
Competing interests	26	Declare any competing interests of review authors.	Page 22 (Confict of interest)
Availability of data, code and other materials	27	Report which of the following are publicly available and where they can be found: template data collection forms; data extracted from included studies; data used for all analyses; analytic code; any other materials used in the review.	

From: Page MJ, McKenzie JE, Bossuyt PM, Boutron I, Hoffmann TC, Mulrow CD, et al. The PRISMA 2020 statement: an updated guideline for reporting systematic reviews. BMJ 2021;372:n71. doi: 10.1136/bmj.n71

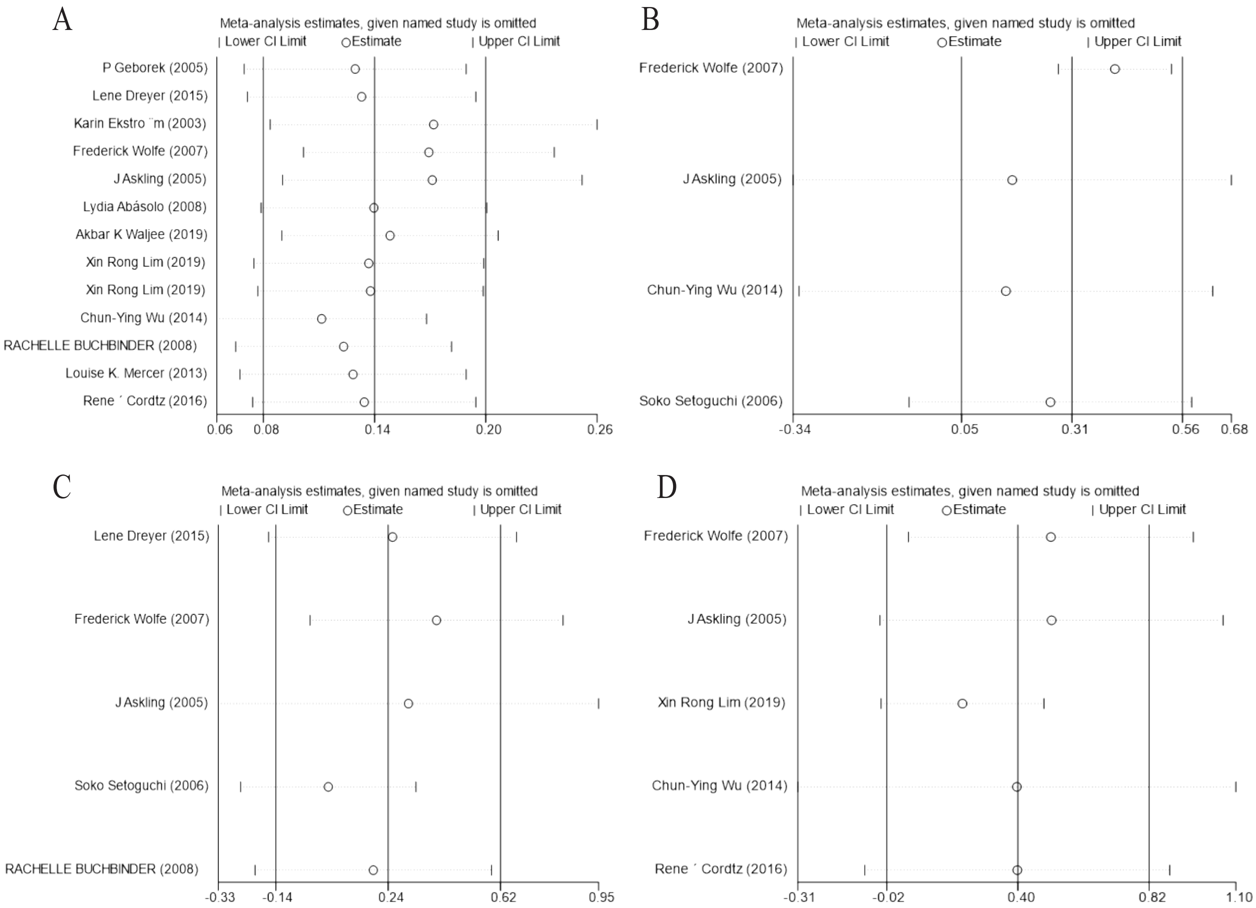
A
Meta-analysis estimates, given named study is omitted
Lower CI Limit
Estimate
Upper CI Limit
P Geborek (2005)
Lene Dreyer (2015)
Karin Ekstro ¨m (2003)
Frederick Wolfe (2007)
J Askling (2005)
Lydia Abásolo (2008)
Akbar K Waljee (2019)
Xin Rong Lim (2019)
Xin Rong Lim (2019)
Chun-Ying Wu (2014)
RACHELLE BUCHBINDER (2008)
Louise K. Mercer (2013)
Rene ´ Cordtz (2016)
0.06
0.08
0.14
0.20
0.26
B
Meta-analysis estimates, given named study is omitted
Lower CI Limit
Estimate
Upper CI Limit
Frederick Wolfe (2007)
J Askling (2005)
Chun-Ying Wu (2014)
Soko Setoguchi (2006)
-0.34
0.05
0.31
0.56
0.68
C
Meta-analysis estimates, given named study is omitted
Lower CI Limit
Estimate
Upper CI Limit
Lene Dreyer (2015)
Frederick Wolfe (2007)
J Askling (2005)
Soko Setoguchi (2006)
RACHELLE BUCHBINDER (2008)
-0.33
-0.14
0.24
0.62
0.95
D
Meta-analysis estimates, given named study is omitted
Lower CI Limit
Estimate
Upper CI Limit
Frederick Wolfe (2007)
J Askling (2005)
Xin Rong Lim (2019)
Chun-Ying Wu (2014)
Rene ´ Cordtz (2016)
-0.31
-0.02
0.40
0.82
1.10

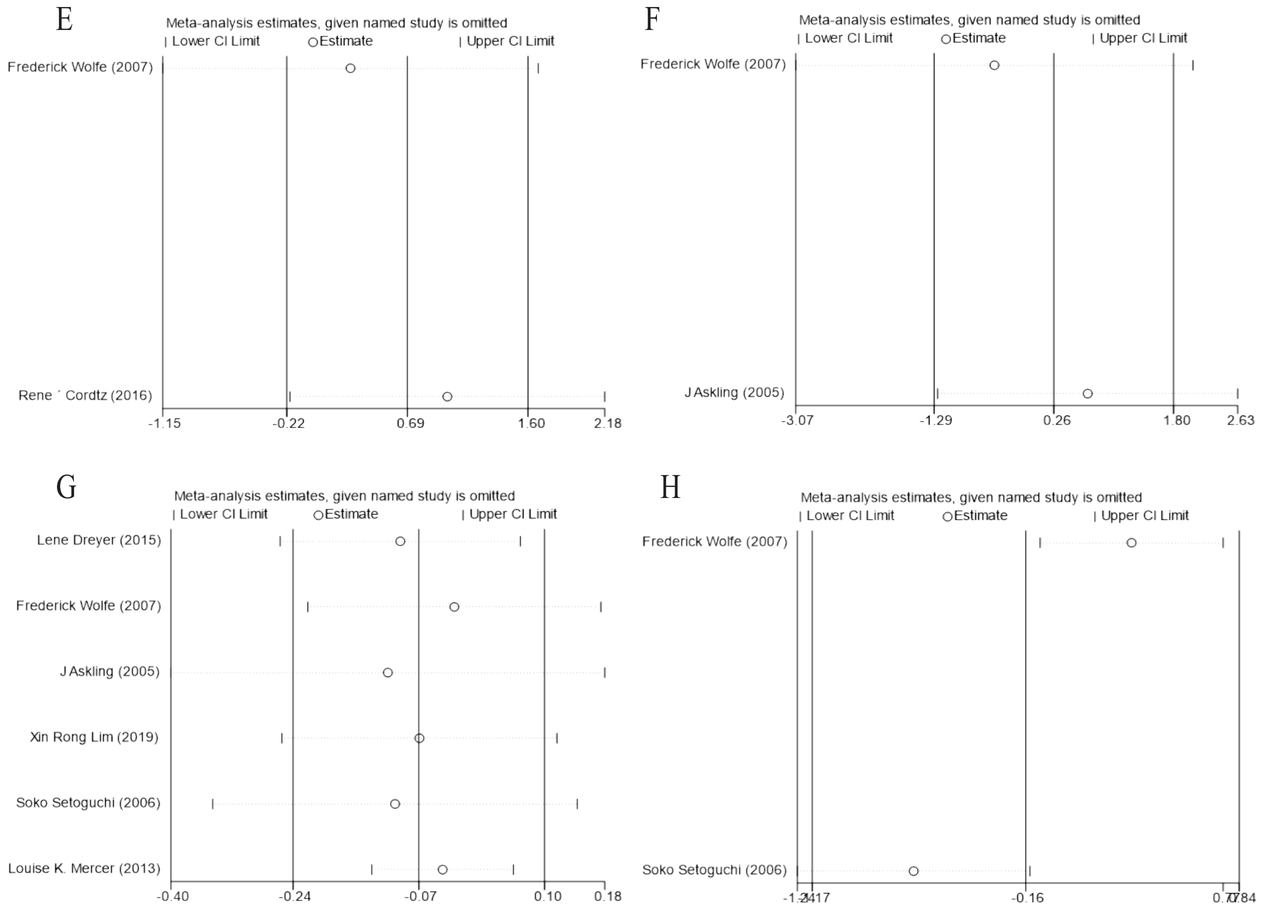
E
Meta-analysis estimates, given named study is omitted
Lower CI Limit
Estimate
Upper CI Limit
Frederick Wolfe (2007)
Rene ´ Cordtz (2016)
-1.15
-0.22
0.69
1.60
2.18
F
Meta-analysis estimates, given named study is omitted
Lower CI Limit
Estimate
Upper CI Limit
Frederick Wolfe (2007)
J Askling (2005)
-3.07
-1.29
0.26
1.80
2.63
G
Meta-analysis estimates, given named study is omitted
Lower CI Limit
Estimate
Upper CI Limit
Lene Dreyer (2015)
Frederick Wolfe (2007)
J Askling (2005)
Xin Rong Lim (2019)
Soko Setoguchi (2006)
Louise K. Mercer (2013)
-0.40
-0.24
-0.07
0.10
0.18
H
Meta-analysis estimates, given named study is omitted
Lower CI Limit
Estimate
Upper CI Limit
Frederick Wolfe (2007)
Soko Setoguchi (2006)
-0.16

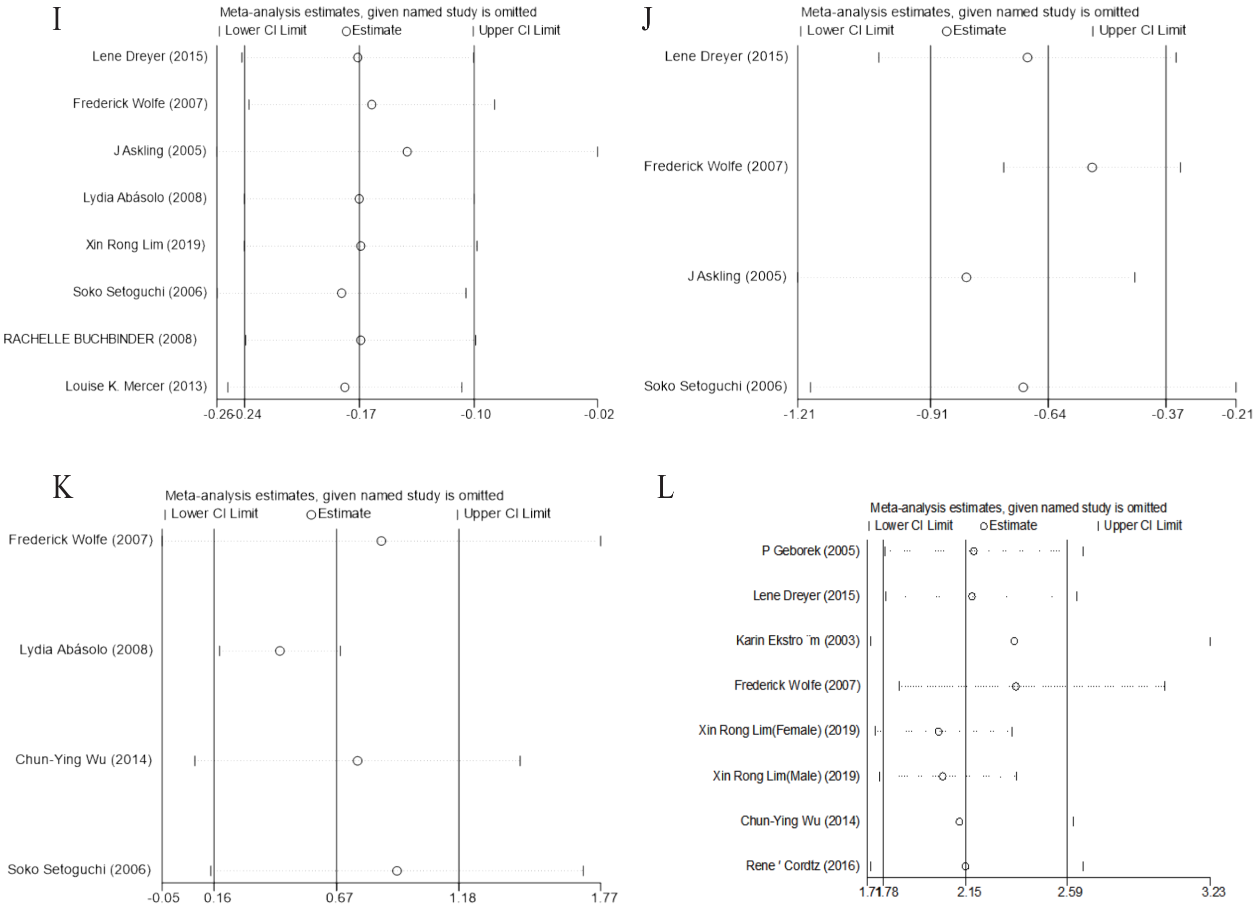

I
Meta-analysis estimates, given named study is omitted
Lower CI Limit
Estimate
Upper CI Limit
Lene Dreyer (2015)
Frederick Wolfe (2007)
J Askling (2005)
Lydia Abásolo (2008)
Xin Rong Lim (2019)
Soko Setoguchi (2006)
RACHELLE BUCHBINDER (2008)
Louise K. Mercer (2013)
-0.26
-0.24
-0.17
-0.10
-0.02
J
Meta-analysis estimates, given named study is omitted
Lower CI Limit
Estimate
Upper CI Limit
Lene Dreyer (2015)
Frederick Wolfe (2007)
J Askling (2005)
Soko Setoguchi (2006)
-1.21
-0.91
-0.64
-0.37
-0.21
K
Meta-analysis estimates, given named study is omitted
Lower CI Limit
Estimate
Upper CI Limit
Frederick Wolfe (2007)
Lydia Abásolo (2008)
Chun-Ying Wu (2014)
Soko Setoguchi (2006)
-0.05
0.16
0.67
1.18
1.77
L
Meta-analysis estimates, given named study is omitted
Lower CI Limit
Estimate
Upper CI Limit
P Geborek (2005)
Lene Dreyer (2015)
Karin Ekstro ̈m (2003)
Frederick Wolfe (2007)
Xin Rong Lim(Female) (2019)
Xin Rong Lim(Male) (2019)
Chun-Ying Wu (2014)
Rene ' Cordtz (2016)
1.71
1.78
2.15
2.59
3.23

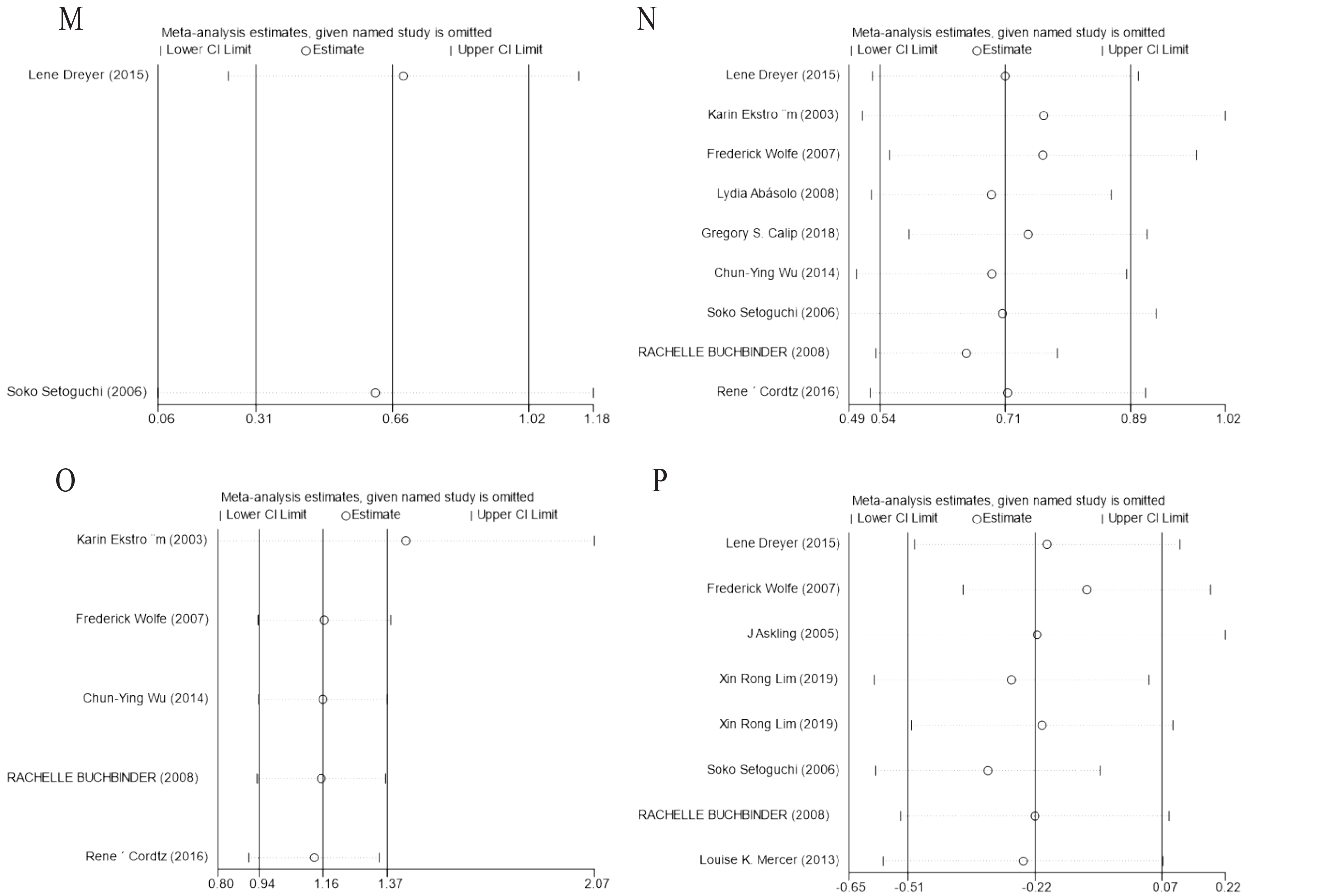
M
Meta-analysis estimates, given named study is omitted
Lower CI Limit
Estimate
Upper CI Limit
Lene Dreyer (2015)
Soko Setoguchi (2006)
0.06
0.31
0.66
1.02
1.18
N
Meta-analysis estimates, given named study is omitted
Lower CI Limit
Estimate
Upper CI Limit
Lene Dreyer (2015)
Karin Ekstro ¨m (2003)
Frederick Wolfe (2007)
Lydia Abásolo (2008)
Gregory S. Calip (2018)
Chun-Ying Wu (2014)
Soko Setoguchi (2006)
RACHELLE BUCHBINDER (2008)
Rene ´ Cordtz (2016)
0.49
0.54
0.71
0.89
1.02
O
Meta-analysis estimates, given named study is omitted
Lower CI Limit
Estimate
Upper CI Limit
Karin Ekstro ¨m (2003)
Frederick Wolfe (2007)
Chun-Ying Wu (2014)
RACHELLE BUCHBINDER (2008)
Rene ´ Cordtz (2016)
0.80
0.94
1.16
1.37
2.07
P
Meta-analysis estimates, given named study is omitted
Lower CI Limit
Estimate
Upper CI Limit
Lene Dreyer (2015)
Frederick Wolfe (2007)
J Askling (2005)
Xin Rong Lim (2019)
Xin Rong Lim (2019)
Soko Setoguchi (2006)
RACHELLE BUCHBINDER (2008)
Louise K. Mercer (2013)
-0.65
-0.51
-0.22
0.07
0.22

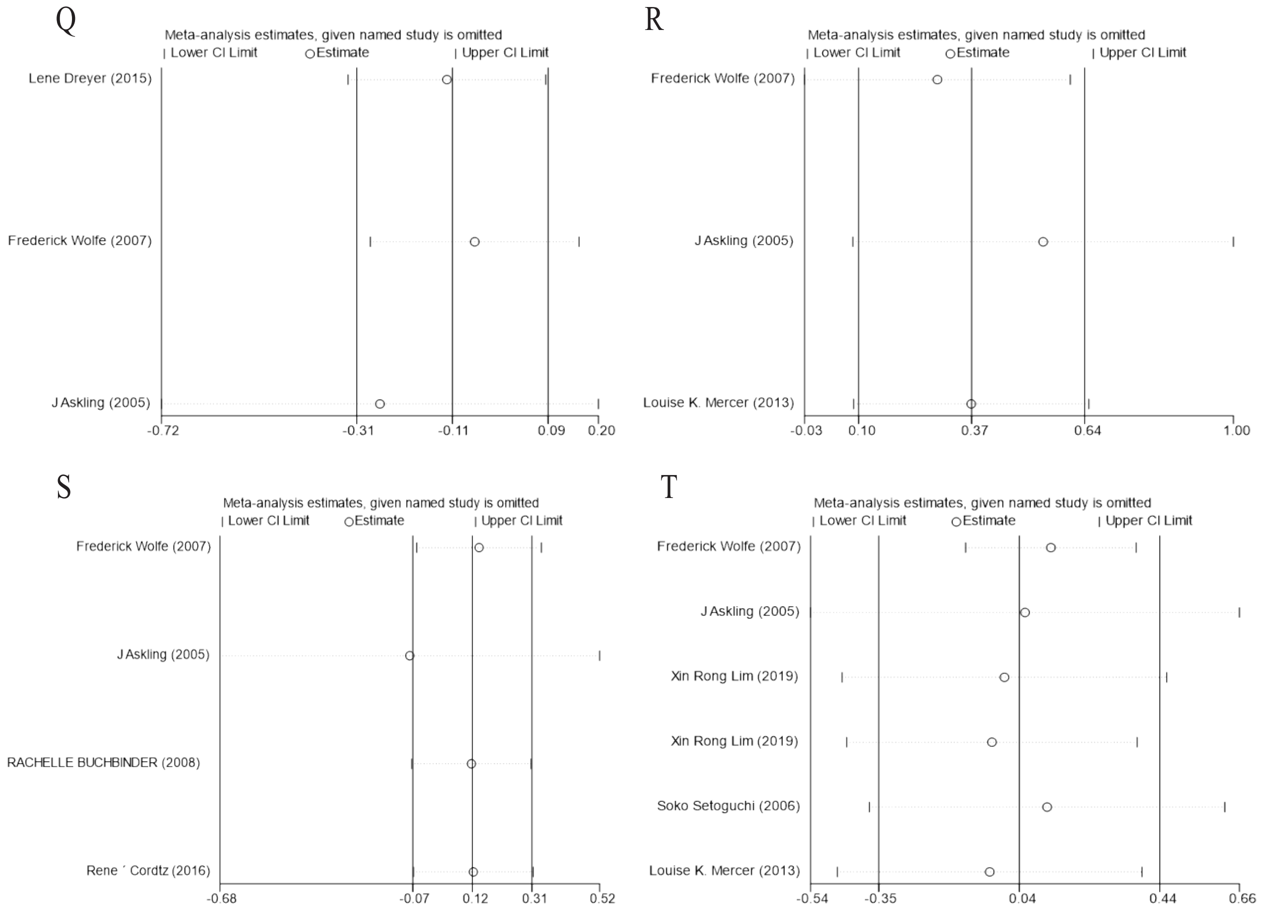
Q
Meta-analysis estimates, given named study is omitted
Lower CI Limit
Estimate
Upper CI Limit
Lene Dreyer (2015)
Frederick Wolfe (2007)
J Askling (2005)
-0.72
-0.31
-0.11
0.09
0.20
R
Meta-analysis estimates, given named study is omitted
Lower CI Limit
Estimate
Upper CI Limit
Frederick Wolfe (2007)
J Askling (2005)
Louise K. Mercer (2013)
-0.03
0.10
0.37
0.64
1.00
S
Meta-analysis estimates, given named study is omitted
Lower CI Limit
Estimate
Upper CI Limit
Frederick Wolfe (2007)
J Askling (2005)
RACHELLE BUCHBINDER (2008)
Rene ´ Cordtz (2016)
-0.68
-0.07
0.12
0.31
0.52
T
Meta-analysis estimates, given named study is omitted
Lower CI Limit
Estimate
Upper CI Limit
Frederick Wolfe (2007)
J Askling (2005)
Xin Rong Lim (2019)
Xin Rong Lim (2019)
Soko Setoguchi (2006)
Louise K. Mercer (2013)
-0.54
-0.35
0.04
0.44
0.66

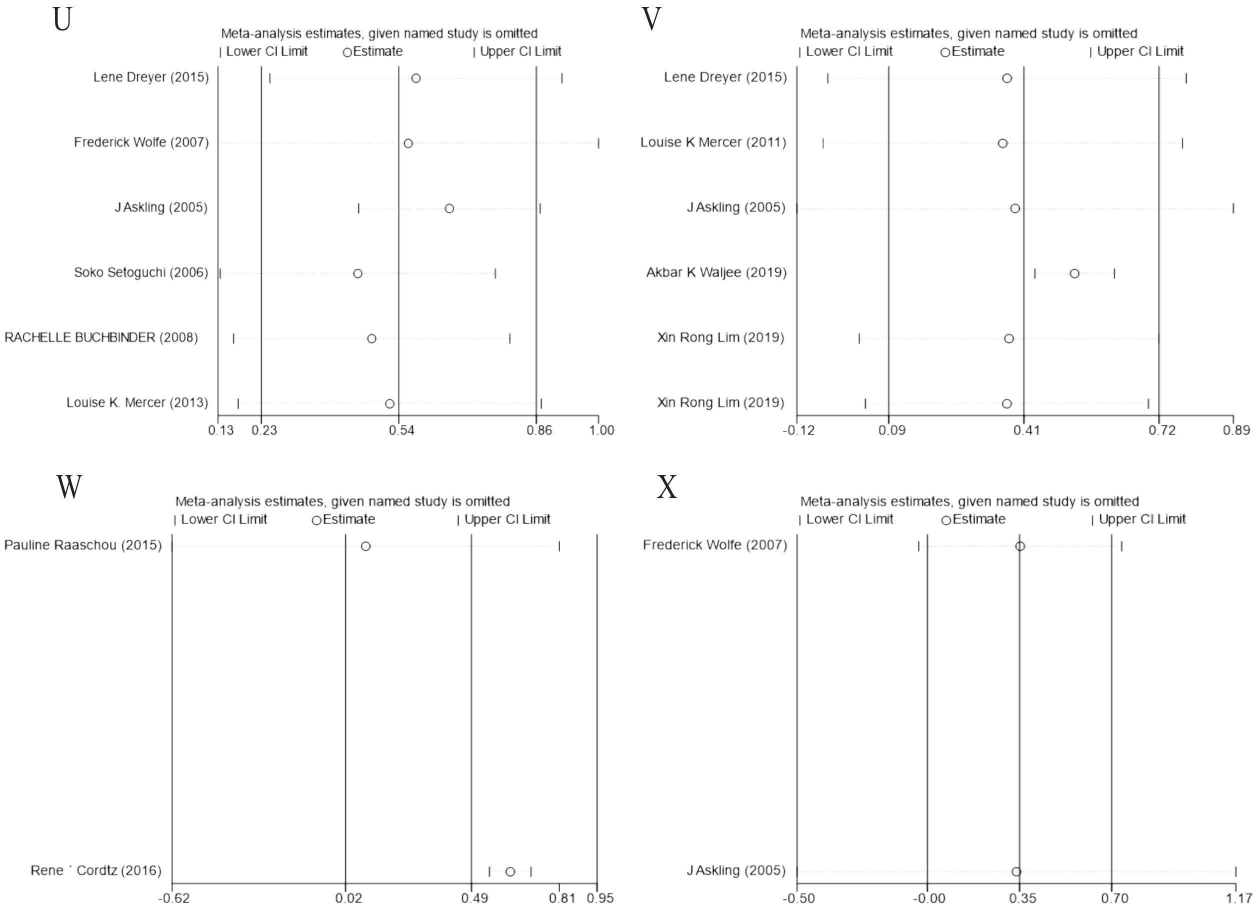
U
Meta-analysis estimates, given named study is omitted
Lower CI Limit
Estimate
Upper CI Limit
Lene Dreyer (2015)
Frederick Wolfe (2007)
J Askling (2005)
Soko Setoguchi (2006)
RACHELLE BUCHBINDER (2008)
Louise K. Mercer (2013)
0.13
0.23
0.54
0.86
1.00
V
Meta-analysis estimates, given named study is omitted
Lower CI Limit
Estimate
Upper CI Limit
Lene Dreyer (2015)
Louise K Mercer (2011)
J Askling (2005)
Akbar K Waljee (2019)
Xin Rong Lim (2019)
Xin Rong Lim (2019)
-0.12
0.09
0.41
0.72
0.89
W
Meta-analysis estimates, given named study is omitted
Lower CI Limit
Estimate
Upper CI Limit
Pauline Raaschou (2015)
Rene ´ Cordtz (2016)
-0.62
0.02
0.49
0.81
0.95
X
Meta-analysis estimates, given named study is omitted
Lower CI Limit
Estimate
Upper CI Limit
Frederick Wolfe (2007)
J Askling (2005)
-0.50
-0.00
0.35
0.70
1.17

Y

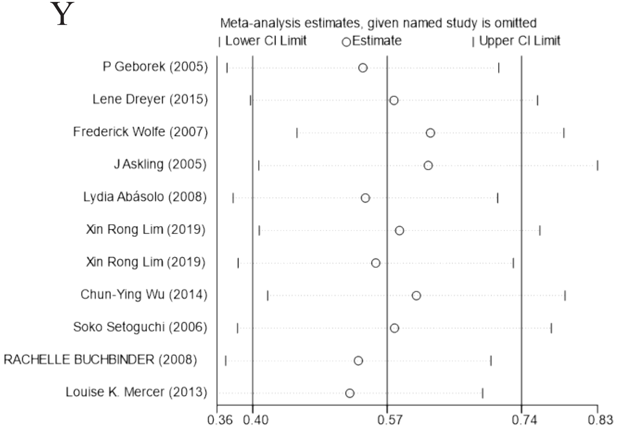

Supplementary Figure S2: Sensitivity analysis for cancers in RA patients with csDMARDs. Overall cancer (A), renal cancer (B), bladder cancer (C), cervical cancer (D), cancer of vulva and vagina (E), cancer of testis (F), prostate cancer (G), ovarian cancer (H), breast cancer (I), cancer of uterus corpus (J), leukemia (K), lymphoma (L), multiple myeloma (M), non Hodgkin's lymphoma (N), Hodgkin's lymphoma (O), colorectal cancer(P), pancreas cancer(Q), oesophageal cancer (R), liver cancer (S), gastric cancer (T), melanoma (U), non-melanoma skin cancer (V), squamous cell carcinoma (W), cancer of soft tissues (X), lung cancer (Y).

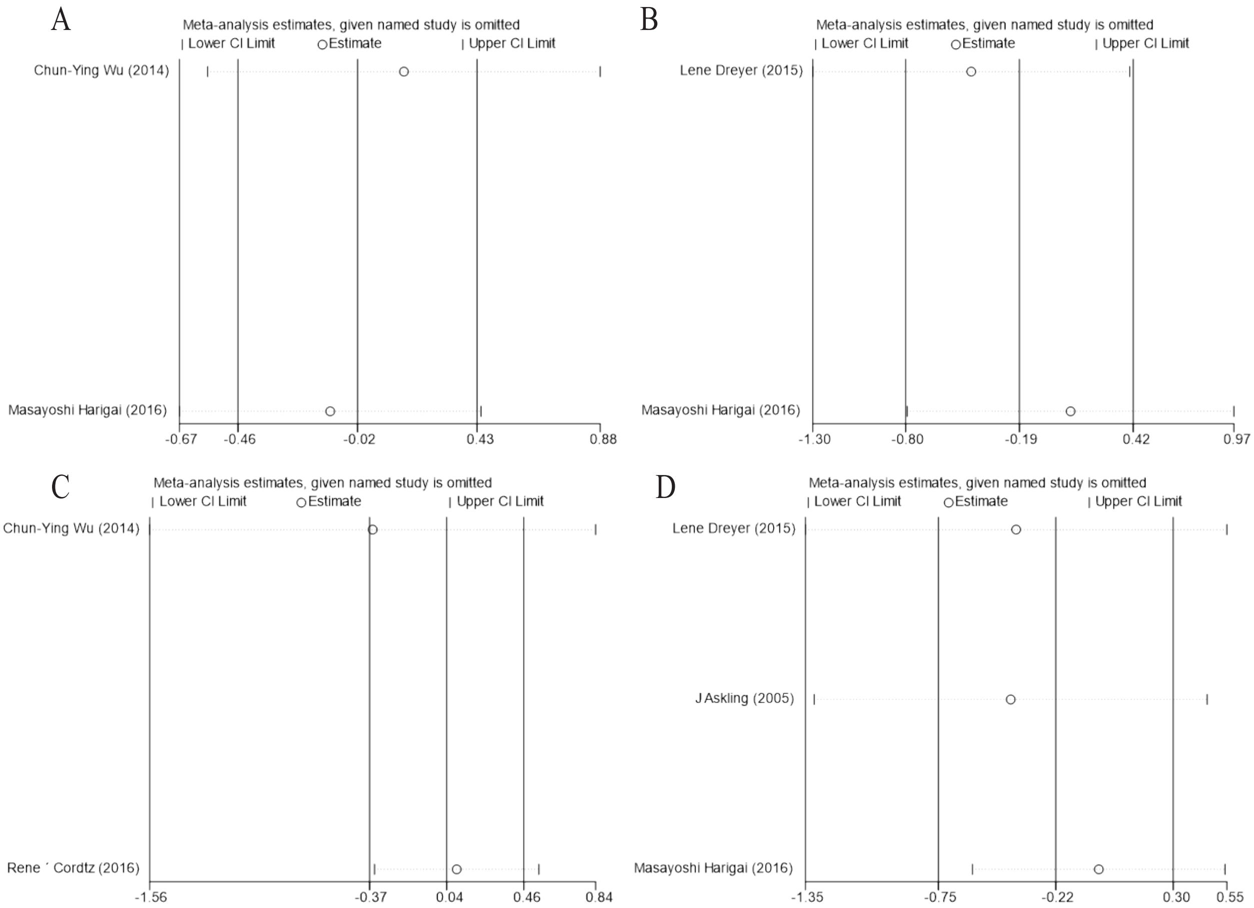
A
Meta-analysis estimates, given named study is omitted
Lower CI Limit
Estimate
Upper CI Limit
Chun-Ying Wu (2014)
Masayoshi Harigai (2016)
-0.67
-0.46
-0.02
0.43
0.88
B
Meta-analysis estimates, given named study is omitted
Lower CI Limit
Estimate
Upper CI Limit
Lene Dreyer (2015)
Masayoshi Harigai (2016)
-1.30
-0.80
-0.19
0.42
0.97
C
Meta-analysis estimates, given named study is omitted
Lower CI Limit
Estimate
Upper CI Limit
Chun-Ying Wu (2014)
Rene ´ Cordtz (2016)
-1.56
-0.37
0.04
0.46
0.84
D
Meta-analysis estimates, given named study is omitted
Lower CI Limit
Estimate
Upper CI Limit
Lene Dreyer (2015)
J Askling (2005)
Masayoshi Harigai (2016)
-1.35
-0.75
-0.22
0.30
0.55

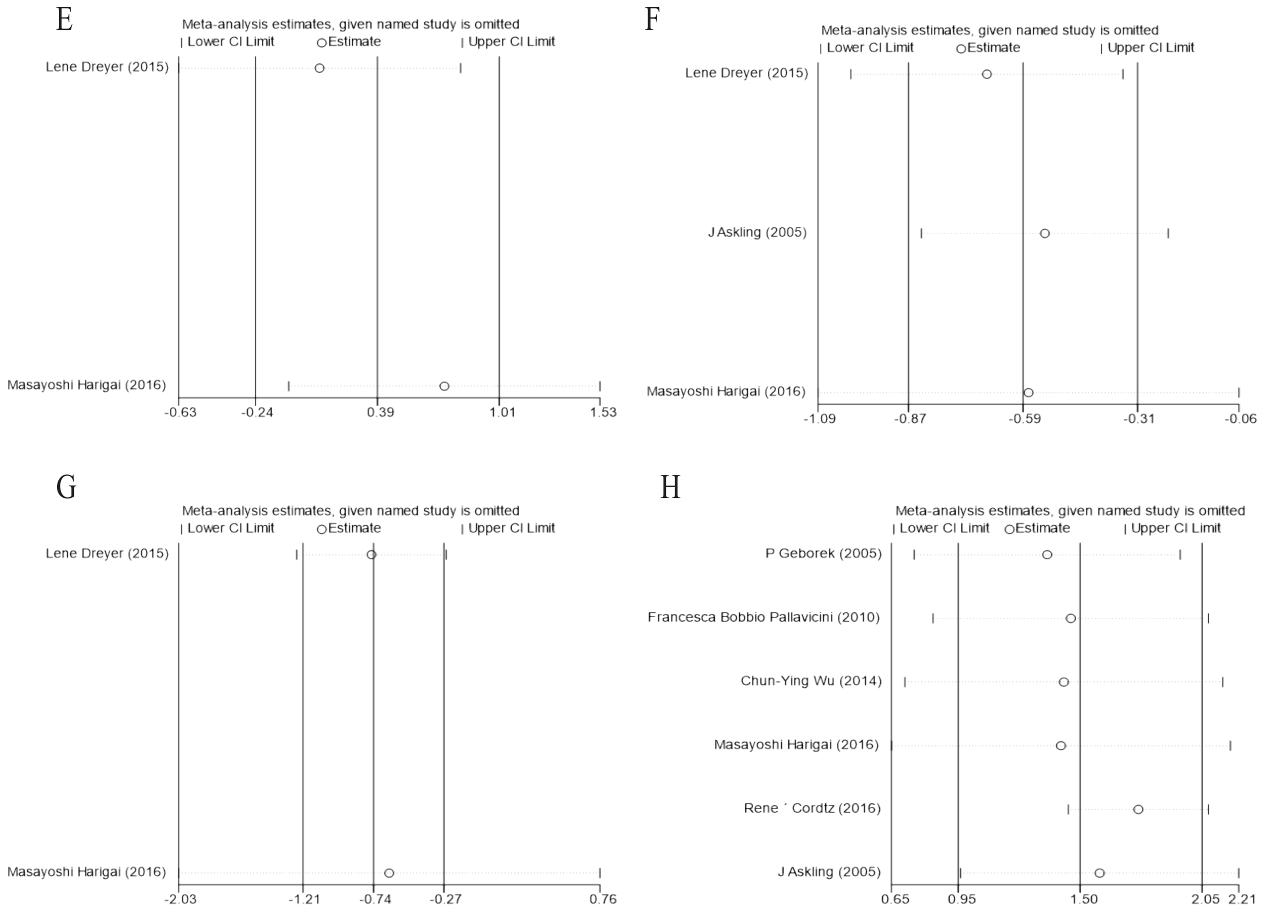
E
Meta-analysis estimates, given named study is omitted
Lower CI Limit
Estimate
Upper CI Limit
Lene Dreyer (2015)
Masayoshi Harigai (2016)
-0.63
-0.24
0.39
1.01
1.53
F
Meta-analysis estimates, given named study is omitted
Lower CI Limit
Estimate
Upper CI Limit
Lene Dreyer (2015)
J Askling (2005)
Masayoshi Harigai (2016)
-1.09
-0.87
-0.59
-0.31
-0.06
G
Meta-analysis estimates, given named study is omitted
Lower CI Limit
Estimate
Upper CI Limit
Lene Dreyer (2015)
Masayoshi Harigai (2016)
-2.03
-1.21
-0.74
-0.27
0.76
H
Meta-analysis estimates, given named study is omitted
Lower CI Limit
Estimate
Upper CI Limit
P Geborek (2005)
Francesca Bobbio Pallavicini (2010)
Chun-Ying Wu (2014)
Masayoshi Harigai (2016)
Rene ´ Cordtz (2016)
J Askling (2005)
0.65
0.95
1.50
2.05
2.21

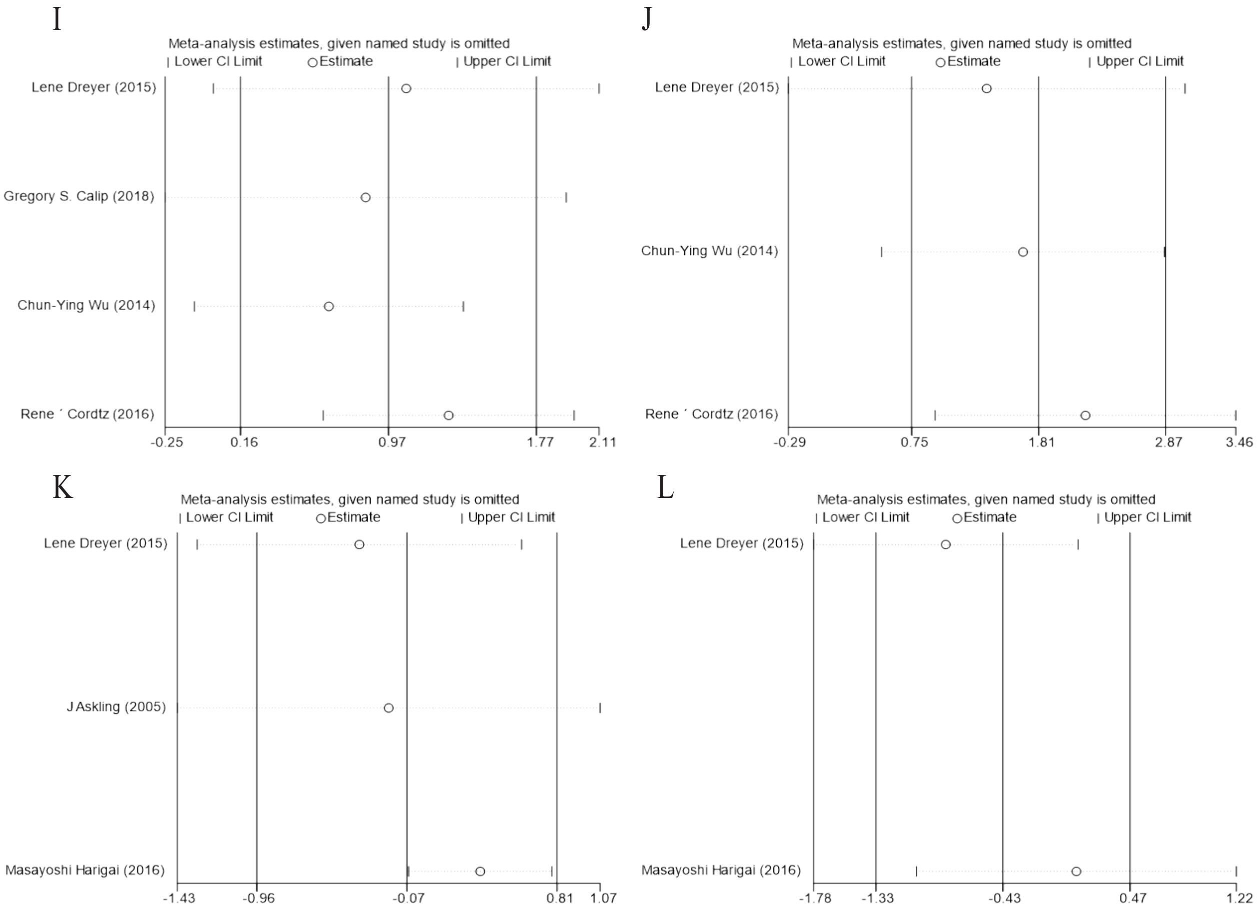
I
Meta-analysis estimates, given named study is omitted
Lower CI Limit
Estimate
Upper CI Limit
Lene Dreyer (2015)
Gregory S. Calip (2018)
Chun-Ying Wu (2014)
Rene ´ Cordtz (2016)
-0.25
0.16
0.97
1.77
2.11
J
Meta-analysis estimates, given named study is omitted
Lower CI Limit
Estimate
Upper CI Limit
Lene Dreyer (2015)
Chun-Ying Wu (2014)
Rene ´ Cordtz (2016)
-0.29
0.75
1.81
2.87
3.46
K
Meta-analysis estimates, given named study is omitted
Lower CI Limit
Estimate
Upper CI Limit
Lene Dreyer (2015)
J Askling (2005)
Masayoshi Harigai (2016)
-1.43
-0.96
-0.07
0.81
1.07
L
Meta-analysis estimates, given named study is omitted
Lower CI Limit
Estimate
Upper CI Limit
Lene Dreyer (2015)
Masayoshi Harigai (2016)
-1.78
-1.33
-0.43
0.47
1.22

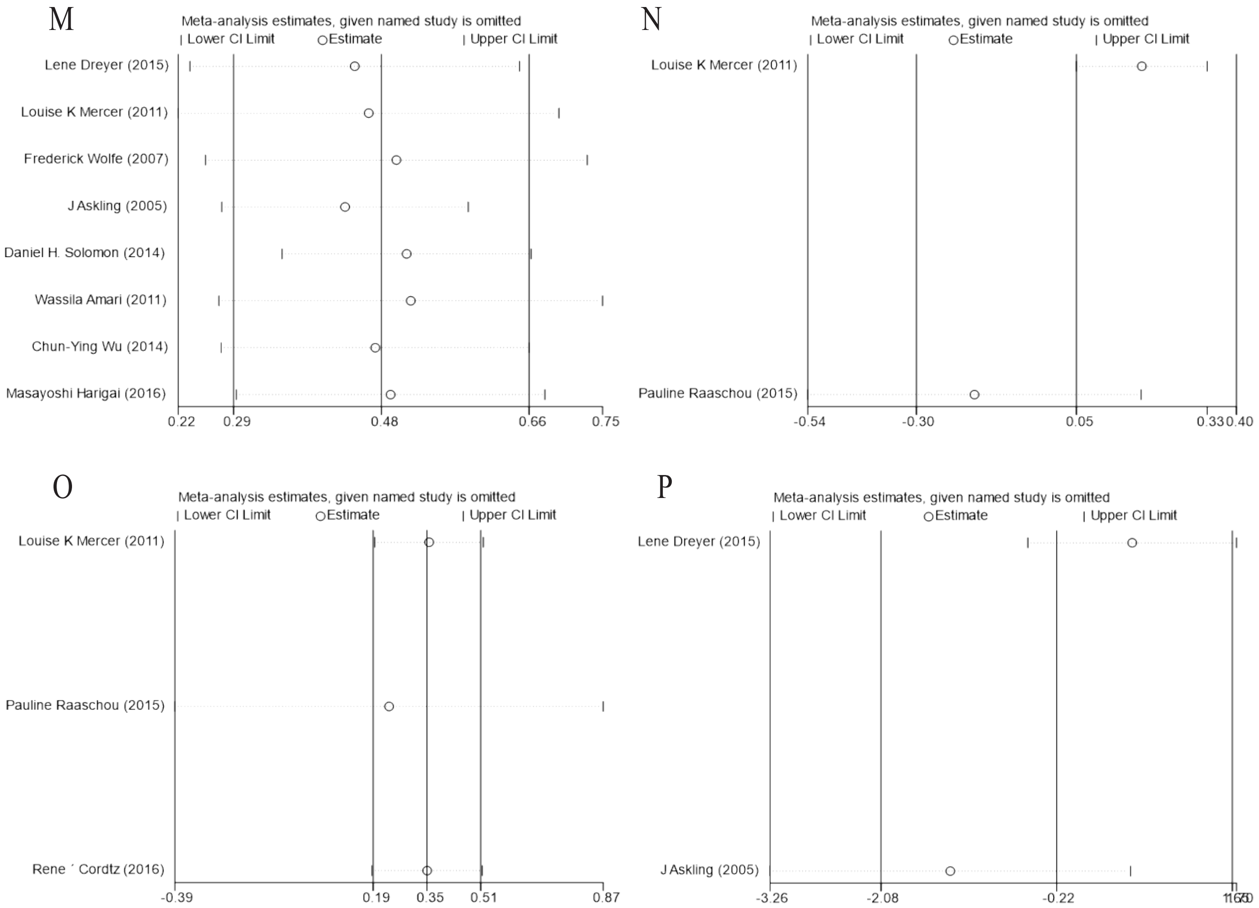
M
Meta-analysis estimates, given named study is omitted
Lower CI Limit
Estimate
Upper CI Limit
Lene Dreyer (2015)
Louise K Mercer (2011)
Frederick Wolfe (2007)
J Askling (2005)
Daniel H. Solomon (2014)
Wassila Amari (2011)
Chun-Ying Wu (2014)
Masayoshi Harigai (2016)
0.22
0.29
0.48
0.66
0.75
N
Meta-analysis estimates, given named study is omitted
Lower CI Limit
Estimate
Upper CI Limit
Louise K Mercer (2011)
Pauline Raaschou (2015)
-0.54
-0.30
0.05
0.33
0.40
O
Meta-analysis estimates, given named study is omitted
Lower CI Limit
Estimate
Upper CI Limit
Louise K Mercer (2011)
Pauline Raaschou (2015)
Rene ´ Cordtz (2016)
-0.39
0.19
0.35
0.51
0.87
P
Meta-analysis estimates, given named study is omitted
Lower CI Limit
Estimate
Upper CI Limit
Lene Dreyer (2015)
J Askling (2005)
-3.26
-2.08
-0.22

Q

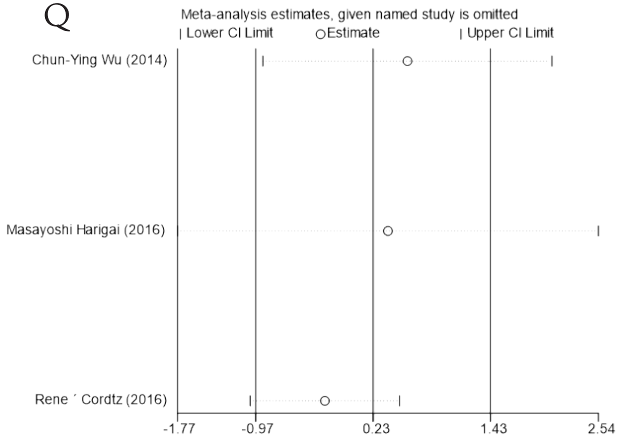

R

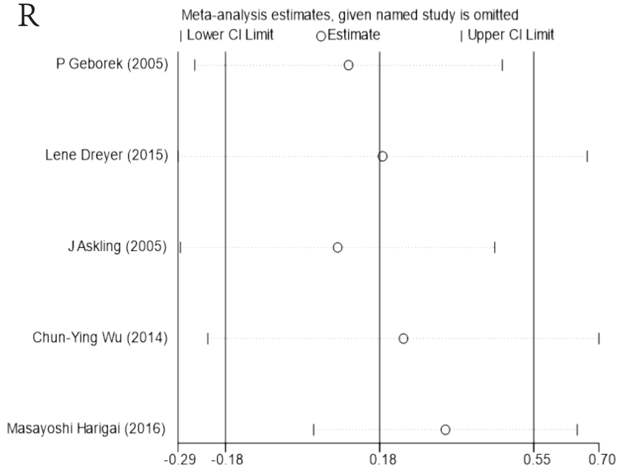

Supplementary Figure S3: Sensitivity analysis for cancers in RA patients with bDMARDs. renal cancer (A), bladder cancer (B), cervical cancer (C), prostate cancer (D), ovarian cancer (E), breast cancer (F), cancer of uterus corpus (G), lymphoma (H), non-Hodgkin's lymphoma (I), Hodgkin's lymphoma (J), colorectal cancer(K), pancreas cancer(L), non-melanoma skin cancer (M), basal cell carcinoma (N), squamous cell carcinoma (O), brain and CNS cancer (P), oral cancer (Q), lung cancer (R).

Supplementary Figure S4. Overall cancer risk stratified by region. csDMARDs(A), bDMARDs(B).

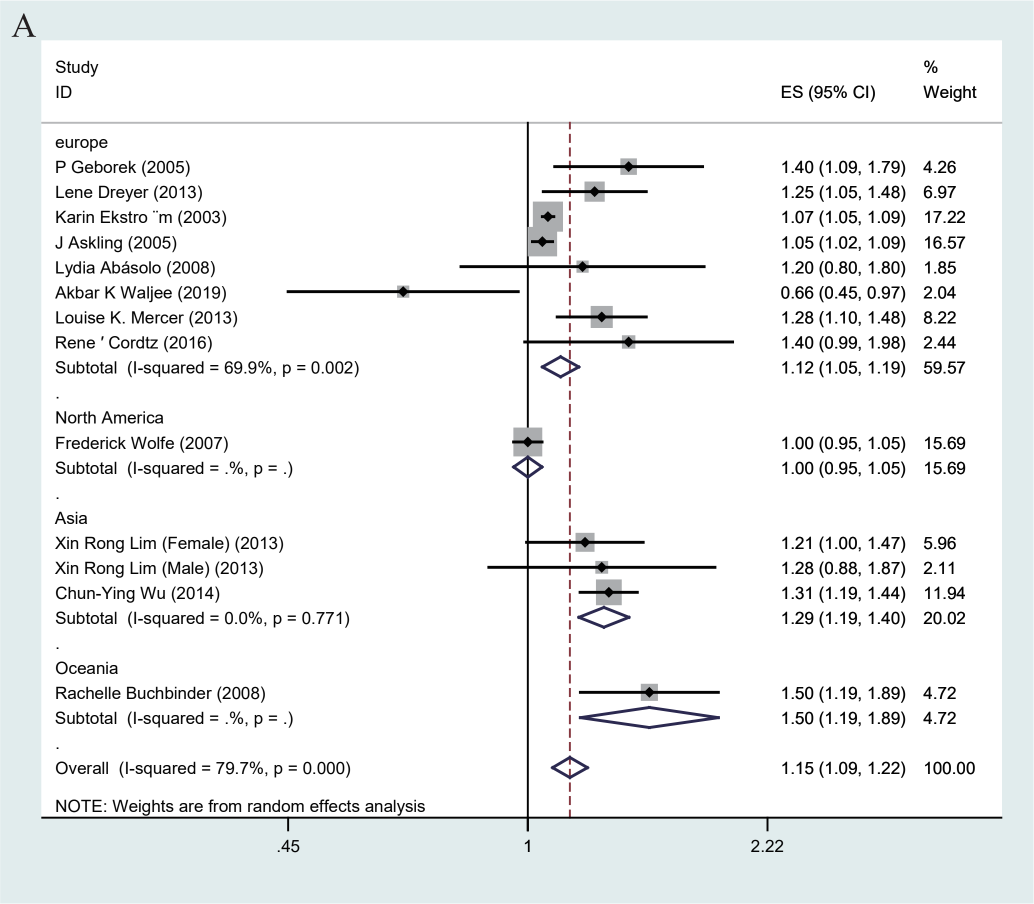

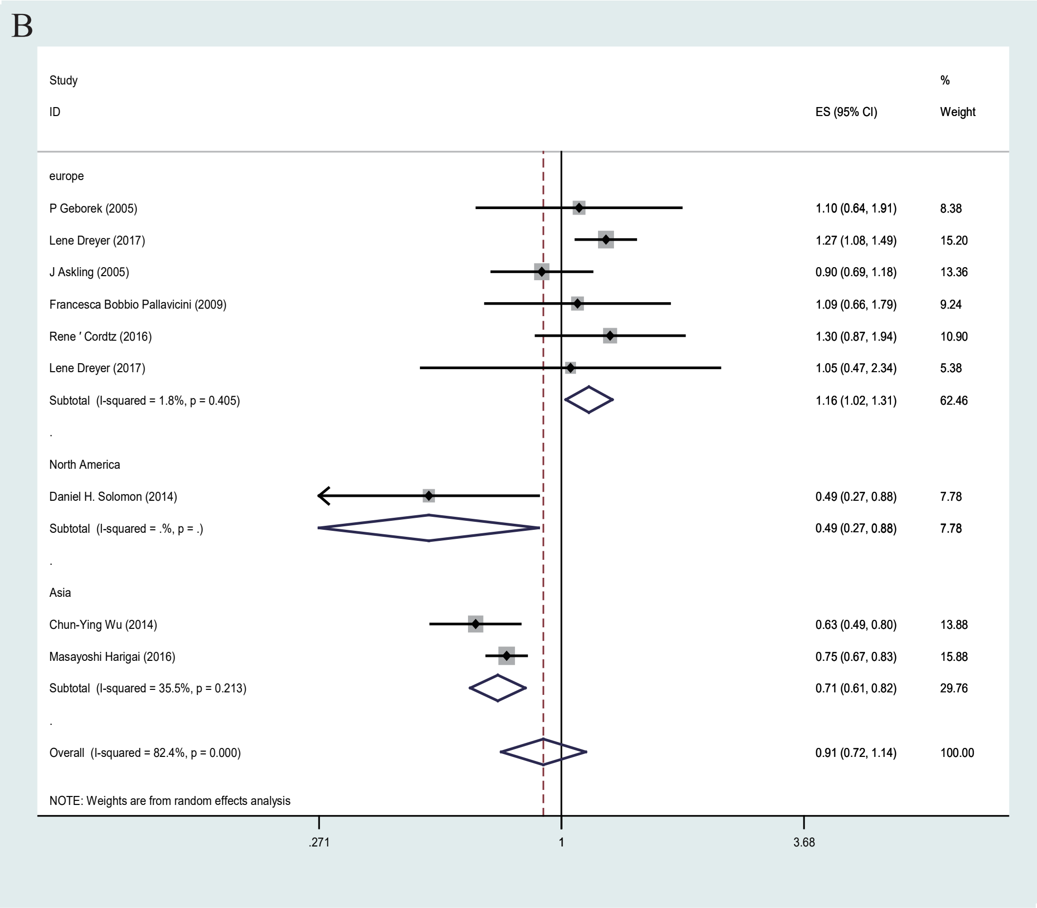
B
Study
ID
ES (95% CI)
%
Weight
europe
P Geborek (2005) 1.10 (0.64, 1.91) 8.38
Lene Dreyer (2017) 1.27 (1.08, 1.49) 15.20
J Askling (2005) 0.90 (0.69, 1.18) 13.36
Francesca Bobbio Pallavicini (2009) 1.09 (0.66, 1.79) 9.24
Rene ′ Cordtz (2016) 1.30 (0.87, 1.94) 10.90
Lene Dreyer (2017) 1.05 (0.47, 2.34) 5.38
Subtotal (I-squared = 1.8%, p = 0.405) 1.16 (1.02, 1.31) 62.46
.
North America
Daniel H. Solomon (2014) 0.49 (0.27, 0.88) 7.78
Subtotal (I-squared = .%, p = .) 0.49 (0.27, 0.88) 7.78
.
Asia
Chun-Ying Wu (2014) 0.63 (0.49, 0.80) 13.88
Masayoshi Harigai (2016) 0.75 (0.67, 0.83) 15.88
Subtotal (I-squared = 35.5%, p = 0.213) 0.71 (0.61, 0.82) 29.76
.
Overall (I-squared = 82.4%, p = 0.000) 0.91 (0.72, 1.14) 100.00
NOTE: Weights are from random effects analysis
.271
1
3.68

Supplementary Figure S4. Overall cancer risk stratified by age. csDMARDS, 55 year old as cut-off point(C), csDMARDS, 60 year old as cut-off point(D), bDMARDS, 55 year old as cut-off point(E).

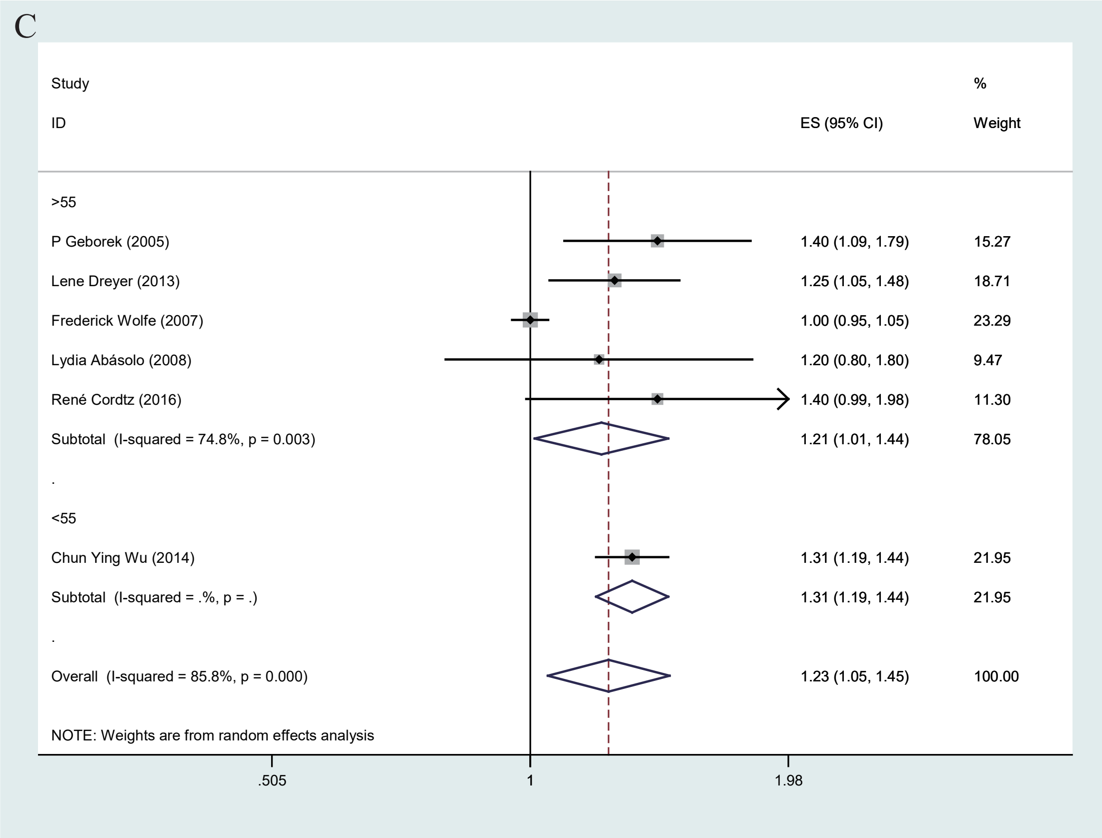

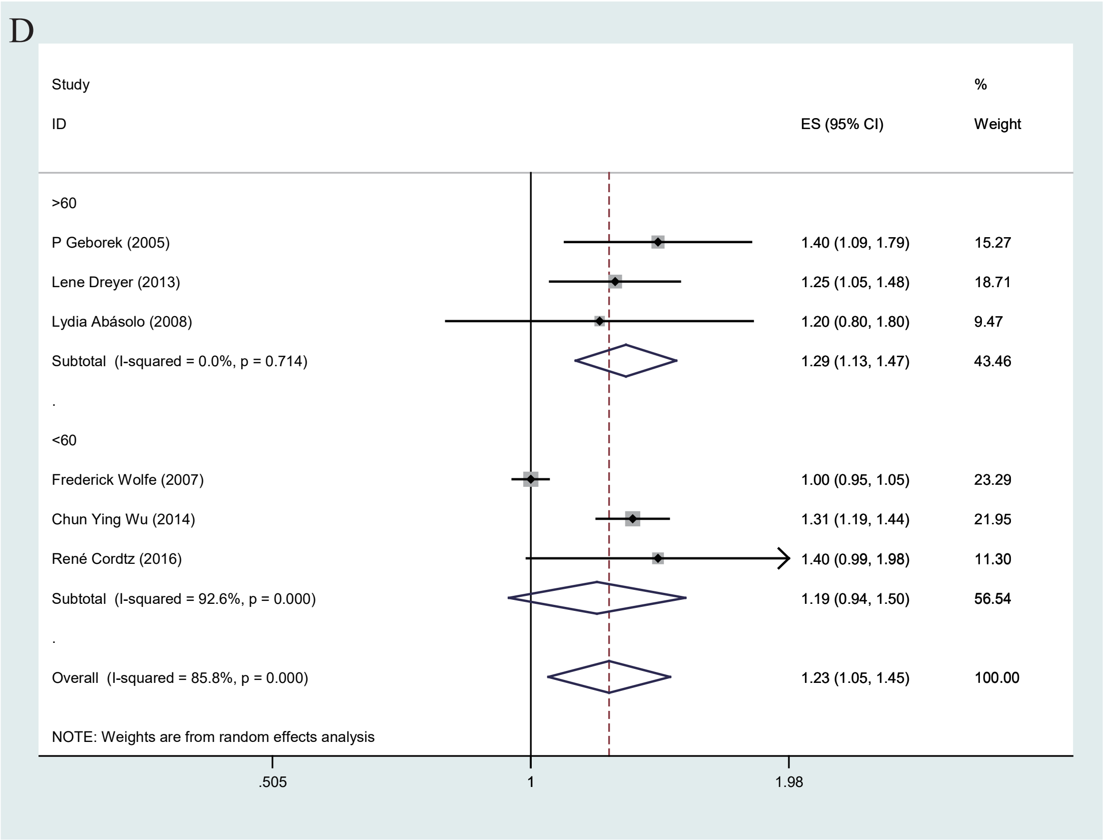
D
Study
ID
ES (95% CI)
%
Weight
>60
P Geborek (2005) 1.40 (1.09, 1.79) 15.27
Lene Dreyer (2013) 1.25 (1.05, 1.48) 18.71
Lydia Abásolo (2008) 1.20 (0.80, 1.80) 9.47
Subtotal (I-squared = 0.0%, p = 0.714) 1.29 (1.13, 1.47) 43.46
.
<60
Frederick Wolfe (2007) 1.00 (0.95, 1.05) 23.29
Chun Ying Wu (2014) 1.31 (1.19, 1.44) 21.95
René Cordtz (2016) 1.40 (0.99, 1.98) 11.30
Subtotal (I-squared = 92.6%, p = 0.000) 1.19 (0.94, 1.50) 56.54
.
Overall (I-squared = 85.8%, p = 0.000) 1.23 (1.05, 1.45) 100.00
NOTE: Weights are from random effects analysis
.505
1
1.98

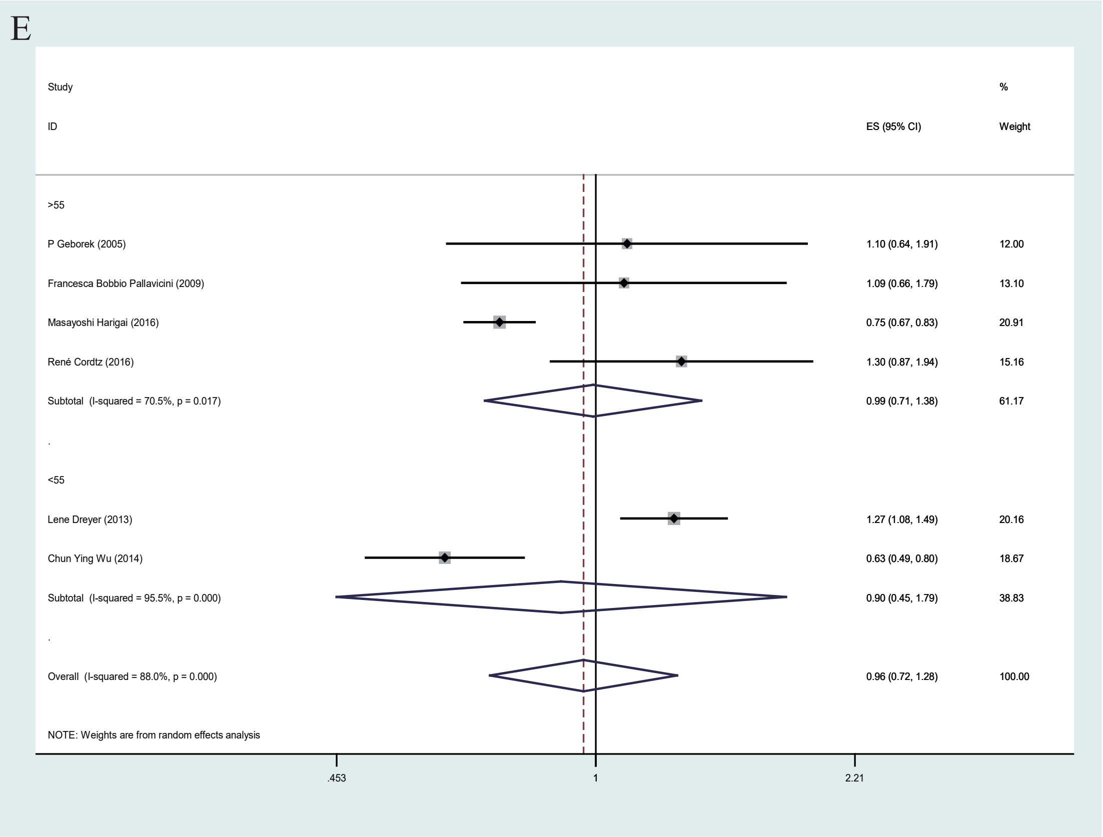
E
Study
ID
ES (95% CI)
%
Weight
>55
P Geborek (2005)
1.10 (0.64, 1.91)
12.00
Francesca Bobbio Pallavicini (2009)
1.09 (0.66, 1.79)
13.10
Masayoshi Harigai (2016)
0.75 (0.67, 0.83)
20.91
René Cordtz (2016)
1.30 (0.87, 1.94)
15.16
Subtotal (I-squared = 70.5%, p = 0.017)
0.99 (0.71, 1.38)
61.17
.
<55
Lene Dreyer (2013)
1.27 (1.08, 1.49)
20.16
Chun Ying Wu (2014)
0.63 (0.49, 0.80)
18.67
Subtotal (I-squared = 95.5%, p = 0.000)
0.90 (0.45, 1.79)
38.83
.
Overall (I-squared = 88.0%, p = 0.000)
0.96 (0.72, 1.28)
100.00
NOTE: Weights are from random effects analysis
.453
1
2.21

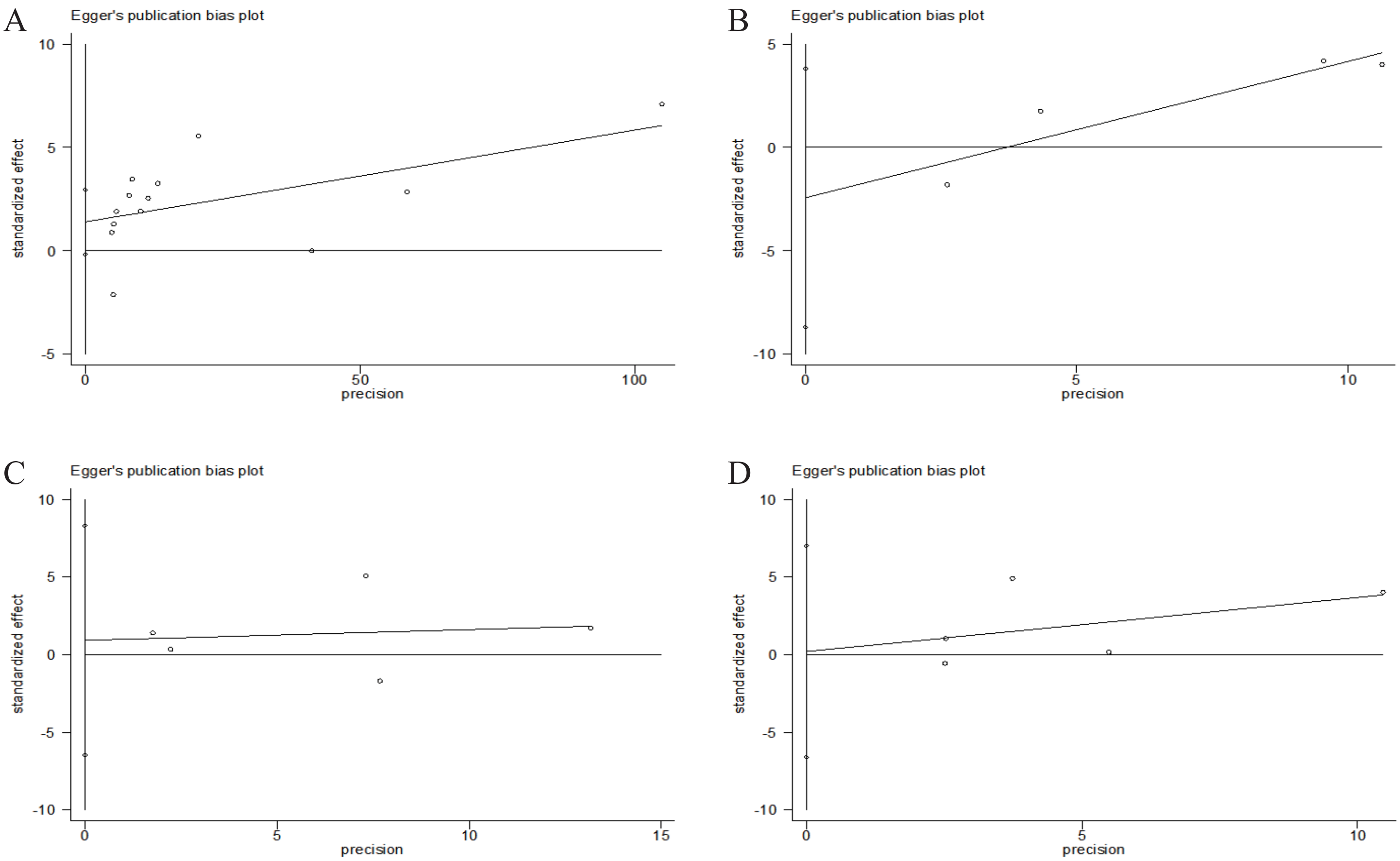
A
Egger's publication bias plot
standardized effect
precision
10
5
0
-5
0
50
100
B
Egger's publication bias plot
standardized effect
precision
5
0
-5
-10
0
5
10
C
Egger's publication bias plot
standardized effect
precision
10
5
0
-5
-10
0
5
10
15
D
Egger's publication bias plot
standardized effect
precision
10
5
0
-5
-10
0
5
10

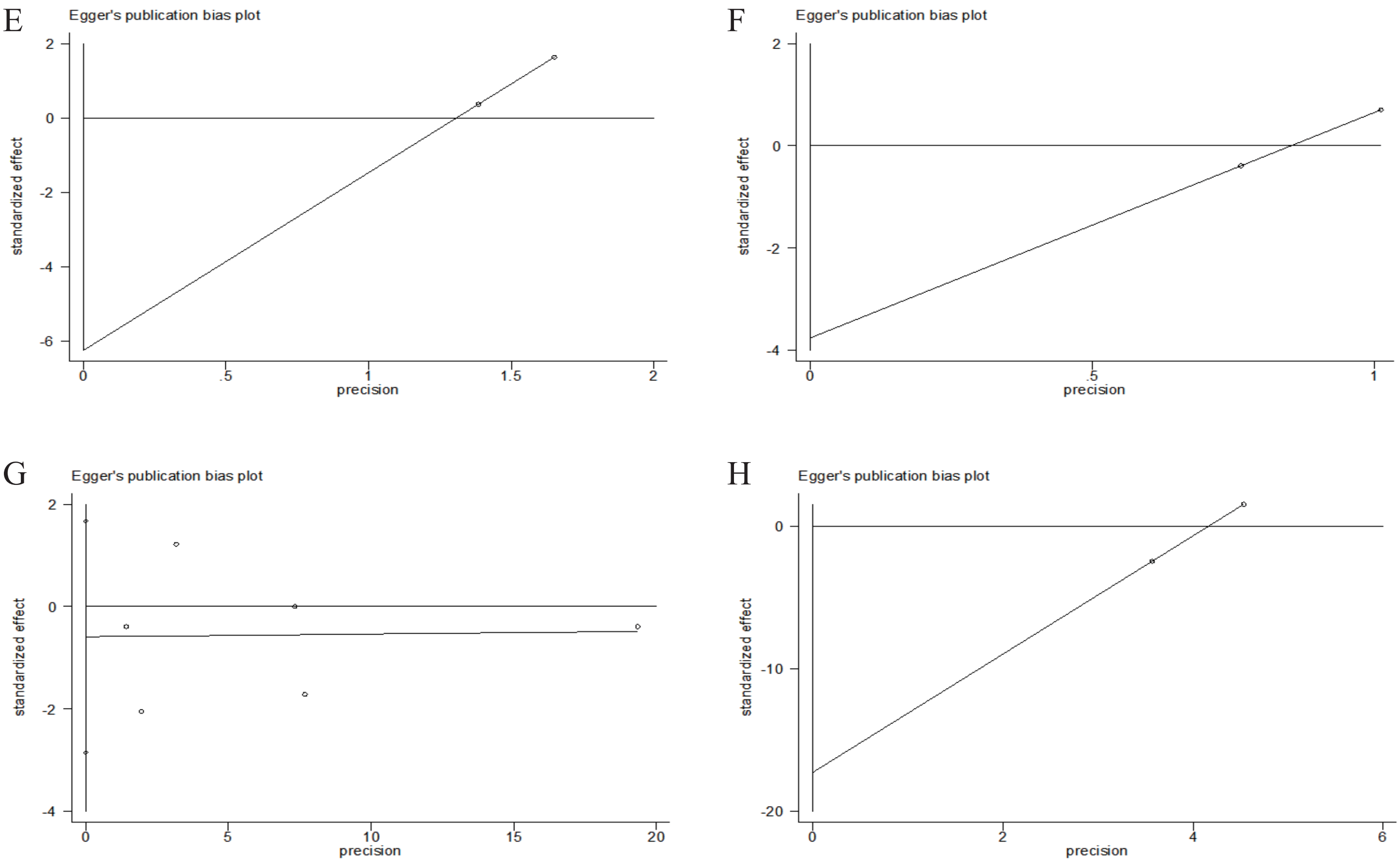
E
Egger's publication bias plot
standardized effect
precision
2
0
-2
-4
-6
0
.5
1
1.5
2
F
Egger's publication bias plot
standardized effect
precision
2
0
-2
-4
0
.5
1
G
Egger's publication bias plot
standardized effect
precision
2
0
-2
-4
0
5
10
15
20
H
Egger's publication bias plot
standardized effect
precision
0
-10
-20
0
2
4
6

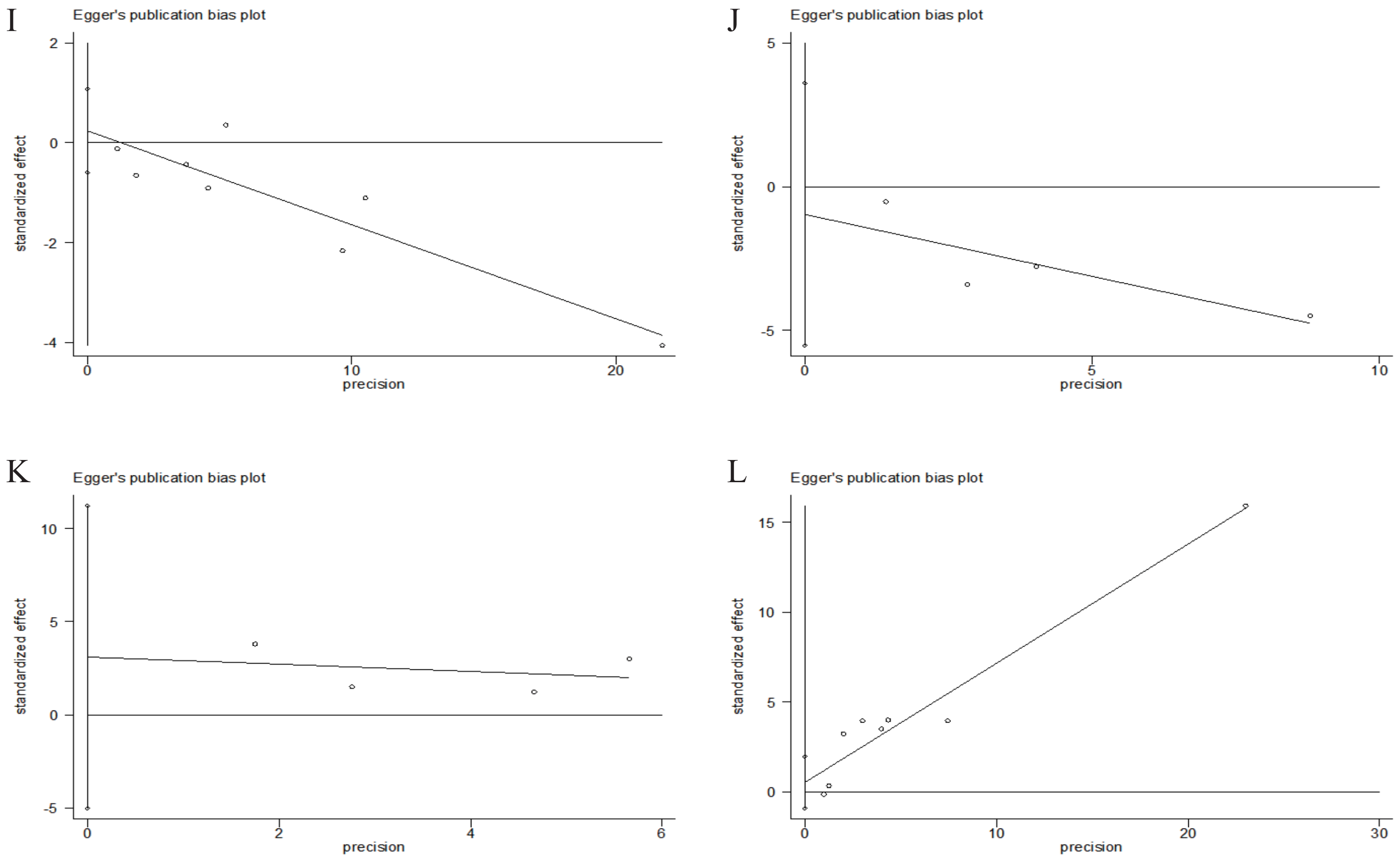
I
Egger's publication bias plot
standardized effect
precision
J
Egger's publication bias plot
standardized effect
precision
K
Egger's publication bias plot
standardized effect
precision
L
Egger's publication bias plot
standardized effect
precision

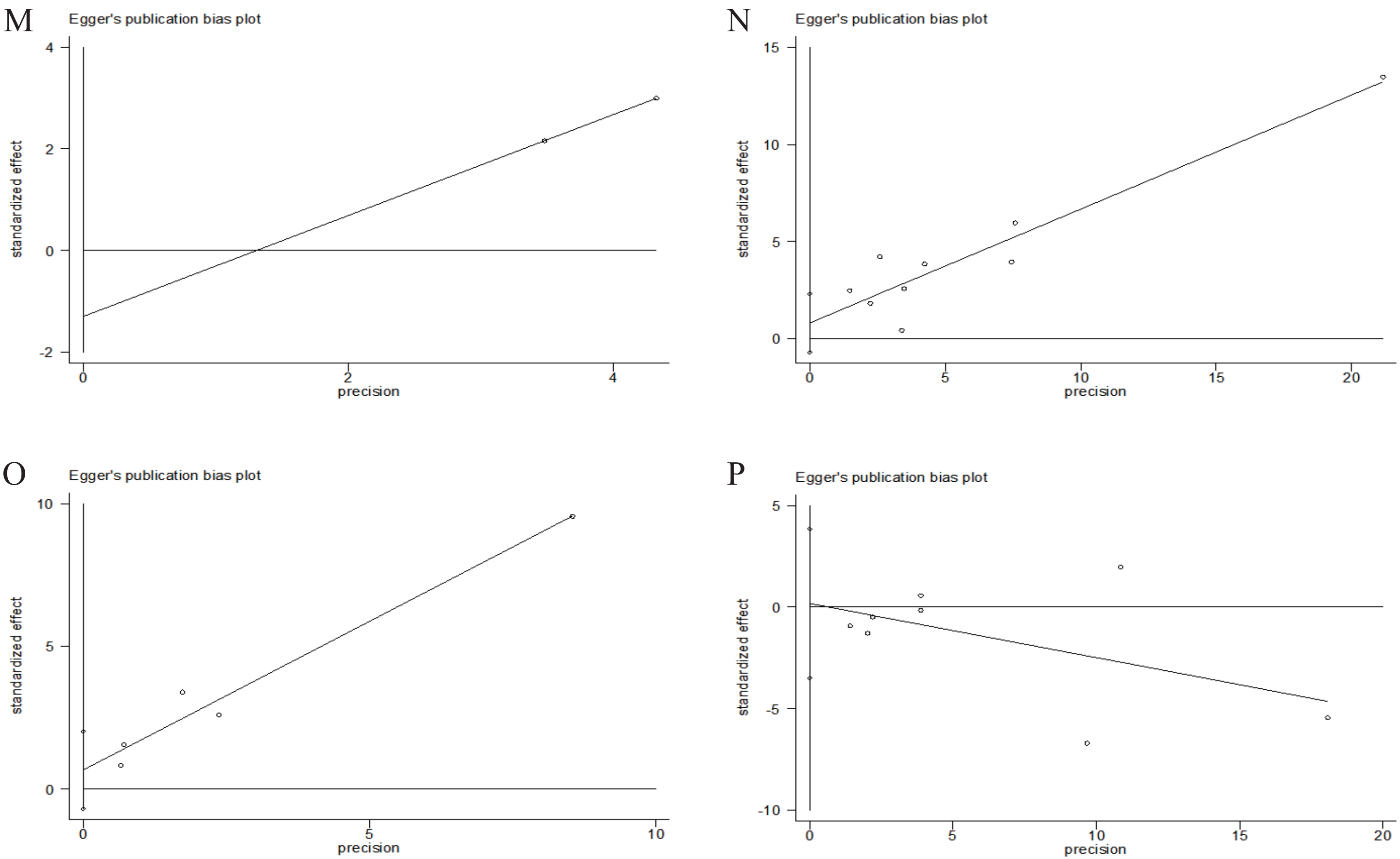
M
Egger's publication bias plot
standardized effect
precision
N
Egger's publication bias plot
standardized effect
precision
O
Egger's publication bias plot
standardized effect
precision
P
Egger's publication bias plot
standardized effect
precision

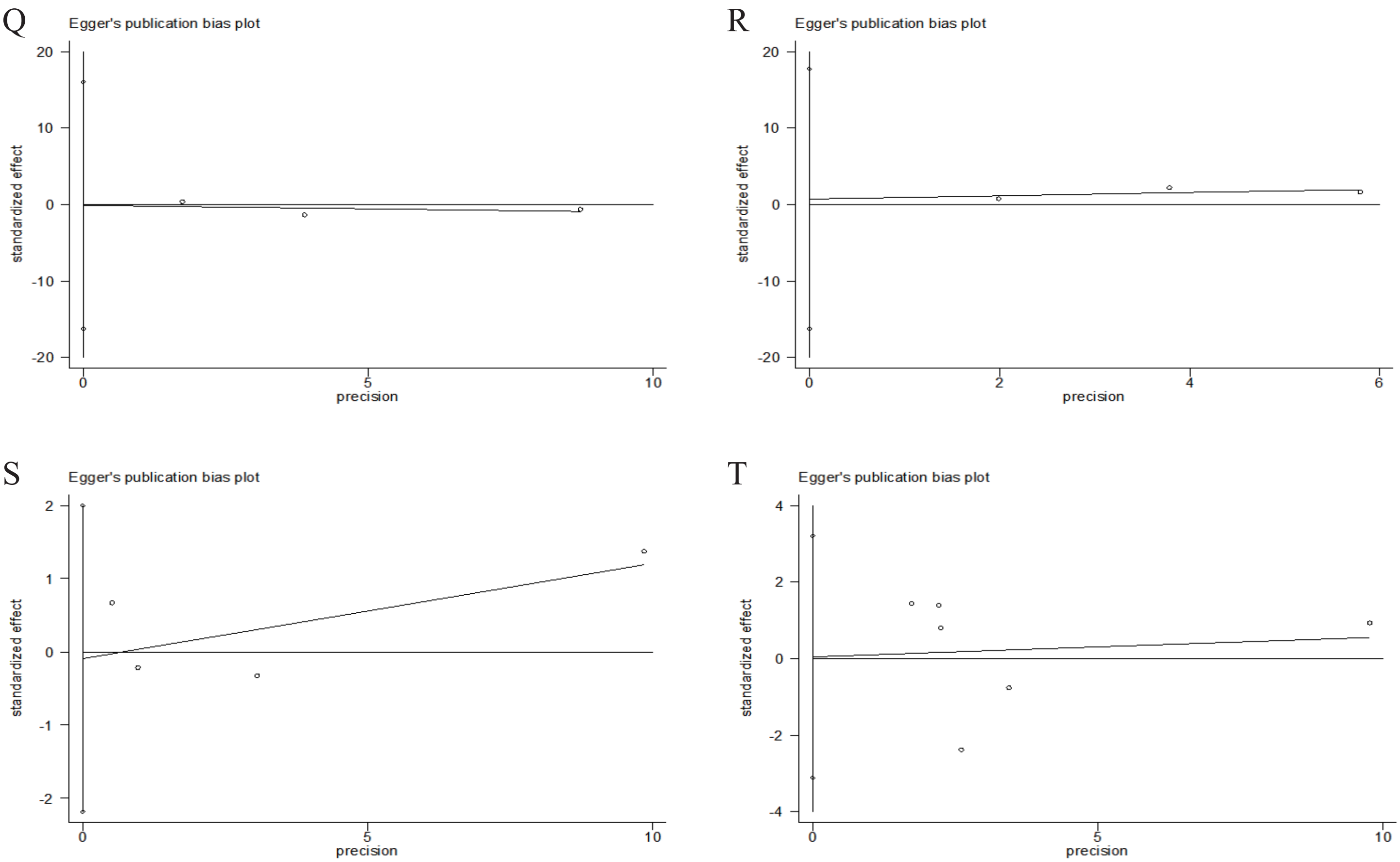
Q
Egger's publication bias plot
standardized effect
20
10
0
-10
-20
0
5
10
precision
R
Egger's publication bias plot
standardized effect
20
10
0
-10
-20
0
2
4
6
precision
S
Egger's publication bias plot
standardized effect
2
1
0
-1
-2
0
5
10
precision
T
Egger's publication bias plot
standardized effect
4
2
0
-2
-4
0
5
10
precision

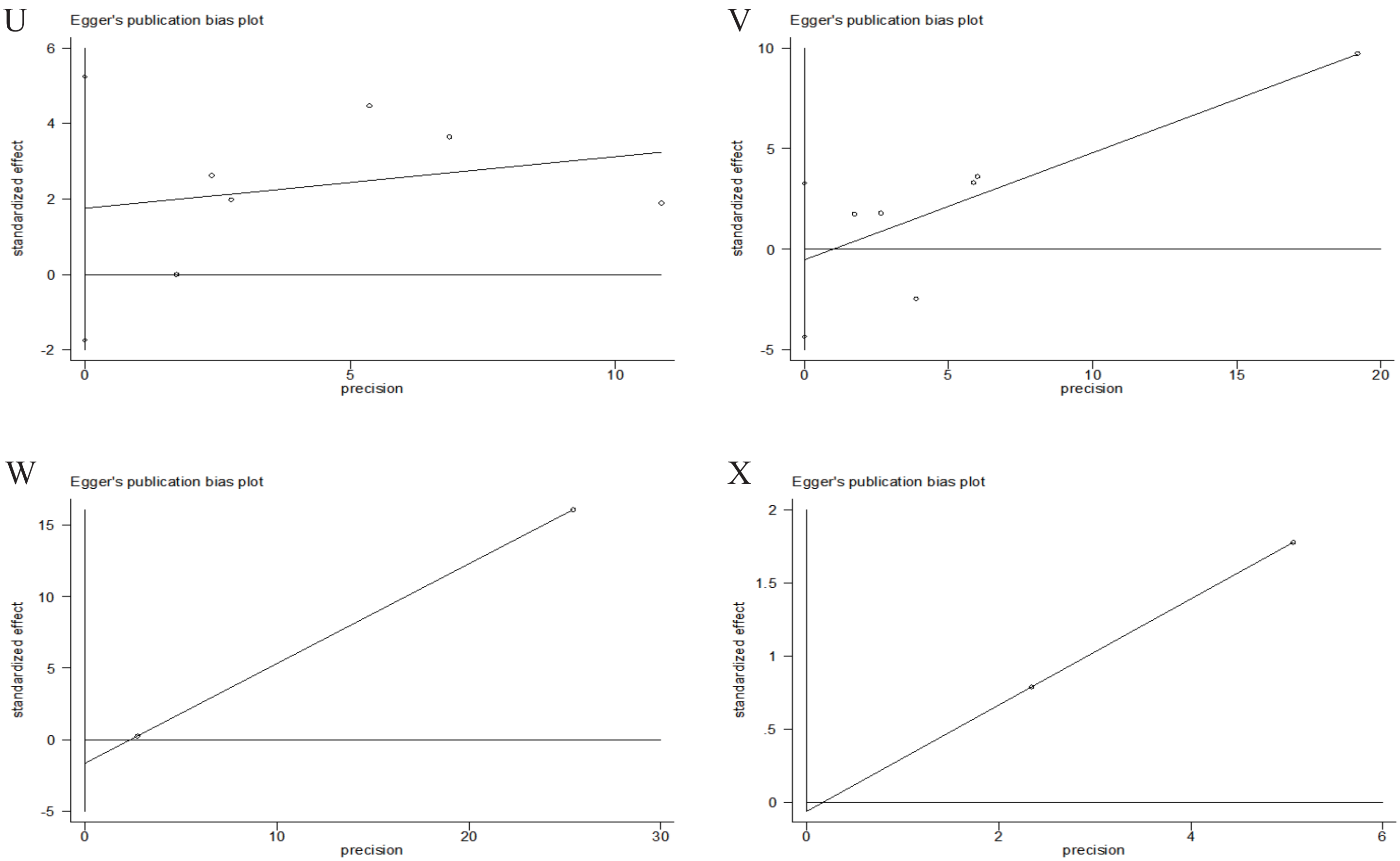
U
Egger's publication bias plot
standardized effect
precision
6
4
2
0
-2
0
5
10
V
Egger's publication bias plot
standardized effect
precision
10
5
0
-5
0
5
10
15
20
W
Egger's publication bias plot
standardized effect
precision
15
10
5
0
-5
0
10
20
30
X
Egger's publication bias plot
standardized effect
precision
2
1.5
1
.5
0
0
2
4
6

Y

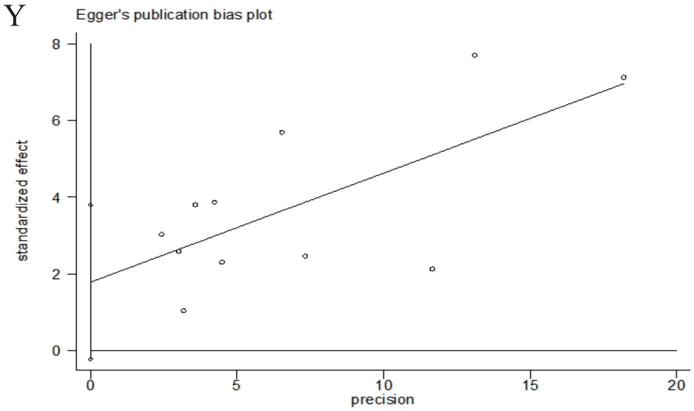

Supplementary Figure S5: Sensitivity analysis for cancers in RA patients with csDMARDs. Overall cancer (A), renal cancer (B), bladder cancer (C), cervical cancer (D), cancer of vulva and vagina (E), cancer of testis (F), prostate cancer (G), ovarian cancer (H), breast cancer (I), cancer of uterus corpus (J), leukemia (K), lymphoma (L), multiple myeloma (M), non Hodgkin's lymphoma (N), Hodgkin's lymphoma (O), colorectal cancer(P), pancreas cancer(Q),oesophageal cancer (R), liver cancer (S), gastric cancer (T), melanoma (U), non-melanoma skin cancer (V), squamous cell carcinoma (W), cancer of soft tissues (X), lung cancer (Y).

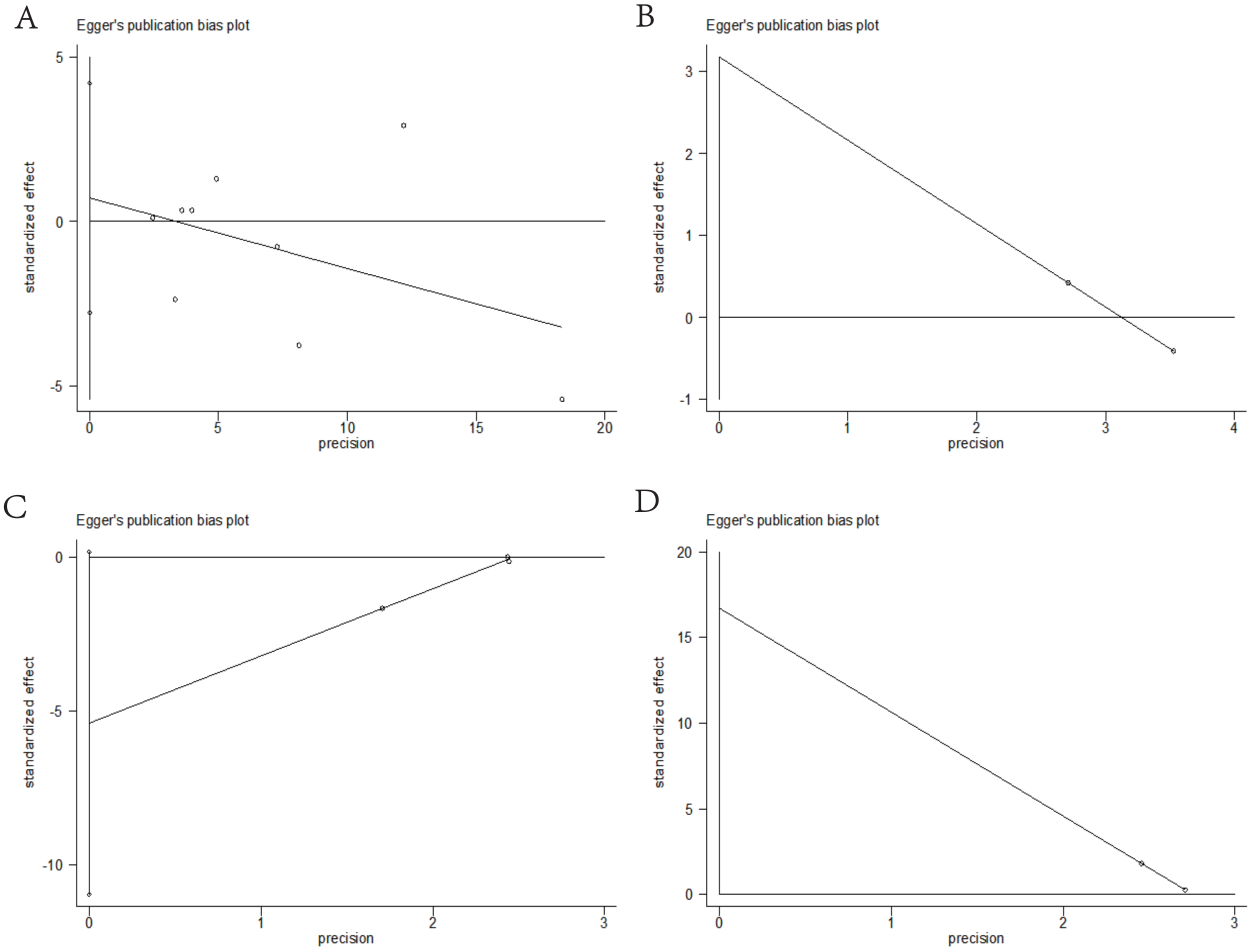
A
Egger's publication bias plot
standardized effect
precision
5
0
-5
0
5
10
15
20
B
Egger's publication bias plot
standardized effect
precision
3
2
1
0
-1
0
1
2
3
4
C
Egger's publication bias plot
standardized effect
precision
0
-5
-10
0
1
2
3
D
Egger's publication bias plot
standardized effect
precision
20
15
10
5
0
0
1
2
3

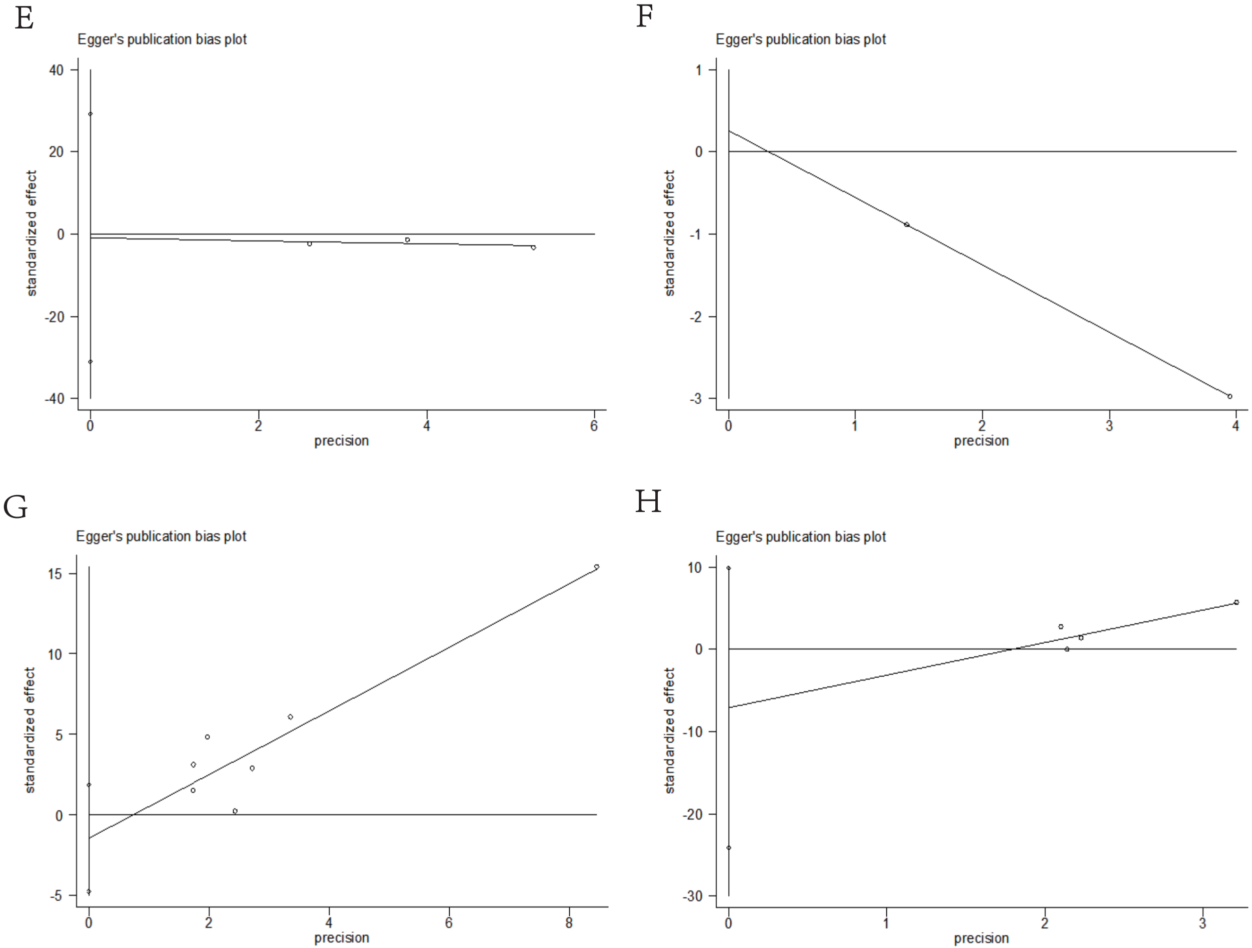

E
Egger's publication bias plot
standardized effect
precision
F
Egger's publication bias plot
standardized effect
precision
G
Egger's publication bias plot
standardized effect
precision
H
Egger's publication bias plot
standardized effect
precision

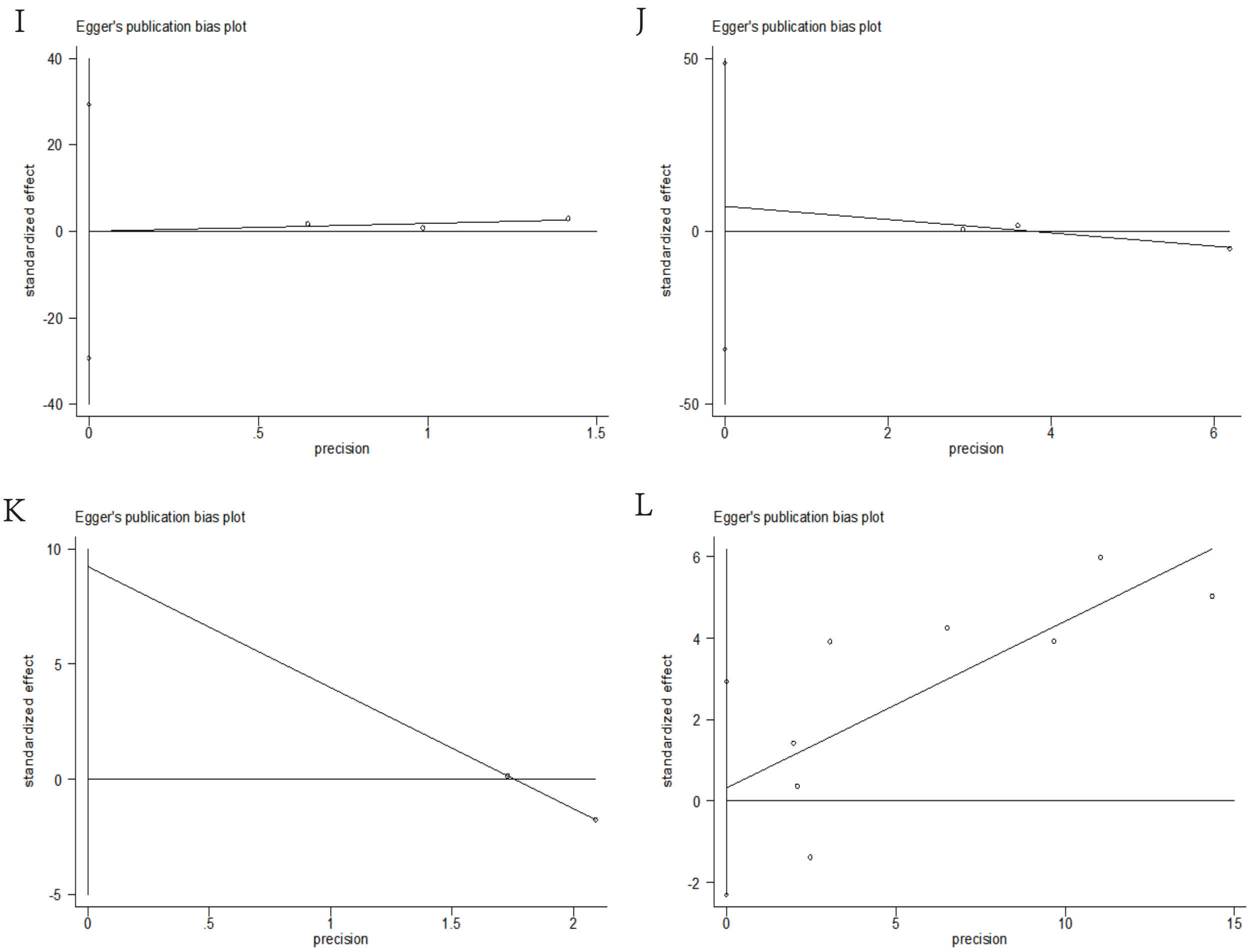

I
Egger's publication bias plot
standardized effect
precision
J
Egger's publication bias plot
standardized effect
precision
K
Egger's publication bias plot
standardized effect
precision
L
Egger's publication bias plot
standardized effect
precision

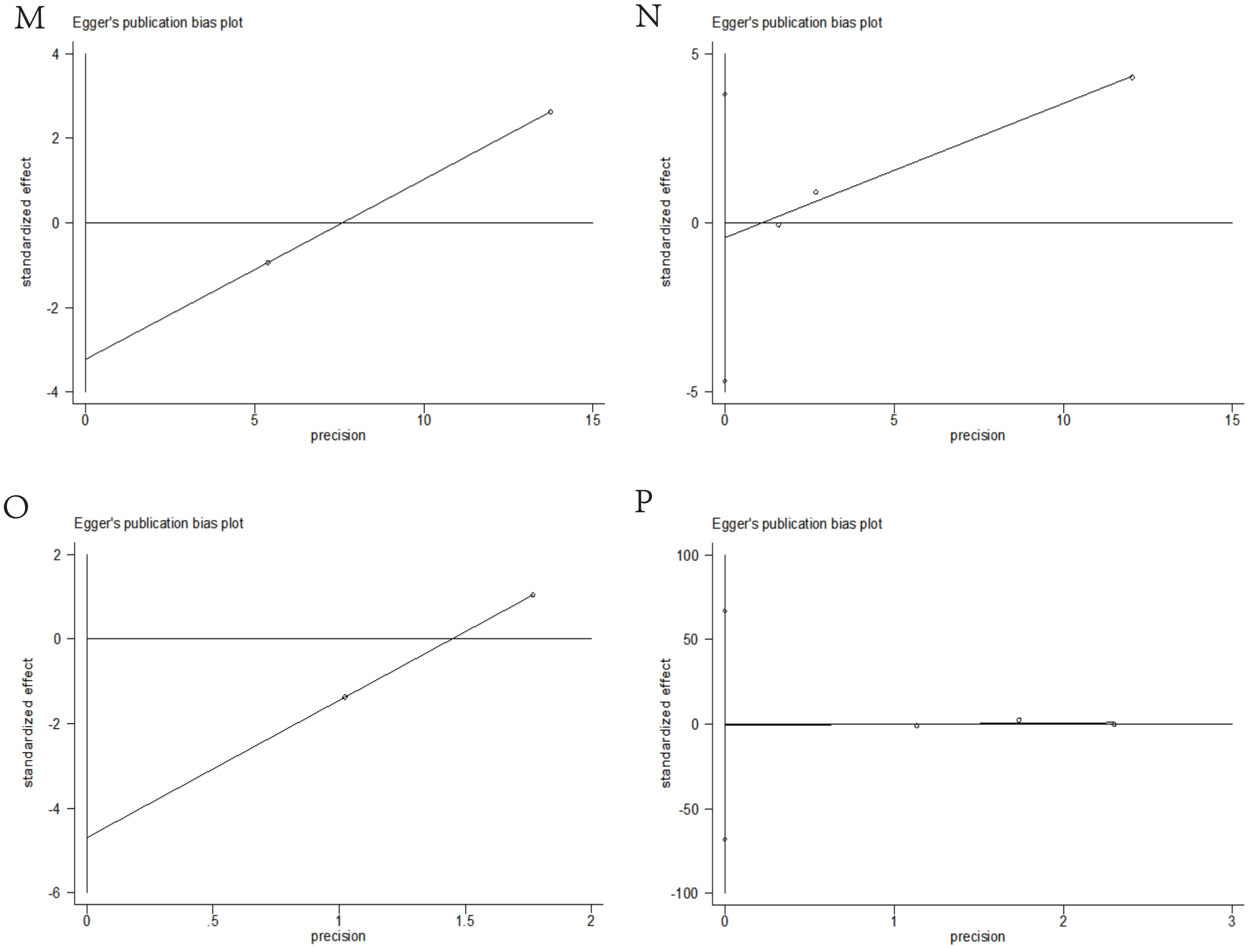
M
Egger's publication bias plot
standardized effect
precision
4
2
0
-2
-4
0
5
10
15
N
Egger's publication bias plot
standardized effect
precision
5
0
-5
0
5
10
15
O
Egger's publication bias plot
standardized effect
precision
2
0
-2
-4
-6
0
.5
1
1.5
2
P
Egger's publication bias plot
standardized effect
precision
100
50
0
-50
-100
0
1
2
3

Q

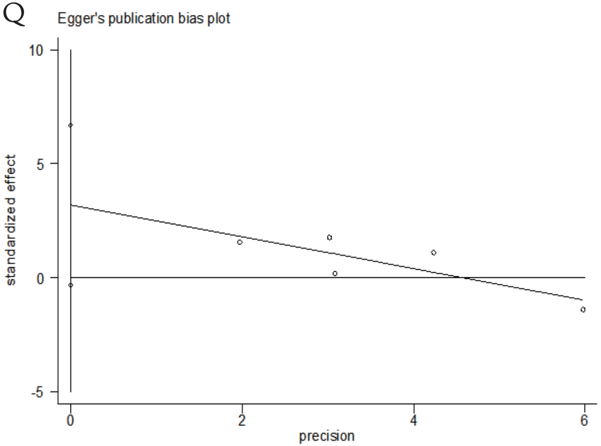

Supplementary Figure S6: Sensitivity analysis for cancers in RA patients with bDMARDs. Overall cancer(A), renal cancer(B), prostate cancer(C), ovarian cancer(D), breast cancer(E), cancer of uterus corpus(F), lymphoma(G), non-Hodgkin's lymphoma(H), Hodgkin's lymphoma(I), colorectal cancer(J), pancreas cancer(K), non-melanoma skin cancer(L), basal cell carcinoma(M), squamous cell carcinoma(N), brain and CNS cancer(O), oral cancer(P), lung cancer(Q).

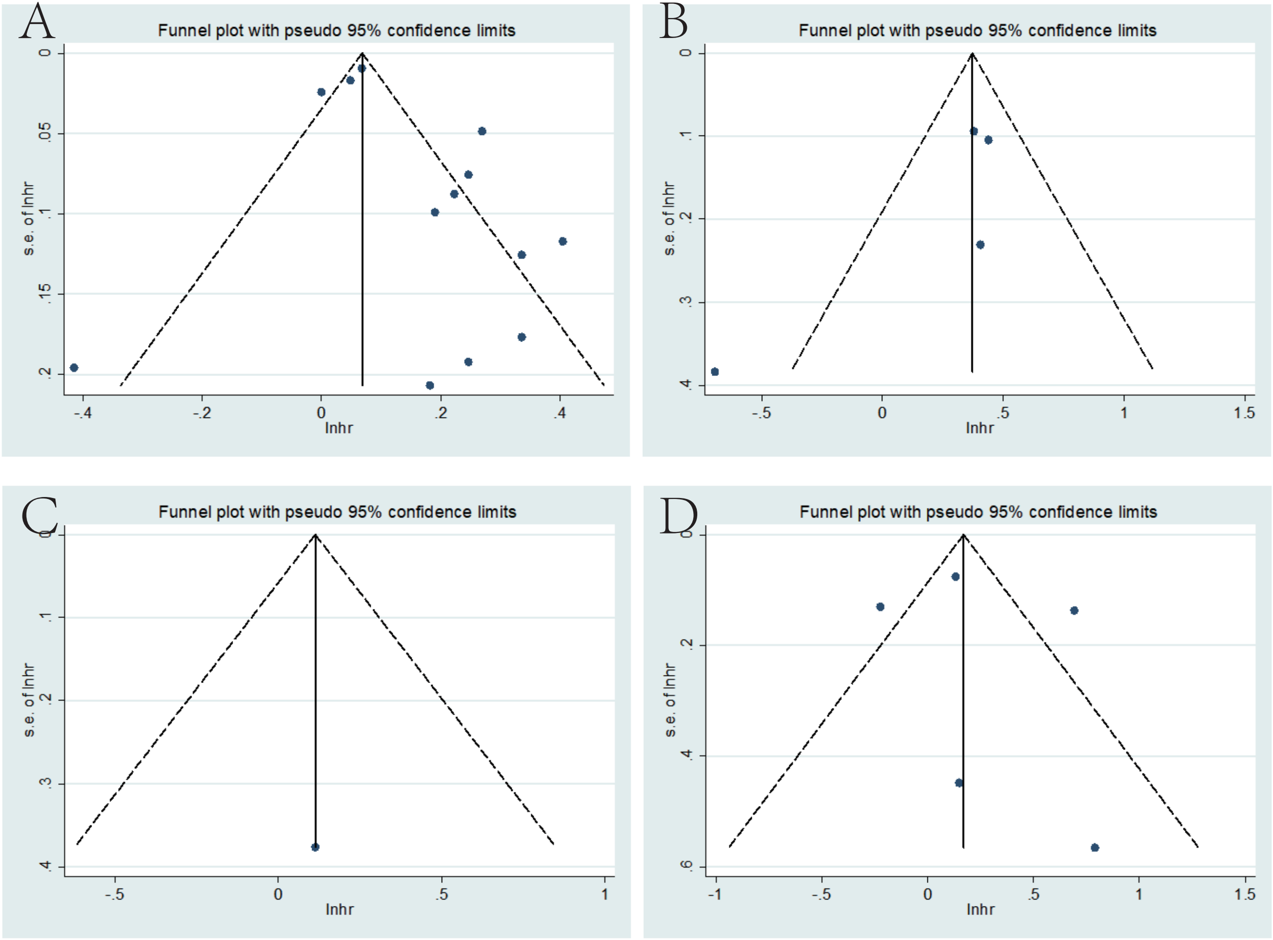

A
Funnel plot with pseudo 95% confidence limits
s.e. of lnhr
0
.05
.1
.15
.2
-.4
-.2
0
.2
.4
lnhr
B
Funnel plot with pseudo 95% confidence limits
s.e. of lnhr
0
.1
.2
.3
.4
-.5
0
.5
1
1.5
lnhr
C
Funnel plot with pseudo 95% confidence limits
s.e. of lnhr
0
.1
.2
.3
.4
-.5
0
.5
1
lnhr
D
Funnel plot with pseudo 95% confidence limits
s.e. of lnhr
0
.2
.4
.6
-1
-.5
0
.5
1
1.5
lnhr

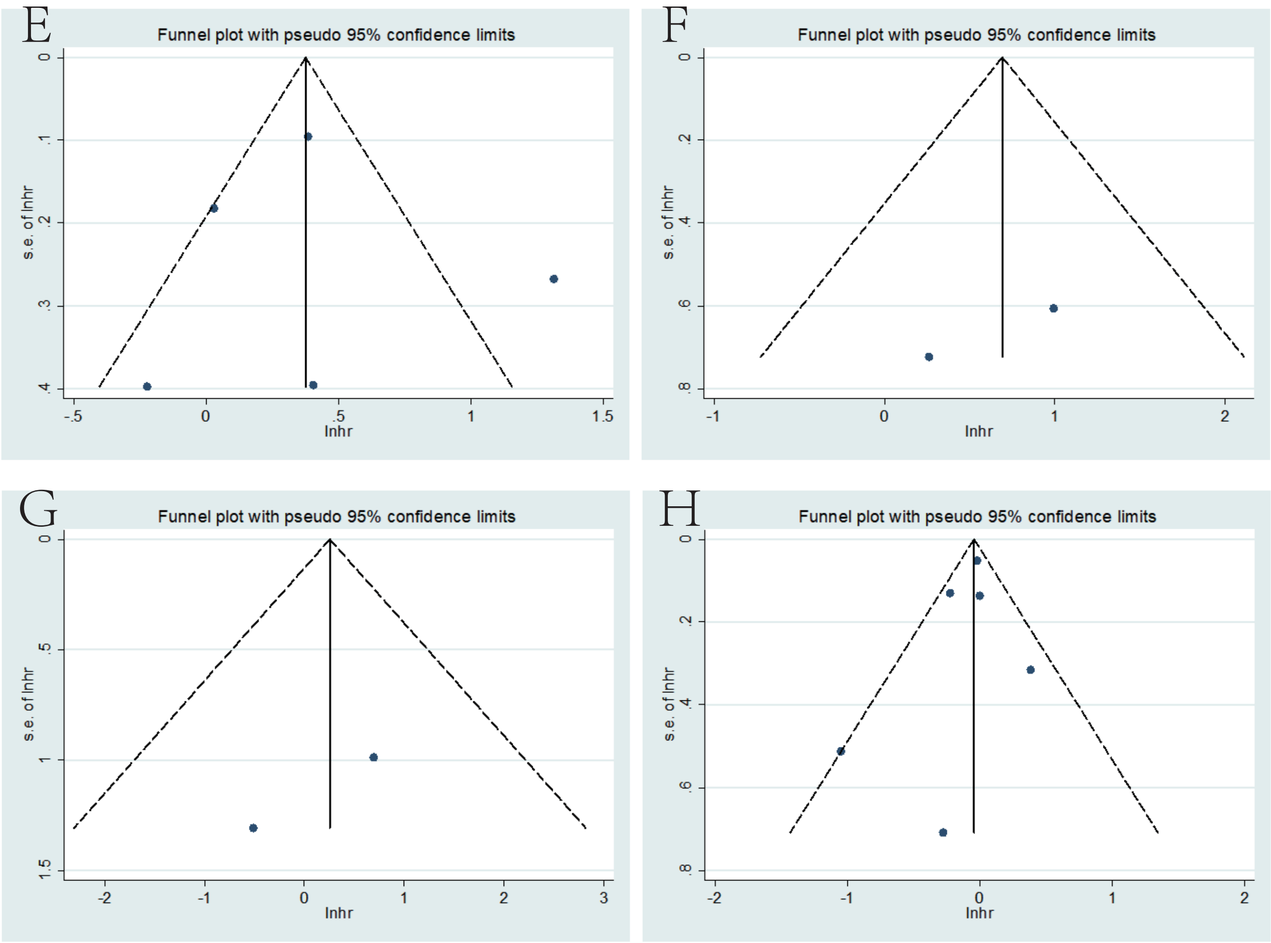

E
Funnel plot with pseudo 95% confidence limits
s.e. of lnhr
0
.1
.2
.3
.4
-.5
0
.5
1
1.5
lnhr
F
Funnel plot with pseudo 95% confidence limits
s.e. of lnhr
0
.2
.4
.6
.8
-1
0
1
2
lnhr
G
Funnel plot with pseudo 95% confidence limits
s.e. of lnhr
0
.5
1
1.5
-2
-1
0
1
2
3
lnhr
H
Funnel plot with pseudo 95% confidence limits
s.e. of lnhr
0
.2
.4
.6
.8
-2
-1
0
1
2
lnhr

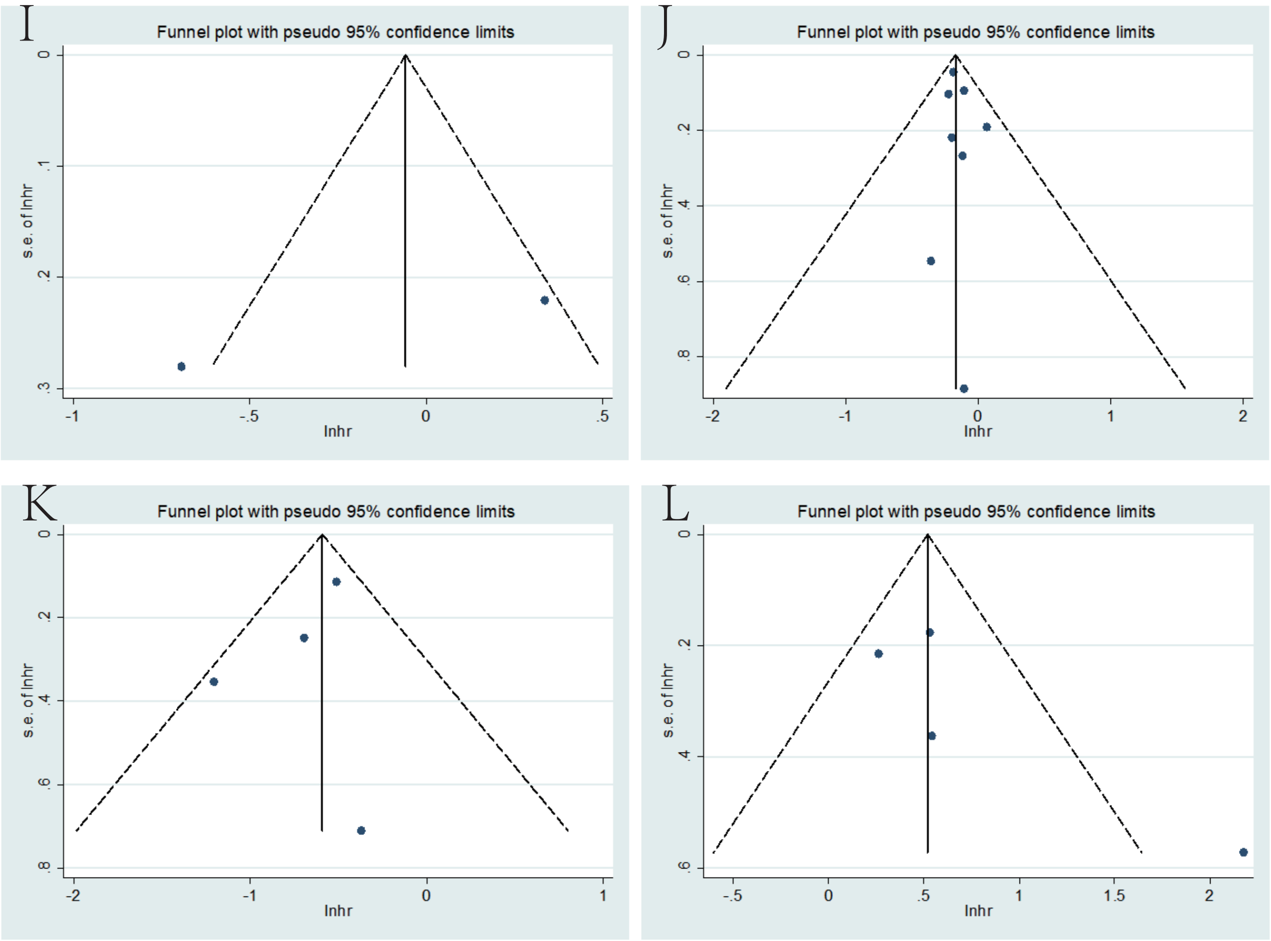

I
Funnel plot with pseudo 95% confidence limits
s.e. of lnhr
lnhr
J
Funnel plot with pseudo 95% confidence limits
s.e. of lnhr
lnhr
K
Funnel plot with pseudo 95% confidence limits
s.e. of lnhr
lnhr
L
Funnel plot with pseudo 95% confidence limits
s.e. of lnhr
lnhr

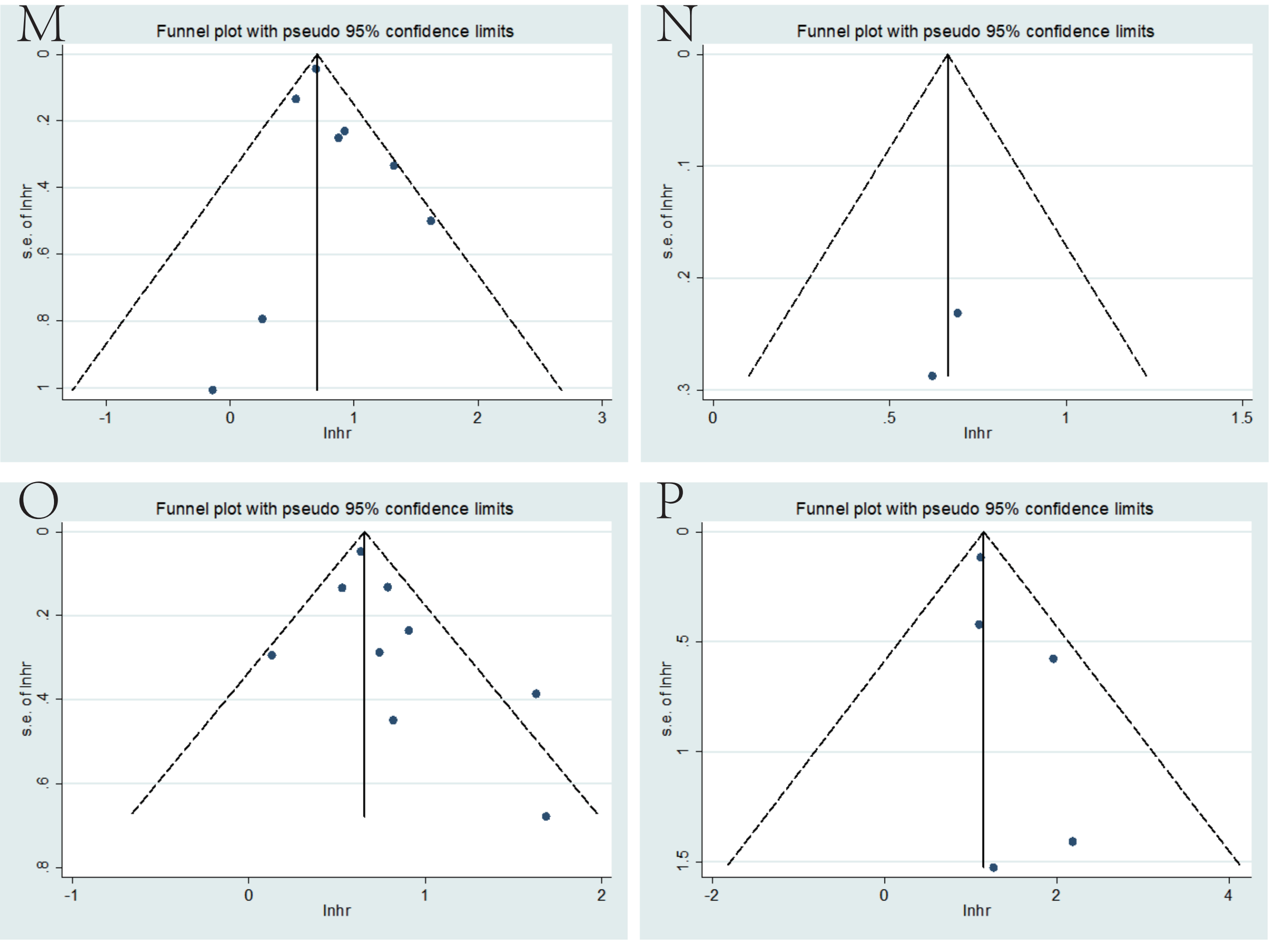
M
Funnel plot with pseudo 95% confidence limits
s.e. of lnhr
lnhr
N
Funnel plot with pseudo 95% confidence limits
s.e. of lnhr
lnhr
O
Funnel plot with pseudo 95% confidence limits
s.e. of lnhr
lnhr
P
Funnel plot with pseudo 95% confidence limits
s.e. of lnhr
lnhr

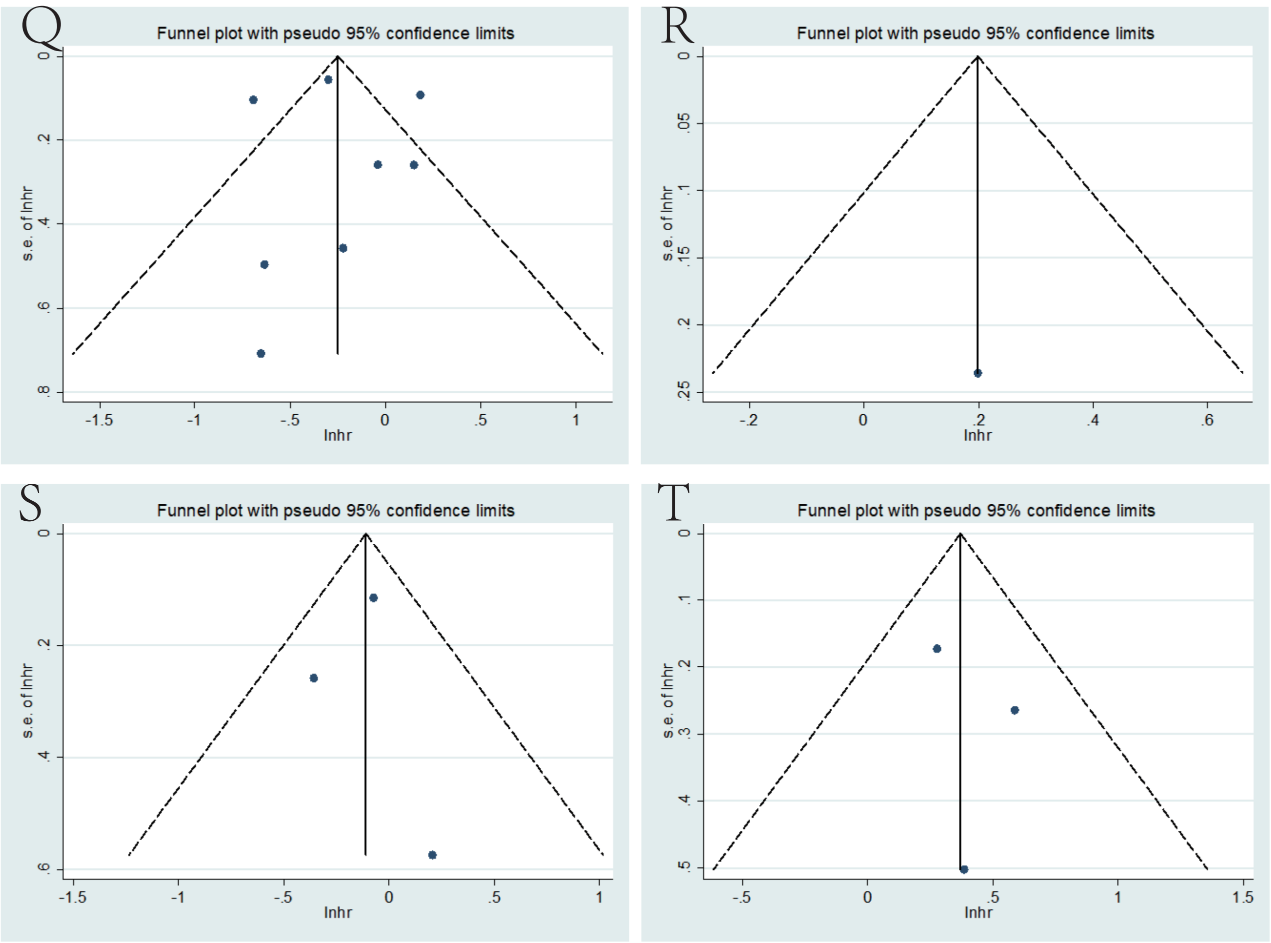

Q
Funnel plot with pseudo 95% confidence limits
s.e. of lnhr
lnhr
R
Funnel plot with pseudo 95% confidence limits
s.e. of lnhr
lnhr
S
Funnel plot with pseudo 95% confidence limits
s.e. of lnhr
lnhr
T
Funnel plot with pseudo 95% confidence limits
s.e. of lnhr
lnhr

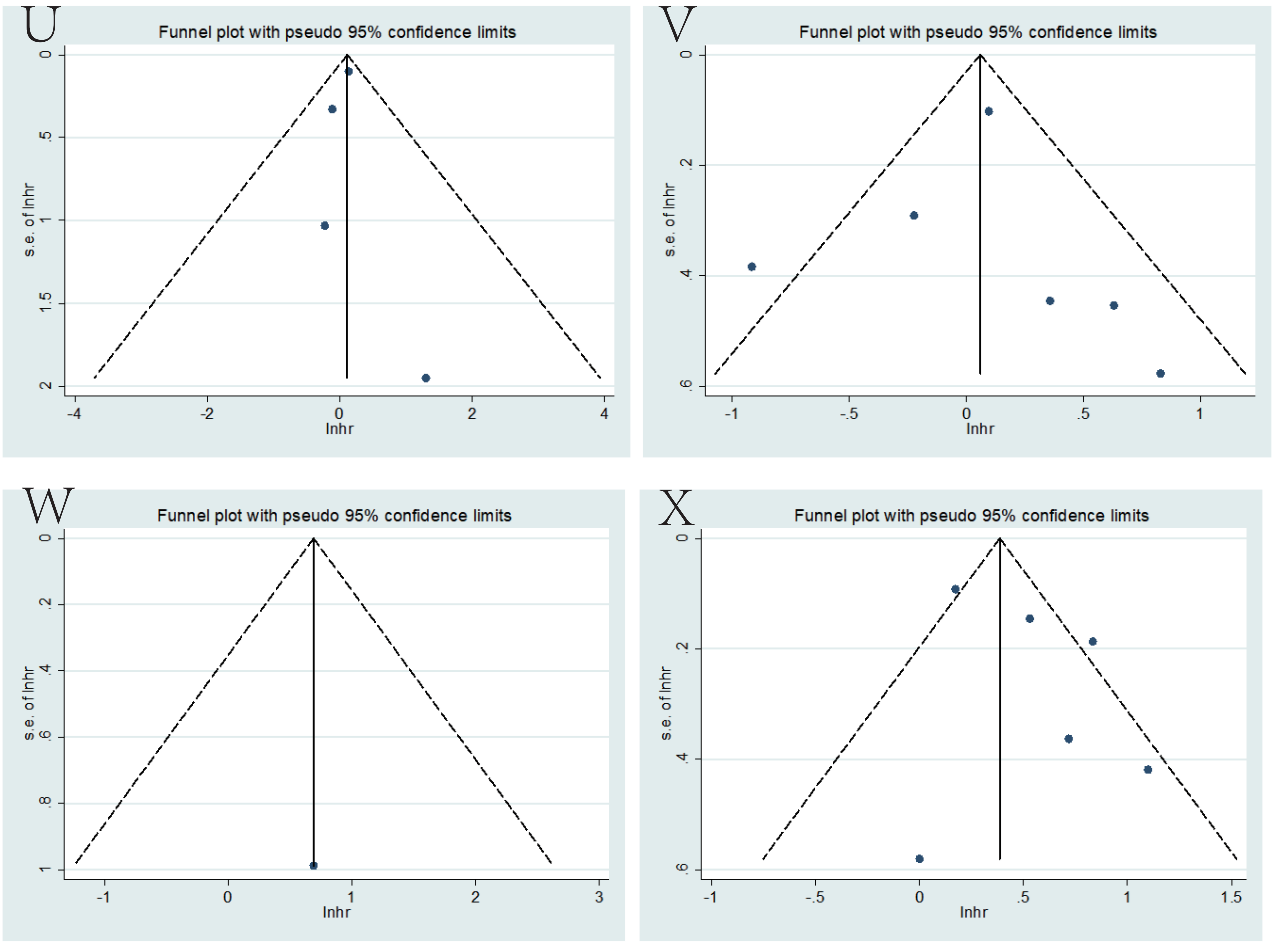
U
Funnel plot with pseudo 95% confidence limits
s.e. of lnhr
lnhr
V
Funnel plot with pseudo 95% confidence limits
s.e. of lnhr
lnhr
W
Funnel plot with pseudo 95% confidence limits
s.e. of lnhr
lnhr
X
Funnel plot with pseudo 95% confidence limits
s.e. of lnhr
lnhr

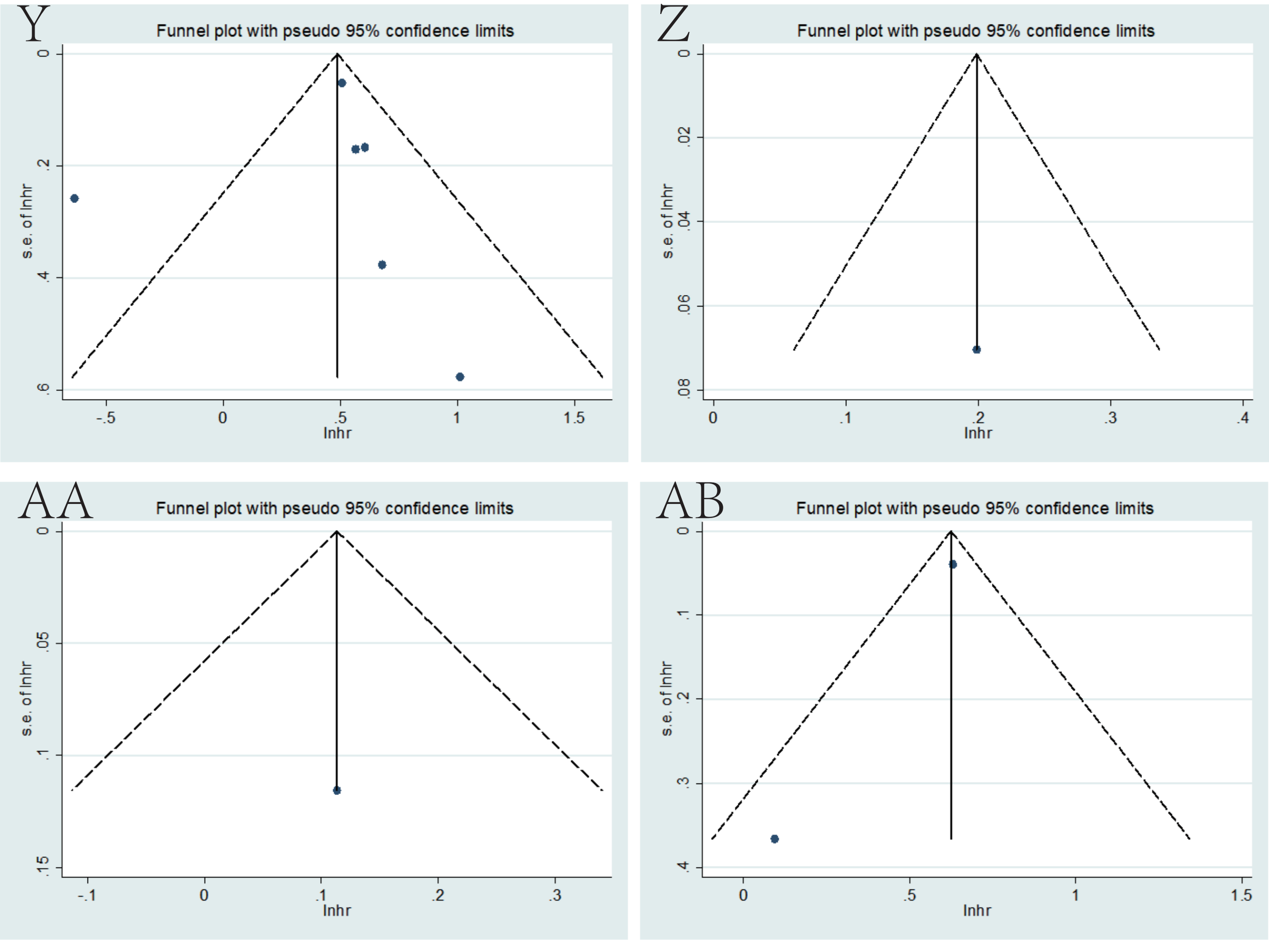
Y
Funnel plot with pseudo 95% confidence limits
s.e. of lnhr
0
.2
.4
.6
-.5
0
.5
1
1.5
lnhr
Z
Funnel plot with pseudo 95% confidence limits
s.e. of lnhr
0
.02
.04
.06
.08
0
.1
.2
.3
.4
lnhr
AA
Funnel plot with pseudo 95% confidence limits
s.e. of lnhr
0
.05
.1
.15
-.1
0
.1
.2
.3
lnhr
AB
Funnel plot with pseudo 95% confidence limits
s.e. of lnhr
0
.1
.2
.3
.4
0
.5
1
1.5
lnhr

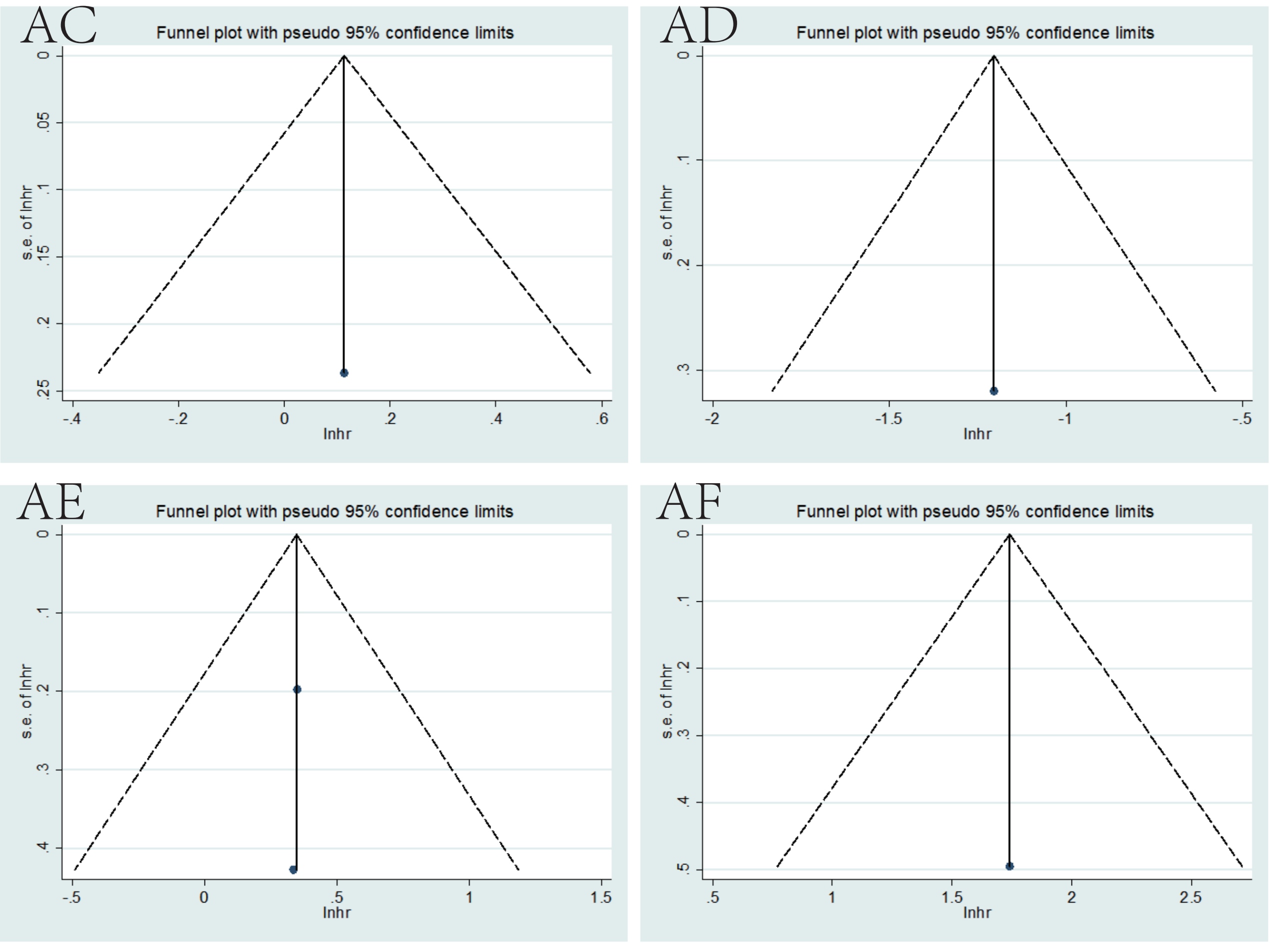

AC
Funnel plot with pseudo 95% confidence limits
s.e. of lnhr
0
.05
.1
.15
.2
.25
-.4
-.2
0
.2
.4
.6
lnhr
AD
Funnel plot with pseudo 95% confidence limits
s.e. of lnhr
0
.1
.2
.3
-2
-1.5
-1
-.5
lnhr
AE
Funnel plot with pseudo 95% confidence limits
s.e. of lnhr
0
.1
.2
.3
.4
-.5
0
.5
1
1.5
lnhr
AF
Funnel plot with pseudo 95% confidence limits
s.e. of lnhr
0
.1
.2
.3
.4
.5
.5
1
1.5
2
2.5
lnhr

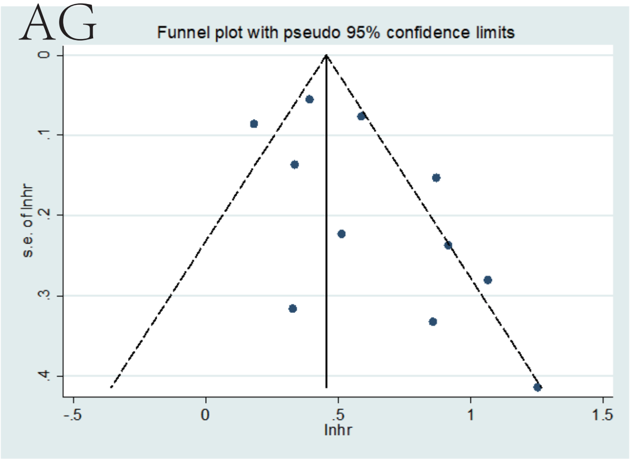

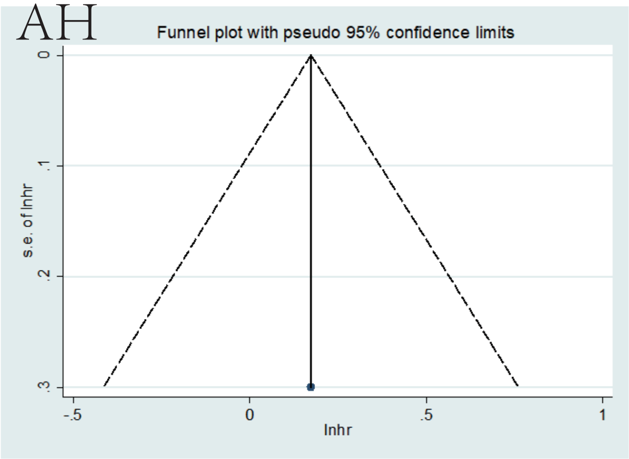

Supplementary Figure S7: Sensitivity analysis for cancers in RA patients with csDMARDs. Overall cancer (A), renal cancer (B), cancer of urethra (C), bladder cancer (D), cervical cancer (E), cancer of vulva and vagina (F), cancer of testis (G), prostate cancer (H), ovarian cancer (I), breast cancer (J), cancer of uterus corpus (K), leukemia (L), lymphoma (M), multiple myeloma (N), non-Hodgkin's lymphoma (O), Hodgkin's lymphoma (P), colorectal cancer (Q), cancer of small intestine (R), pancreas cancer (S), oesophageal cancer (T), liver cancer (U), gastric cancer (V), gallbladder cancer (W), melanoma (X), non-melanoma skin cancer (Y), basal cell carcinoma (Z), squamous cell carcinoma (AA), brain and CNS cancer (AB), oral cancer (AC), head and neck cancer (AD), cancer of soft tissues (AE), cancer of bones and joints (AF), lung cancer (AG), cancer of larynx (AH).

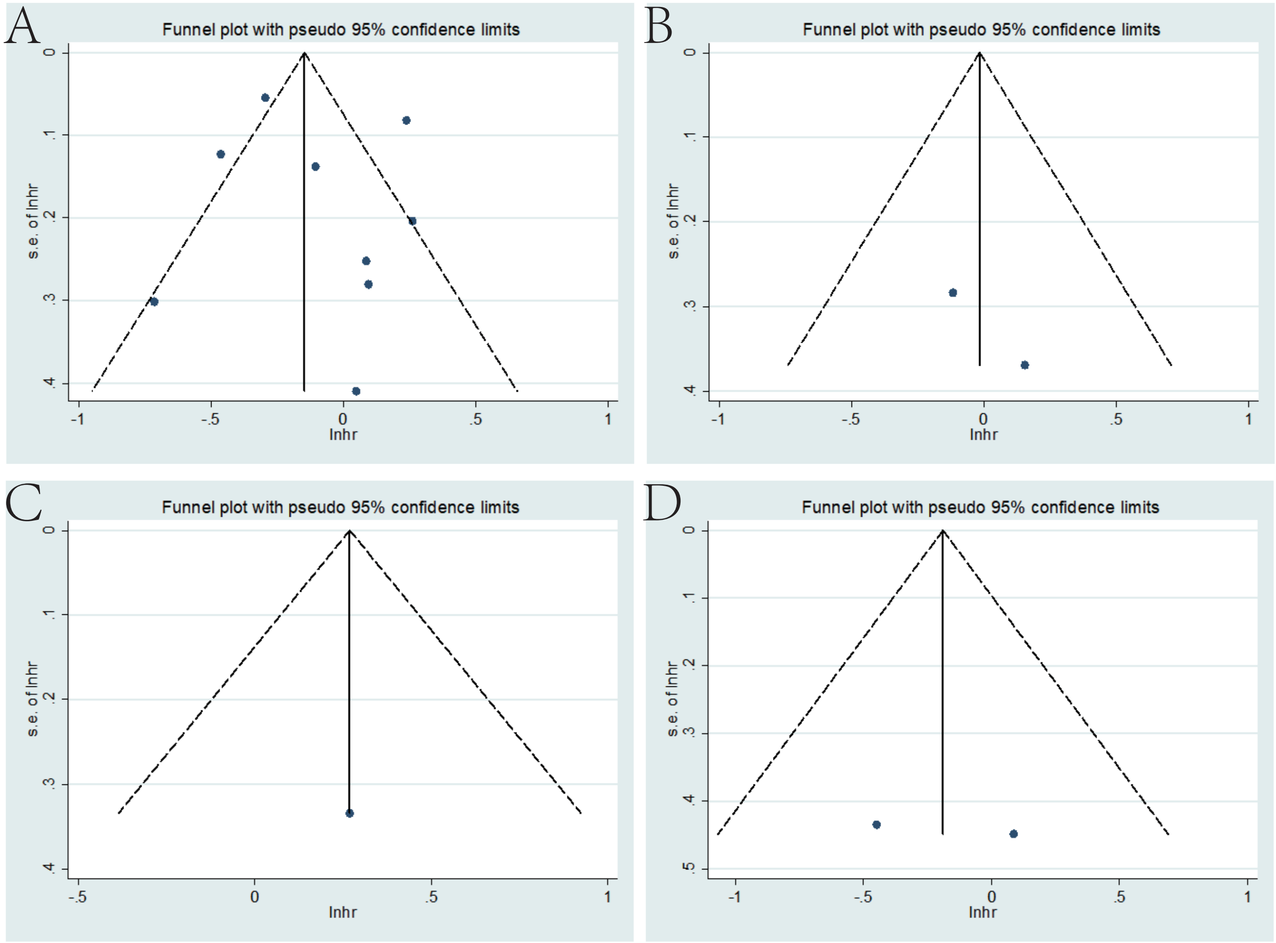

A
Funnel plot with pseudo 95% confidence limits
s.e. of lnhr
lnhr
B
Funnel plot with pseudo 95% confidence limits
s.e. of lnhr
lnhr
C
Funnel plot with pseudo 95% confidence limits
s.e. of lnhr
lnhr
D
Funnel plot with pseudo 95% confidence limits
s.e. of lnhr
lnhr

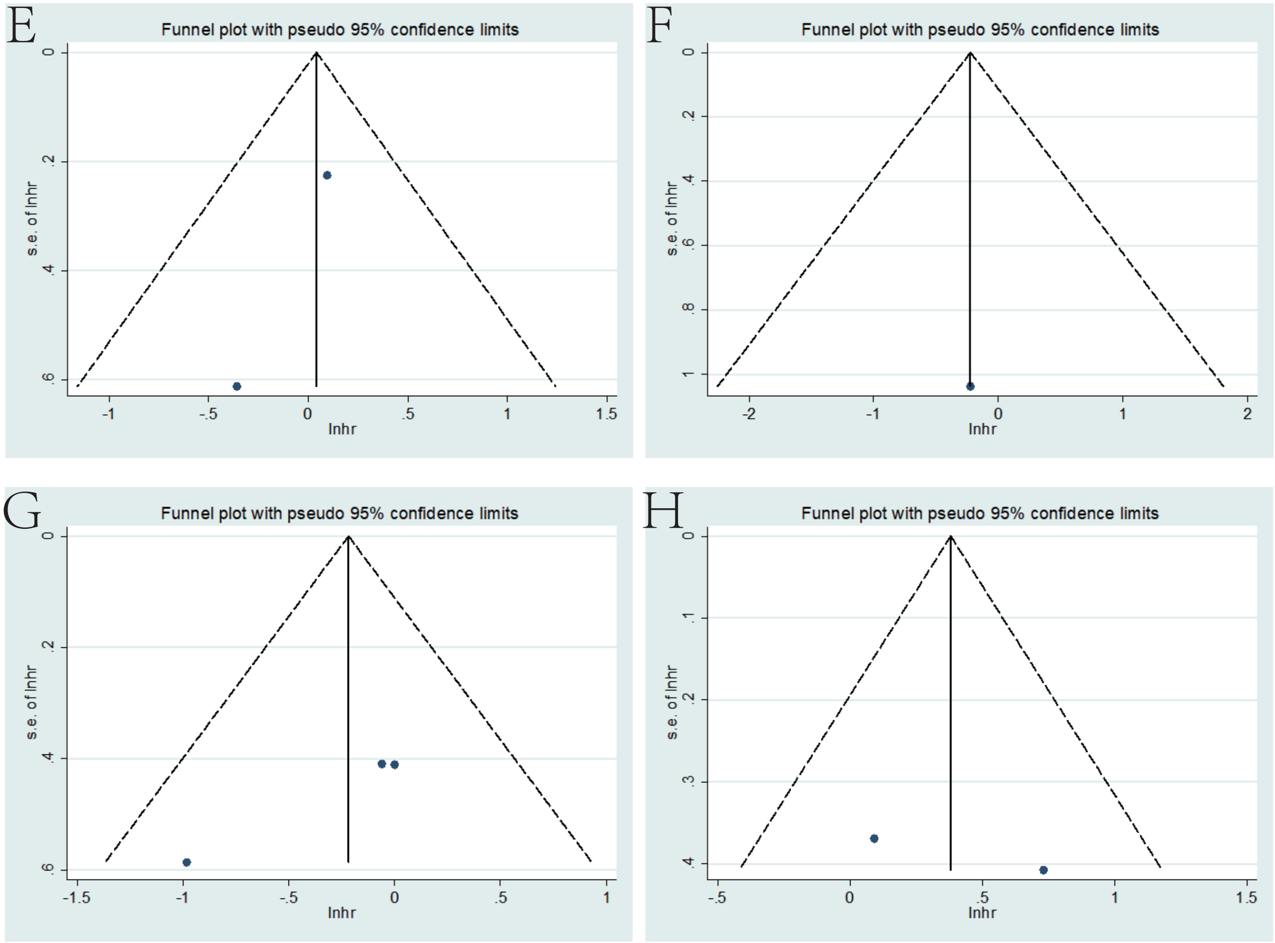
E
Funnel plot with pseudo 95% confidence limits
s.e. of lnhr
lnhr
F
Funnel plot with pseudo 95% confidence limits
s.e. of lnhr
lnhr
G
Funnel plot with pseudo 95% confidence limits
s.e. of lnhr
lnhr
H
Funnel plot with pseudo 95% confidence limits
s.e. of lnhr
lnhr

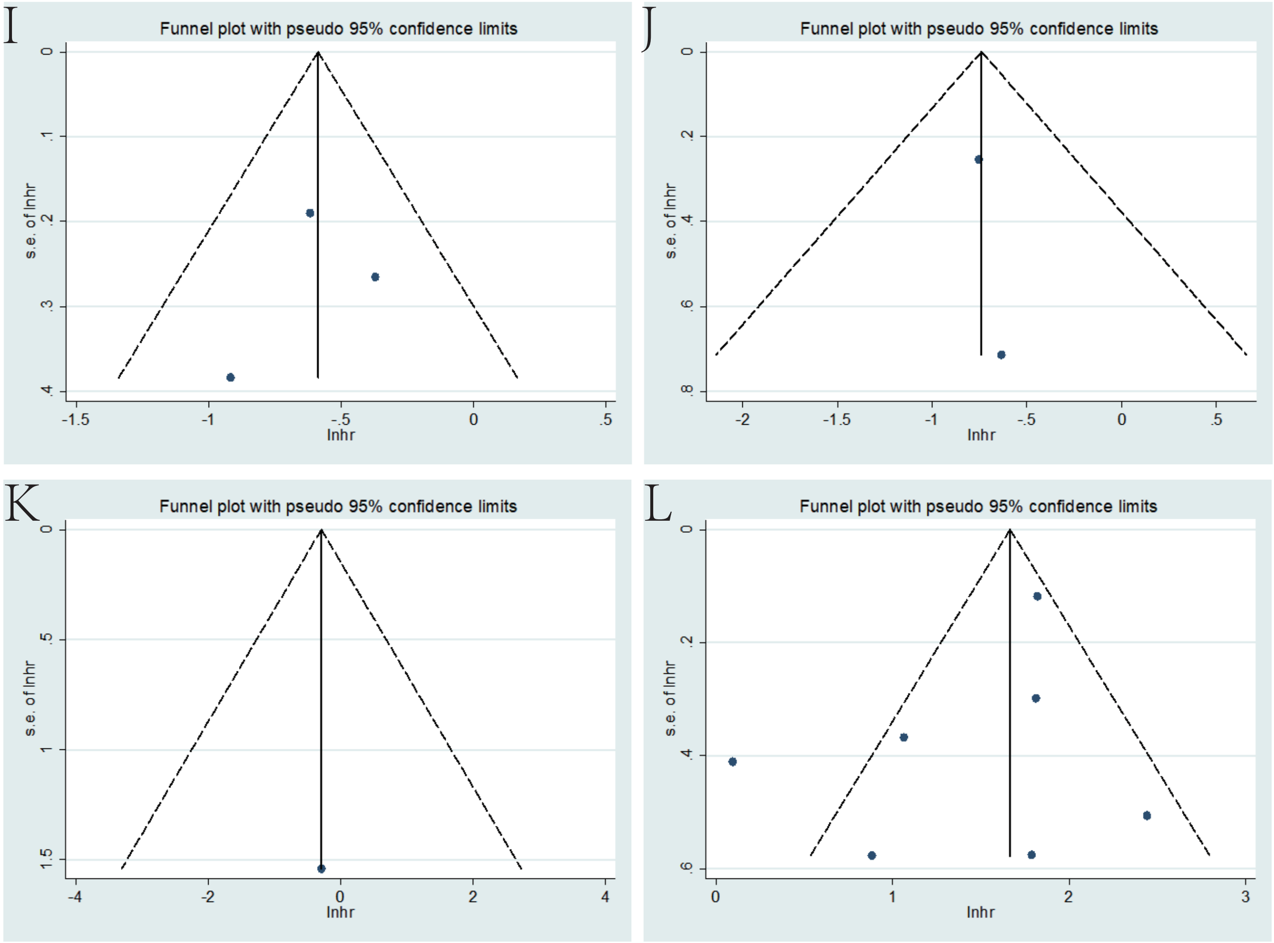
I
Funnel plot with pseudo 95% confidence limits
s.e. of lnhr
lnhr
J
Funnel plot with pseudo 95% confidence limits
s.e. of lnhr
lnhr
K
Funnel plot with pseudo 95% confidence limits
s.e. of lnhr
lnhr
L
Funnel plot with pseudo 95% confidence limits
s.e. of lnhr
lnhr

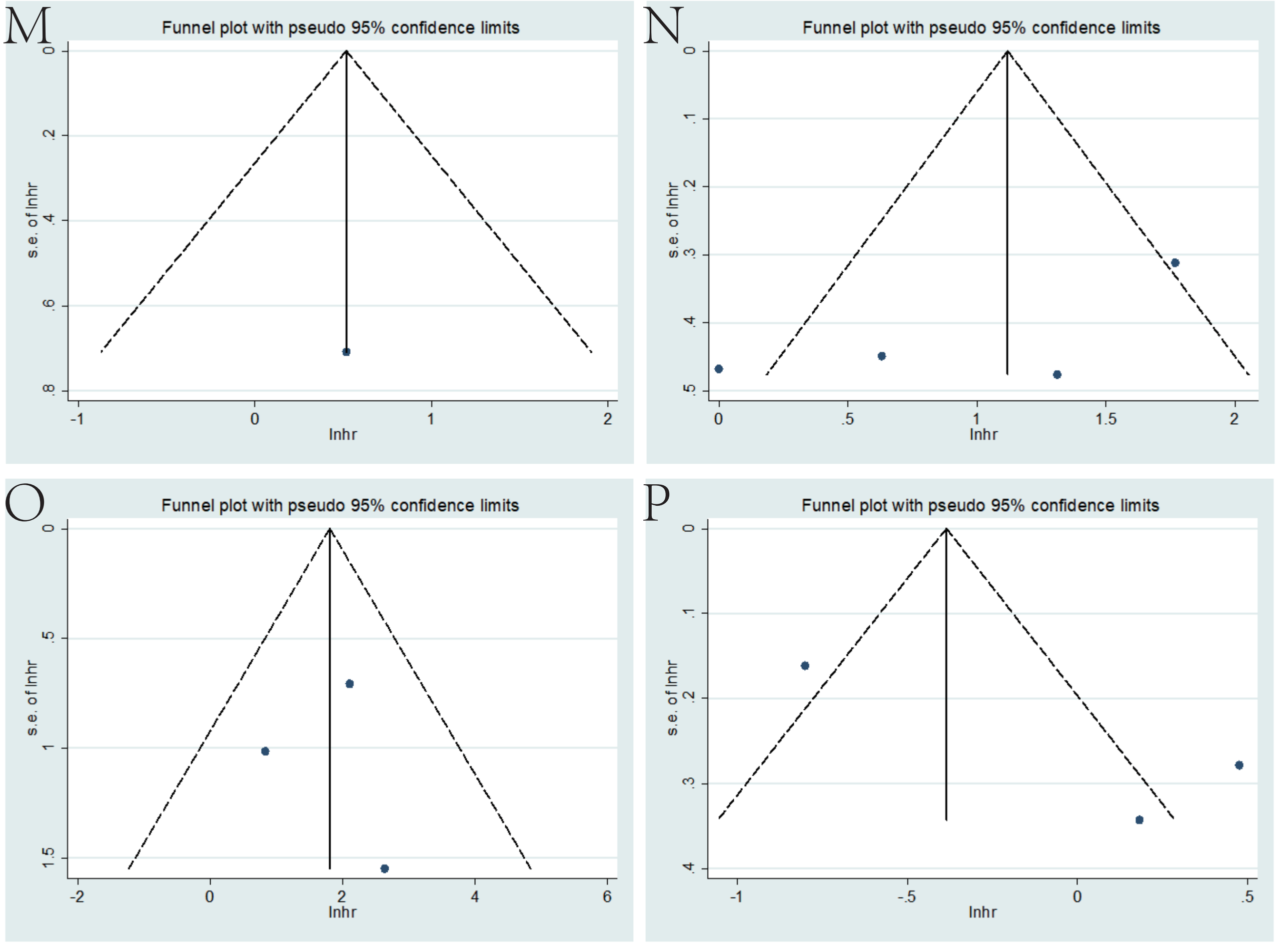
M
Funnel plot with pseudo 95% confidence limits
s.e. of lnhr
0
.2
.4
.6
.8
-1
0
1
2
lnhr
N
Funnel plot with pseudo 95% confidence limits
s.e. of lnhr
0
.1
.2
.3
.4
.5
0
.5
1
1.5
2
lnhr
O
Funnel plot with pseudo 95% confidence limits
s.e. of lnhr
0
.5
1
1.5
-2
0
2
4
6
lnhr
P
Funnel plot with pseudo 95% confidence limits
s.e. of lnhr
0
.1
.2
.3
.4
-1
-.5
0
.5
lnhr

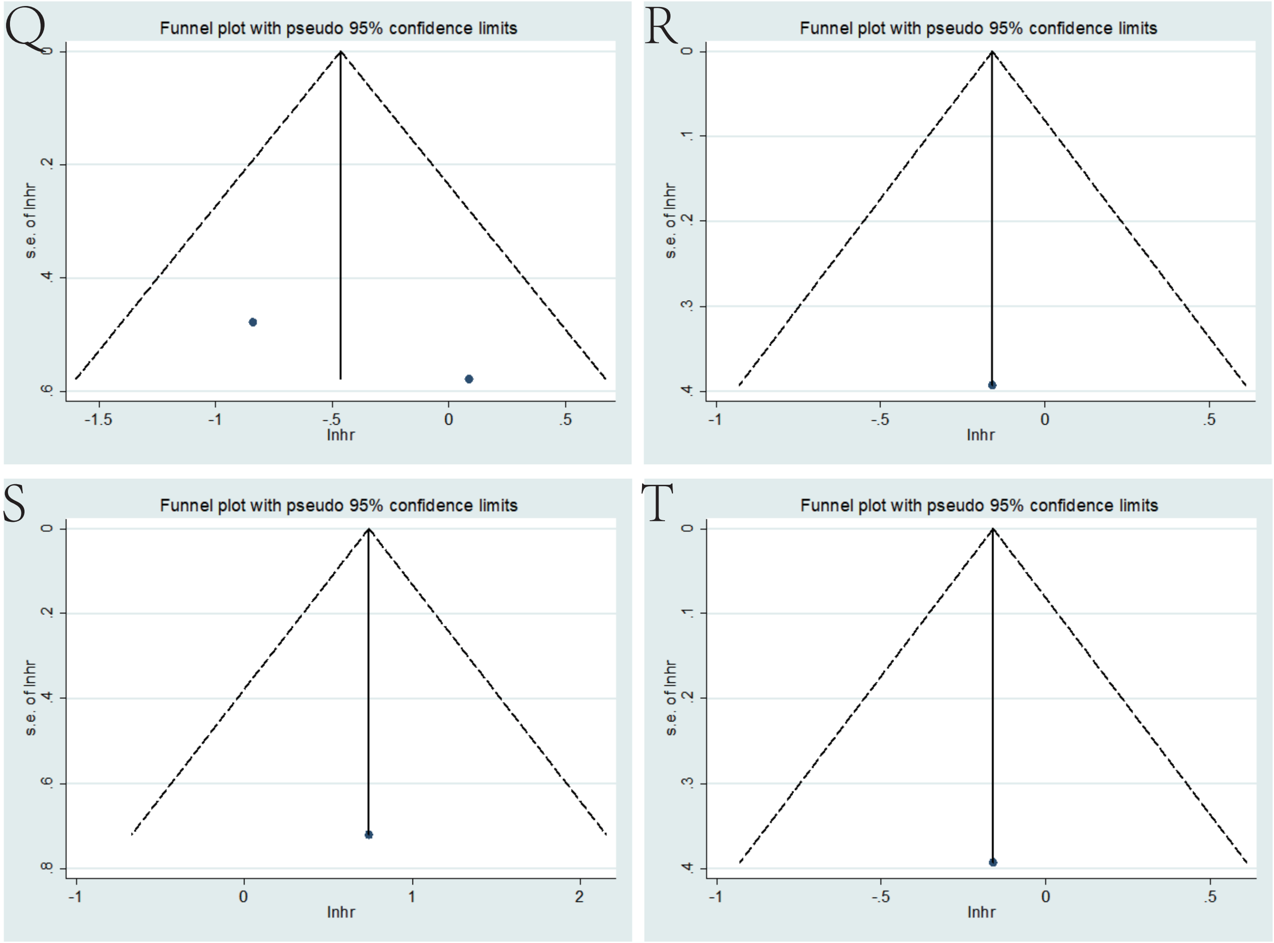
Q
Funnel plot with pseudo 95% confidence limits
s.e. of lnhr
0
.2
.4
.6
-1.5
-1
-.5
0
.5
lnhr
R
Funnel plot with pseudo 95% confidence limits
s.e. of lnhr
0
.1
.2
.3
.4
-1
-.5
0
.5
lnhr
S
Funnel plot with pseudo 95% confidence limits
s.e. of lnhr
0
.2
.4
.6
.8
-1
0
1
2
lnhr
T
Funnel plot with pseudo 95% confidence limits
s.e. of lnhr
0
.1
.2
.3
.4
-1
-.5
0
.5
lnhr

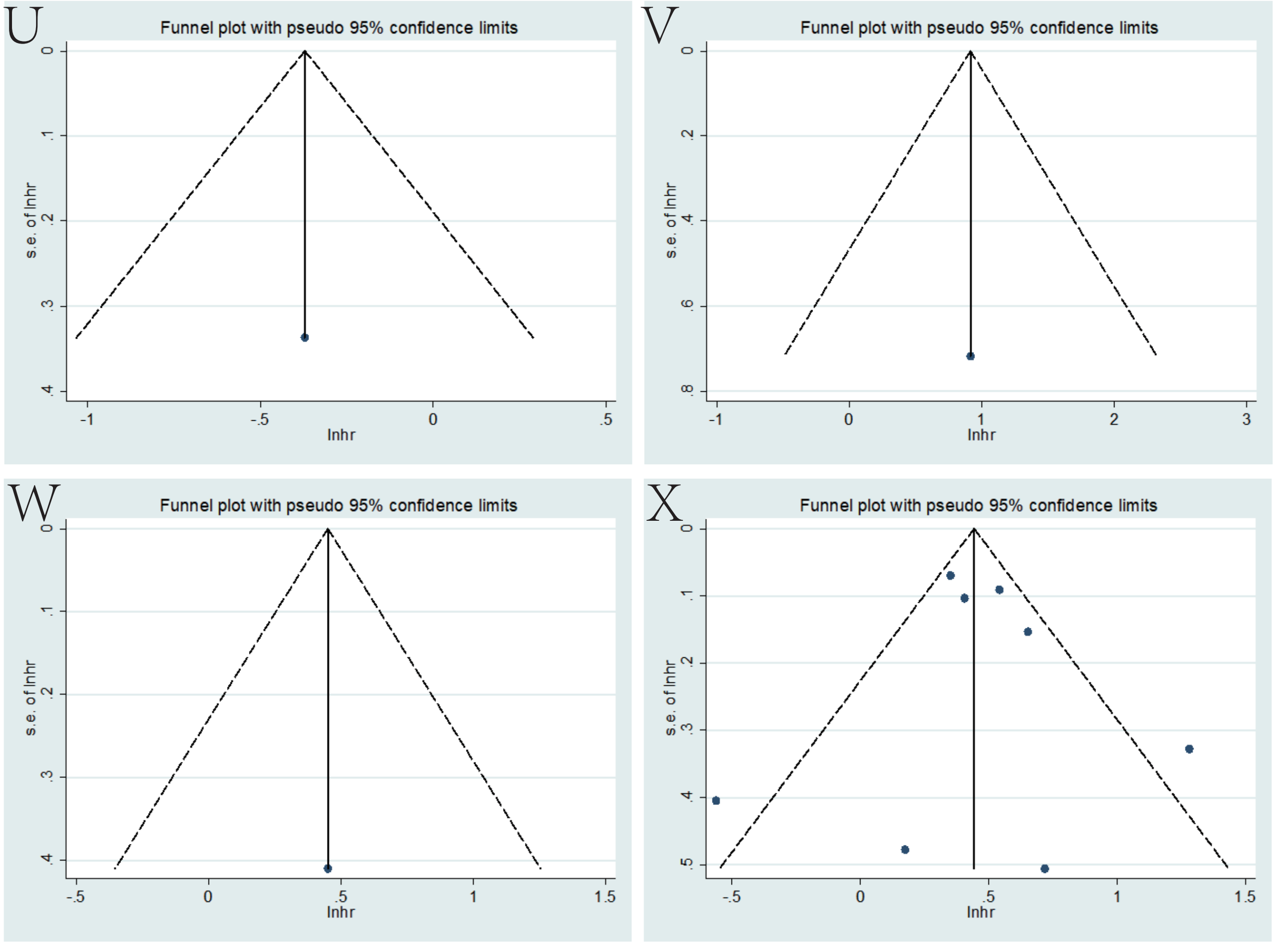
U
Funnel plot with pseudo 95% confidence limits
s.e. of lnhr
lnhr
V
Funnel plot with pseudo 95% confidence limits
s.e. of lnhr
lnhr
W
Funnel plot with pseudo 95% confidence limits
s.e. of lnhr
lnhr
X
Funnel plot with pseudo 95% confidence limits
s.e. of lnhr
lnhr

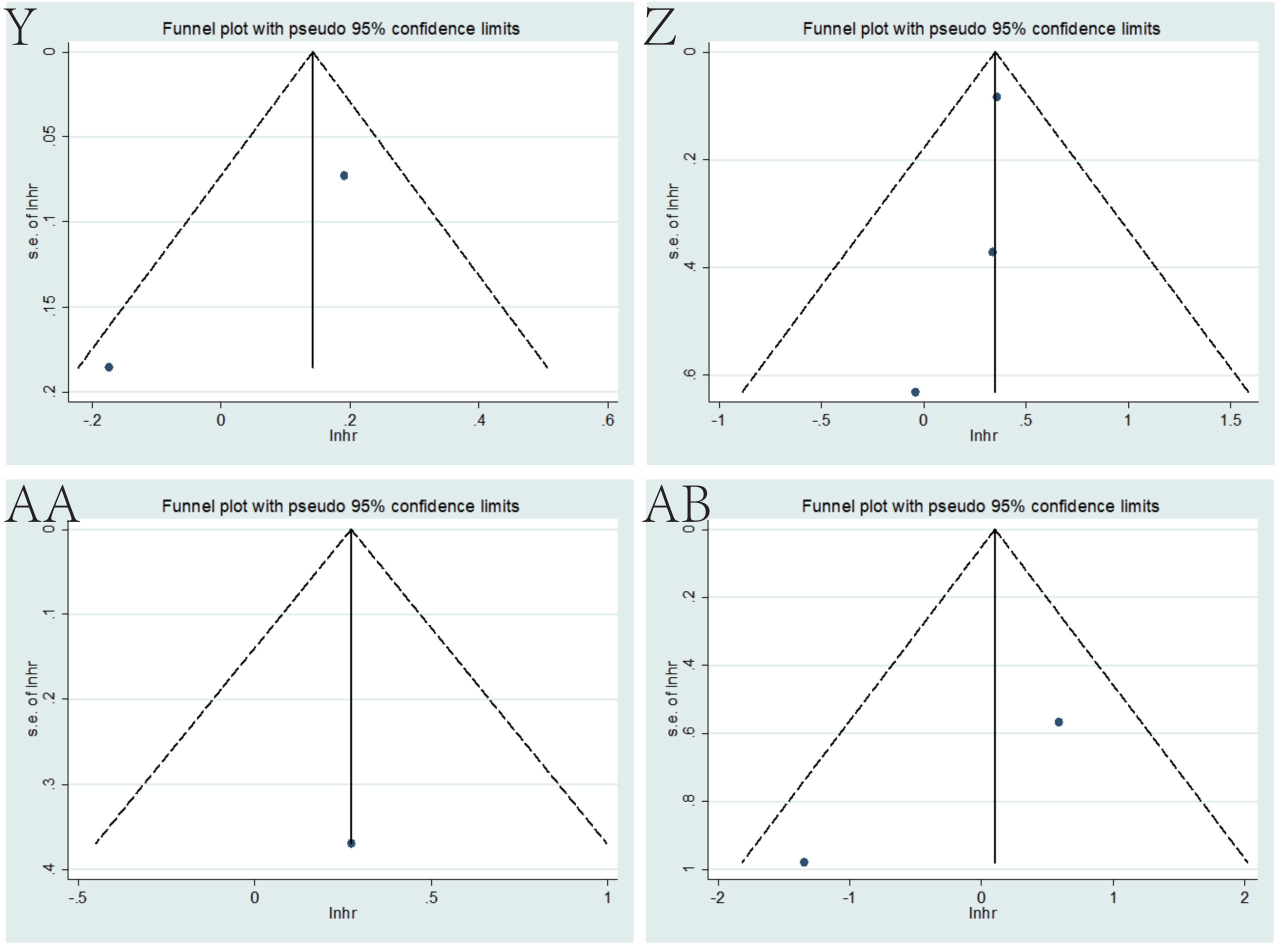

Y
Funnel plot with pseudo 95% confidence limits
s.e. of lnhr
0
.05
.1
.15
.2
-.2
0
.2
.4
.6
lnhr
Z
Funnel plot with pseudo 95% confidence limits
s.e. of lnhr
0
.2
.4
.6
-1
-.5
0
.5
1
1.5
lnhr
AA
Funnel plot with pseudo 95% confidence limits
s.e. of lnhr
0
.1
.2
.3
.4
-.5
0
.5
1
lnhr
AB
Funnel plot with pseudo 95% confidence limits
s.e. of lnhr
0
.2
.4
.6
.8
1
-2
-1
0
1
2
lnhr

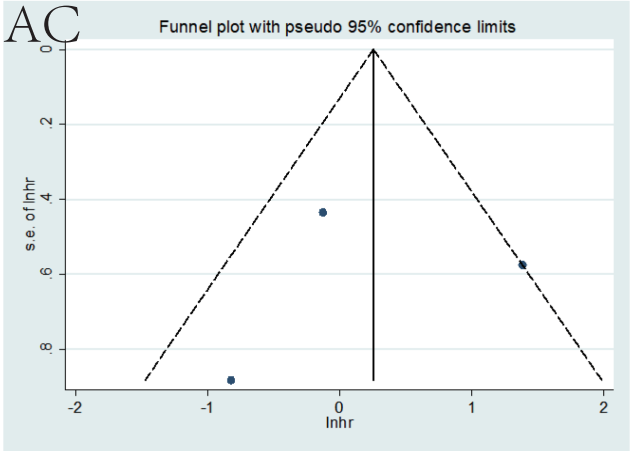

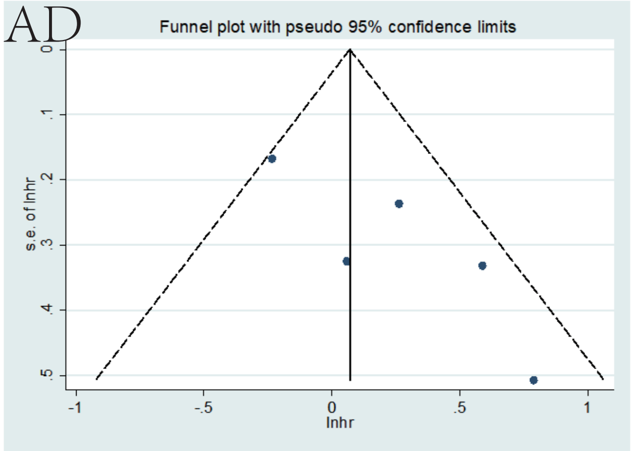

Supplementary Figure S8: Sensitivity analysis for cancers in RA patients with bDMARDs. Overall cancer (A), renal cancer (B), cancer of urethra (C), bladder cancer (D), cervical cancer (E), cancer of vulva and vagina (F), prostate cancer (G), prostate cancer (H), ovarian cancer (I), breast cancer (J), cancer of uterus corpus (K), leukemia, lymphoma (L), multiple myeloma (M), non-Hodgkin's lymphoma (N), Hodgkin's lymphoma (O), colorectal cancer (P), pancreas cancer (Q), oesophageal cancer (R), liver cancer (S), gastric cancer (T), cancer of bile duct (U), anus cancer (V), melanoma (W), non-melanoma skin cancer (X), basal cell carcinoma (Y), squamous cell carcinoma (Z), thyroid cancer (AA), brain and CNS cancer (AB), oral cancer (AC), lung cancer (AD).

Supplementary Table S1. Tumor Mutational Burden values and their natural logarithms of directly matched malignancies.

Site	Median TMB	ln Median TMB
Thyroid	1.8	0.59
Leukemia	1.8	0.59
Kidney	2.7	0.99
Testis	2.7	0.99
Liver	3.1	1.13
Breast	3.2	1.16
Prostate	3.3	1.19
Multiple Myeloma	3.3	1.19
Gallbladder	3.6	1.28
Cervix	4.5	1.50
Esophagus	5.0	1.61
Stomach	5.0	1.61
Anus	5.4	1.69
Bladder	6.6	1.89
Melanoma	13.5	2.60
Squamous Cell Carcinoma	45.2	3.81
Basal Cell Carcinoma	47.3	3.86
Head and Neck	5.0	1.61
Uterus Endometrial	4.5	1.50

TMB=Tumor Mutational Burden; ln=natural logarithm

All Median TMB values were extracted from the study of Chalmers et al.[1].

1. Chalmers ZR, Connelly CF, Fabrizio D, et al. Analysis of 100,000 human cancer genomes reveals the landscape of tumor mutational burden. Genome medicine. 2017 Apr 19;9(1):34.

Supplementary Table S2. Tumor Mutational Burden values and their natural logarithms of non-directly matched malignancies.

Disease Type	Averaged Median TMB	Median TMB	ln Averaged Median TMB
**Lung Cancer**	**6.60**		**1.89**
lung large cell carcinoma		12.2	
lung small cell undifferentiated carcinoma		9.9	
lung large cell neuroendocrine carcinoma		9.9	
lung squamous cell carcinoma		9.0	
lung non-small cell lung carcinoma		8.1	
lung sarcomatoid carcinoma		7.2	
lung adenocarcinoma		6.3	
lung adenosquamous carcinoma		5.4	
lung atypical carcinoid		1.8	
soft tissue rhabdomyosarcoma alveolar		1.7	
lung adenoid cystic carcinoma		1.3	
**Non-Hodgkin's Lymphoma**	**7.20**		**1.97**
lymph node lymphoma diffuse large B cell		10.0	
lymph node lymphoma follicular lymphoma		8.3	
lymph node lymphoma mantle cell		3.3	
**Brain/Central Nervous System**	**2.00**		**0.69**
brain gliosarcoma		3.6	
brain glioblastoma		2.7	
brain oligodendroglioma		2.7	
brain ependymoma		1.8	
brain meningioma		1.8	
brain medulloblastoma		1.8	
brain oligoastrocytoma		1.8	
brain astrocytoma		1.8	
brain anaplastic astrocytoma		1.8	
brain astrocytoma pilocytic		0.9	
**Skin**	**25.10**		**3.22**
skin basal cell carcinoma		47.3	
skin squamous cell carcinoma		45.2	
skin merkel cell carcinoma		4.3	
skin adnexal carcinoma		3.6	
**Colorectum**	**4.40**		**1.49**
colon adenocarcinoma		4.5	
colon neuroendocrine carcinoma		3.7	
rectum squamous cell carcinoma		5.9	
rectum adenocarcinoma		3.6	
**Pancreas**	**2.40**		**0.88**
pancreas acinar cell carcinoma		2.7	
pancreas neuroendocrine carcinoma		2.7	
pancreas ductal adenocarcinoma		1.8	
**Ovary**	**3.03**		**1.11**
ovary epithelial carcinoma		3.6	
ovary carcinosarcoma		3.6	

ovary high grade serous carcinoma		3.6	
ovary endometrioid adenocarcinoma		3.6	
ovary serous carcinoma		2.7	
ovary mucinous carcinoma		2.7	
ovary clear cell carcinoma		2.7	
ovary granulosa cell tumor		1.8	
**Small Intestine**	**3.05**		**1.16**
small intestine adenocarcinoma		4.5	
small intestine gist		1.8	
small intestine carcinoid		0.9	
**Soft Tissue**	**2.80**		**1.03**
soft tissue malignant peripheral nerve sheath tumor		2.5	
soft tissue sarcoma undifferentiated		2.5	
soft tissue angiosarcoma		3.3	
**Urethra**	**6.30**		**1.84**
kidney urothelial carcinoma		5.4	
bladder urothelial (transitional cell) carcinoma		7.2	
ureter urothelial carcinoma		5.4	
unknown primary urothelial carcinoma		7.2	

TMB=Tumor Mutational Burden; ln=natural logarithm

Averaged Median TMBs in bold were calculated by averaging the Median TMBs of the subtypes of the malignancies from the study of Chalmers et al.[1].

1. Chalmers ZR, Connelly CF, Fabrizio D, et al. Analysis of 100,000 human cancer genomes reveals the landscape of tumor mutational burden. Genome medicine. 2017 Apr 19;9(1):34.

Supplementary Table S3.Demographic details of the included studies.

Study	Region	Included Population	Age Characteristics	Drug	Drug detail	Reported Malignancies	Male(%)	Participant Characteristics	NOS Scores
Geborek,P. 2005 (25)	Sweden	757	csDMARDs 64 (52–78)<br>bDMARDs 56 (53–78)	Multiple	Methotrexate<br>Sulfasalazine<br>Ciclosporin<br>Antimalarial drugs<br>Injectable gold<br>Auranofin<br>Cyclophosphamide<br>TNF-I	Multiple	csDMARDs 27<br>bDMARDs 24	The South Swedish Arthritis Treatment Group (SSATG) register (1999-2002)	9
Dreyer,Lene. 2015 (15)	Denmark	3,347	csDMARDs 61.2 (15–92)<br>bDMARDs 54.3 (15–87)	Multiple	Adalimumab 46%<br>Etanercept 44%<br>Infliximab 48%<br>TNF-I	Multiple	csDMARDs 26<br>bDMARDs 27	Danish Cancer Registry (2000-2008)	9
Dreyer,Lene. 2017 (26)	Denmark	282	65.5 (38–85)	bDMARDs	Infliximab<br>Etanercept<br>Adalimumab<br>Golimumab<br>Certolizumab<br>Rituximab<br>Abatacept<br>Tocilizumab	Multiple	31.5	DANBIO Registry (2000-2011)	8
Mercer,Louise K. 2011 (27)	UK	15,510	csDMARDs 60 (12)<br>bDMARDs 56 (12)	Multiple	Etanercept<br>Infliximab<br>Adalimumab	Skin cancer	csDMARDs 28<br>bDMARDs 24	British Society for Rheumatology Biologics Register (2001-2008)	9
Ekström,Karin. 2003 (28)	Sweden	76,527	NA	csDMARDs	—	Multiple	29	Swedish nationwide a nd population-based registers (1964–1999)	8
Wolfe,Frederick. 2007 (29)	USA	13,001	58.5 (13.1)	Multiple	Prednisone 45.6%<br>Methotrexate 56.9%<br>Leflunomide 18.7%<br>Sulfasalazine 9.4%<br>Hydroxychloroquine 25.2%<br>Infliximab 19.9%<br>Etanercept 7.6%<br>Adalimumab 0.4%<br>Anakinra 0.3%	Multiple	22	US National Data Bank for Rheumatic Diseases (NDB) (1998-2005)	9
Raaschou,Pauline. 2015 (30)	Sweden	111,469	csDMARDs bDMARDs<br>SCC 60.9 (14.7) 55.2 (13.3)<br>BCC 61.6 (14.7) 55.3 (13.6)	Multiple	Adalimumab<br>Certolizumab pegol<br>Etanercept<br>Golimumab<br>Infliximab	Skin cancer (SCC,BCC)	csDMARDs bDMARDs<br>SCC 24.6 28.5<br>BCC 25.2 28.5	Sweden national administrative and clinical register (1998-2012)	9
Askling,J. 2005 (31)	Sweden	57,227	csDMARDs bDMARDs<br>16–44 8.8% 22.8%<br>45–74 56.3% 71.8%<br>>75 34.9% 5.4%	Multiple	TNF-I	Multiple	csDMARDs 28.6<br>bDMARDs 25.5	The Swedish Inpatient Register (1990-2003) Swedish national registries (1999-2003)	9

Pallavicini, Francesca Bobbio. 2010 (32)	Italy	1,064	55.84	bDMARDs	Etanercept 22.7%<br>Adalimumab 28.5%<br>Infliximab 48.8%	Multiple	16.8	The American Rheumatism Association (1987)	9
Abásolo,Lydia. 2008 (33)	Spain	789	61 (13)	csDMARDs	Methotrexate<br>Azathioprine<br>Chlorambucil<br>Cyclophosphamide	Multiple	28	The EMECAR cohort (Estudio de la Morbilidad y Ex-presión Clínica de la Artritis Reumatoide) (1999-2005)	8
Solomon,Daniel H. 2014 (34)	USA	3,761	csDMARDs bDMARDs<br><45 12.9% 15.3%<br>45-64 49.8% 58.9%<br>>65 37.2% 25.7%	bDMARDs	TNF-I	Multiple	csDMARDs 24.2<br>bDMARDs 22.0	The CORRONA registry (2001-2010)	9
Calip,Gregory S. 2018 (35)	USA	947	csDMARDs 59 (52–69)<br>bDMARDs 55 (50–61)	Multiple	methotrexate<br>hydroxychloroquine<br>sulfasalazine<br>leflunomide<br>etanercept<br>NSAIDs	NHL	csDMARDs 31.4<br>bDMARDs 37.5	The Truven Health MarketScan Research Database (2010-2014)	8
Amari,Wassila. 2011 (36)	USA	19,200	62.9 (12.3)	Multiple	Methotrexate 57.2%<br>Leflunomide 15.7%<br>Hydroxychloroquine 48.4%<br>Sulfasalazine 28.8%<br>Infliximab 4.5%<br>Adalimumab 8.8%<br>Etanercept 14.5%	NMSC	90.7	Department of Veterans' Affairs (VA) national administrative databases (1998-2008)	8
Waljee,Akbar K. 2019 (37)	Denmark	1,219	NA	bDMARDs	Adalimumab<br>Certolizumab pegol<br>Etanercept<br>Golimumab<br>Infliximab	Multiple	NA	The Danish National Patient Registry and the Danish Cancer Registry (1999-2016)	9
Lim,Xin Rong. 2019 (38)	Singapore	1,117	NA	csDMARDs	—	Multiple	20.4	The Tan Tock Seng Hospital (TTSH) RA Registry (2001-2013)	9
Wu,Chun Ying. 2014 (39)	China	22,130	csDMARDs 53.89 (13.09)<br>bDMARDs 53.88 (13.08)	Multiple	Methotrexate (48.9) (93.2)<br>Sulfasalazine (48.2）(74.0)<br>Hydroxychloroquine (62.0) (79.4)<br>Glucocorticosteroids (72.7） (90.4)	Multiple	86.2	The Taiwan National Health Insurance Research Database (1997-2011)	9
Harigai,Masayoshi. 2016 (40)	Japan	14440	57	bDMARDs	Etanercept 53.3%<br>Fliximab 45.8%<br>Tocilizuma 20.4%<br>Adalimumab 15.8%<br>Abatacept 6.4%<br>Golimumab 1.4%	Multiple	19.9	the SafEty of biologics in Clinical Use in Japanese patients with RhEumatoid arthritis (SECURE) (2013)	9

Setoguchi,Soko.<br>2006 (41)	USA	8,458	csDMARDs 73.4 (6.2)<br>bDMARDs 71.4 ± 5.4	Multiple	Methotrexate 86.4%<br>bDMARD 13.6%	Multiple	csDMARDs 26.9<br>bDMARDs 24.7	Medicare beneficiaries enrolled in the Pharmaceutical Assistance Contract for the Elderly in Pennsylvania (1994-2004)<br>Medicare beneficiaries enrolled in the Pharmaceutical Assistance to the Aged and Disabled program or Medicaid in New Jersey (1994-2004)<br>All residents of British Columbia, Canada (1996-2003)	8
Buchbinder,Rachell.<br>2008 (42)	Australia	458	<40 15%<br>40–49 17%<br>50–59 28%<br>60–69 25%<br>>70 15%	csDMARDs	Methotrexate	Multiple	33	a cohort of all patients attending the community-based private practices of 6 rheumatologists in Melbourne, Australia (1986-1998)	9
Mercer,Louise K.<br>2013 (17)	UK	3,771	<55 31%<br>55-64 31%<br>65-74 29%<br>>75 12%	csDMARDs	—	—	28	the British Society for Rheumatology Biologics Register (2002-2009)	9
Cordtz,René.<br>2016 (43)	Denmark	11,677	csDMARDs 59.2 (48.6-68.7)<br>bDMARDs 56.0 (46.3-64.5)	Multiple	—	Multiple	0	the national Danish DANBIO registry (2000-2011)	9
Askling,J.<br>2005 (44)	Sweden	4,160	16–44 22.8%<br>45–74 71.8%<br>>75 5.4%	bDMARDs	Etanercept<br>Infliximab adalimumab	Haematopoietic Malignancies (Malignant lymphoma, Plasma cell neoplasms, Leukaemia)	25.2	the Swedish Nationwide and population based health and census registers (1999-2003)	9
